# Low Reproductive Rate Predicts Species Sensitivity to Habitat Loss: A Meta-Analysis of Wetland Vertebrates

**DOI:** 10.1371/journal.pone.0090926

**Published:** 2014-03-20

**Authors:** Pauline E. Quesnelle, Kathryn E. Lindsay, Lenore Fahrig

**Affiliations:** 1 Geomatics and Landscape Ecology Research Laboratory, Ottawa-Carleton Institute of Biology, Carleton University, Ottawa, Ontario, Canada; 2 Wildlife and Landscape Science, National Wildlife Research Centre, Environment Canada, Ottawa, Ontario, Canada; Universität Zurich, Switzerland

## Abstract

We tested the hypotheses that species with greater mobility and/or higher reproductive rates are less sensitive to habitat loss than species with lower mobility and/or reproductive rates by conducting a meta-analysis of wetland vertebrate responses to wetland habitat loss. We combined data from 90 studies conducted worldwide that quantified the relationship between wetland amount in a landscape and population abundance of at least one wetland species to determine if mobility (indexed as home range size and body length) and annual reproductive rate influence species responses to wetland loss. When analyzed across all taxa, animals with higher reproductive rates were less sensitive to wetland loss. Surprisingly, we did not find an effect of mobility on response to wetland loss. Overall, wetland mammals and birds were more sensitive to wetland loss than were reptiles and amphibians. Our results suggest that dispersal between habitat patches is less important than species’ reproductive rates for population persistence in fragmented landscapes. This implies that immigration and colonization rate is most strongly related to reproduction, which determines the total number of potential colonists.

## Introduction

Habitat loss is the primary threat to biodiversity worldwide [1], but species show wide variation in their responses to habitat loss. This variation is often attributed to differences in species traits [2]–[3]. However, most studies evaluating species responses to habitat loss have measured habitat amount as patch size [4], rather than evaluating the effects of habitat loss over the landscape (i.e. landscape scale study). In addition, most are limited to a narrow range geographical locations [5]. Moreover, understanding the effects of species traits on species responses to habitat loss is often (unavoidably) confounded by correlations or synergistic interactions among the traits themselves [6]. Therefore, we still do not know, in general terms, why some species or species groups are more sensitive to habitat loss than others [5], [7], [8].

Dispersal ability is generally considered an important species trait influencing species response to habitat loss. Species with greater movement ranges are predicted to have higher colonization rates because they are able to access more habitat in a landscape [9]. Moreover, since colonization rates are assumed to be correlated to immigration rates, local extinction probability is predicted to be lower for species with higher movement ranges, due to rescue of populations from low numbers by immigration. Metapopulation studies therefore typically assume that more mobile species should be less susceptible to habitat loss than less mobile species [10]–[13]. However, some empirical studies have found the opposite, that more mobile species are more sensitive to habitat loss, possibly because they incur higher dispersal mortality [14]–[16]. Therefore, the general relationship between mobility and tolerance to habitat loss is not clear.

Reproductive rate could also influence species responses to habitat loss, but it is not often considered, at least in empirical studies. Simulation studies suggest that reproductive rate has a much larger effect on the amount of habitat required for population persistence than the per capita rate of emigration [17] or dispersal ability [18]; lower reproductive rates are predicted to increase the amount of habitat required for population persistence. To our knowledge, there are only two empirical tests of this prediction in which habitat amount is measured at a landscape scale. Vance et al. [19] found that forest bird species with lower reproductive rates require more habitat for a 50% probability of occurrence than do forest birds with higher reproductive rates. Similarly, Holland et al. [20] found a negative association between reproductive rate and minimum habitat amount required for presence across a group of dead wood boring beetle species. These studies suggest that species with lower reproductive rates require more habitat in a landscape for population persistence than do species with higher reproductive rates. This is likely because for a given amount of habitat in a landscape, species with higher reproductive rates will rebound more quickly from population declines than species with lower reproductive rates. Moreover, a large number of offspring increases the number of potential colonists which increases colonization rates of unoccupied patches [21]. Therefore, we predicted that species with lower reproductive rates are more sensitive to habitat loss than species with higher reproductive rates.

The objective of this study was to determine the importance of mobility and reproductive rate in determining the responses of wetland species to wetland habitat loss. We used a meta-analytical approach to quantitatively synthesize the results of 90 studies conducted across the world that quantified the relationship between wetland amount in a landscape and wetland animal abundance. From these we obtained 426 responses to wetland loss for 220 wetland species including mammals, birds, reptiles and amphibians. We selected wetlands and wetland species for several reasons. First, wetlands are generally thought to function as metapopulations [22]–[24] and thus they provide an appropriate system to test whether mobility drives species responses to habitat loss. Moreover, wetland species are undergoing the largest wildlife population declines worldwide, primarily due to habitat loss [25], but are a relatively understudied ecological group in landscape ecology. Therefore, there is a need to provide a general synthesis on how wetland species are responding to wetland habitat loss, to prioritize landscape-scale conservation action. For example, if mobility is driving the response to habitat loss, the conservation focus should be on facilitating movement during high dispersal events or increasing connectivity in landscapes. In contrast, if reproductive rate is driving the response, the focus should be on supporting critical reproductive stages. To our knowledge, this is the first meta-analysis of population responses to habitat amount at the landscape scale (previous reviews were conducted at the patch scale), across taxa, and including the relationship to species’ mobility and reproductive rate.

## Methods

### Study Selection Criteria

**Figure 1:**
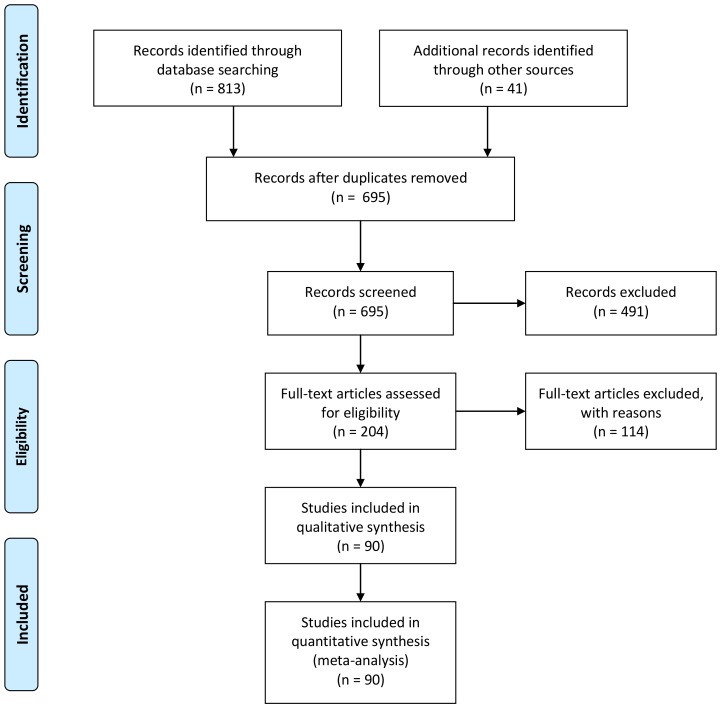
PRISMA literature search flow diagram.

We measured wetland loss as the amount of wetland in a landscape. We included studies that measured wetland amount as the percent of wetland area in a landscape (area-based buffers) or wetland connectivity (or isolation). We searched for studies that quantified the relationship between the amount of wetland in a landscape and population abundance of at least one wetland species in the Web of Science and ProQuest dissertation and theses databases on 01 December 2011 using the following keyword string: (wetland* OR marsh* OR swamp* OR pond*) AND (amount OR area OR isolat* OR fragmentation) AND (amphib* OR turtle* OR reptile* OR mammal* OR bird*) AND (abundance* OR occurrence* OR occup* OR distribution) AND (species OR population*) AND (landscape*). No restriction on date was used. We limited our analyses to empirical studies that were conducted in wetlands, including natural wetlands (e.g. pond, marsh) and artificially created wetlands (e.g. stormwater basins, rice fields). For all studies, we assumed the authors accurately selected wetland habitat for each species; however if wetland amount at the landscape scale included land cover types other than wetlands (e.g. lakes), we contacted authors for clarification or excluded the study from analyses. We used a broad definition of “population abundance” to include population size (or relative abundance), population density (or relative density) and species occupancy (as an index of low vs. high abundance). We defined “wetland species” as any vertebrate (mammal, bird, reptile or amphibian) that uses wetlands as primary habitat for at least one part of its life cycle. We included species complexes that were fertile hybrids (e.g. *Pelophylax esculentus*) or two species that could not be distinguished (e.g. larval stages of *Ambystoma* spp) as one species in the meta-analysis. During our literature search, we recorded the number of articles identified and the number of studies included and excluded according to the Preferred Reporting Items for Systematic Reviews and Meta-Analyses (PRISMA) Statement (Figure 1).

### Data Extraction

In our meta-analysis, an effect size represents the quantitative relationship between the amount of wetland in a landscape and population abundance for a given wetland species. To extract an effect size from each study, we first searched the paper for a test statistic for the effect of wetland amount on animal abundance, and/or summary statistics (e.g. mean and variance), and corresponding sample size that could be converted into an effect size. When these values were not reported, we calculated them using raw data if they were provided in the paper, or we could extract them from figures using GetData Graph Digitizer 2.25 (Fedorov, S. 2012, internet free software), or we could obtain them from the authors. When a single study reported results for more than one species, we entered each species effect size as an independent estimate. When a study combined abundance or occurrence data across species, such that values for individual species could not be extracted, we contacted the authors for raw data or excluded the study. We did not calculate an effect size for 36 (of 256) species that occurred in ≤10% of sampled landscapes or locations in each study, because effect size estimation is biased when there is a high proportion of absences [26]. While this may have resulted in exclusion of species sensitive to habitat loss, in the absence of sufficient sample sizes we cannot include them in the analyses. When a single study presented more than one effect size for a given species such that different effect sizes representing responses of the same species to different wetland types, we averaged these estimates across wetland types to extract a single effect size for that species, to avoid non-independence (4 studies). When a single study presented data in multiple years using the same study design, we averaged estimates across years for continuous data or tallied the numbers of years present for occupancy data. When studies presented effects of wetland amount at multiple landscape scales (buffer sizes), we selected the largest estimate, on the assumption that this scale was closest to the scale at which wetland loss best predicts the species’ response (i.e. scale of effect, *sensu* [27]).

### Study Design Moderators

We identified three study design moderators to test if differences in study design influenced the magnitude and direction of the effect size, and to statistically control for such effects in remaining analyses (Table 1). First, the effect size might vary depending on how wetland amount in a landscape was measured. We combined several measures of wetland amount, including simple area-based buffers and nearest-neighbour distances to more complex connectivity indexes based on the incidence function model, because these measures have been shown to be highly correlated and have similar performance in predicting ecological responses [28]–[34]. These comparative studies also suggest that measures with more information about the amount of (occupied) habitat in the landscape are better predictors, and therefore we expected a priori studies using such measures would have larger effect sizes. We distinguished two study types, 1) amount-based studies, where wetland amount was calculated as the percent wetland area in a landscape or buffer surrounding the sampled wetland patch, or 2) configuration-based studies, where the configuration of wetland habitat was included in the calculation of the measure, such as the number of wetland patches in a landscape, nearest-neighbour distances, wetland proximity or wetland connectivity. For all study types, we applied the convention that each effect size extracted from a study should represent the population response of a species to increasing wetland amount in a landscape. However, for nearest-neighbour studies, a negative effect of increasing distance indicates that a species responded positively to closer wetlands, or equivalently, greater wetland habitat amount within the surrounding landscape. Therefore, we reversed the sign of the effect sizes extracted for nearest-neighbour studies to make them comparable to those extracted for all other studies representing the response to increasing wetland amount [35].

**Table 1:** Study design and species trait moderator variables used in the meta-analysis.

Moderator Type	ModeratorVariable	Categoryor Range	Description
StudyDesign	Study Type	Amount-based	Wetland amount was measured as the percent wetland area in a landscape orbuffer (area-based buffers)
		Configuration-based	Wetland amount was measured as the number of wetland patches in a landscape,or using wetland isolation (nearest-neighbour distances) or connectivity(incidence function model) metrics
	Sampling Effort	Area-dependent	Sampling effort increased in proportion to the sampled wetland area
		Area-independent	Sampling effort was consistent across sampled wetlands
		Unknown	Sampling effort was unknown
	Patch Area	Included	Sampled wetland area was included in the calculation of wetlandamount in the landscape
		Not included	Sampled wetland area was not included in the calculation of wetlandamount in the landscape
SpeciesTrait	Home RangeSize	0.001–35600	Mean annual home range or territory size (ha)
	Body Length	2.5–1200	Mean body length across both sexes (cm)
	Reproductive rate	2–16000	Mean litter or clutch size multiplied by the mean number of litters orclutches per year

Second, the relationship between sampling effort used to measure population abundance of a given species and the size of wetlands may influence the effect size observed. Studies where sampling effort increased in proportion to wetland size will observe a positive relationship between wetland size and abundance, simply because more area is searched in larger wetlands. If wetland patch size is positively correlated with total wetland amount in a landscape, this will inflate the effect of wetland amount because a greater amount of wetland was sampled in landscapes containing more wetland. Therefore, we categorized studies by the sampling effort as, 1) area-independent, where sampling effort was consistent across sampled wetlands, 2) area-dependent, where sampling effort increased in proportion to the wetland area, or 3) unknown, where the sampling effort was unknown and could not be obtained by contacting authors. When a study used a combination of more than one of these methods, we selected the sampling method that accounted for the majority of the data.

Third, the effect of wetland habitat amount in a landscape could vary depending on whether the sampled wetland was included in the calculation of wetland area in a landscape (wetland amount). Prugh [31] found that measures of habitat amount in a landscape, including area-based buffers, nearest-neighbour distances and connectivity, were better predictors of occupancy when focal patch area was included in the model. Therefore, we expected a priori that studies that did not include the sampled wetland patch area in wetland amount would have a lower effect size compared to studies that did include the sampled wetland patch area.

### Effect Size Calculations

We selected the Pearson correlation coefficient *r* as our common estimate of effect size. When a study reported Spearman’s rank correlation coefficient (*ρ*), we converted *ρ* to *r* following [36]. If studies did not report a correlation coefficient, we transformed published test statistics as follows. For studies with continuous measures of population abundance, we extracted *r* values by taking the square root of reported R^2^ values from univariate linear regressions, and adding the sign of the slope. Note that we did not use partial R^2^ values [37]. When raw data were available, we calculated *r* for species with occupancy rates ≥0.7. Data sets with occupancy rates ≤0.7 did not meet normality assumptions of *r*; in this case we converted continuous data to occupancy data to determine an effect size. For studies that measured species occupancy or reported means and variances between two groups (e.g. mean wetland amount in occupied vs. unoccupied landscapes), we first calculated the standardized mean difference (ES_sm_) following [35]. We then converted each ES_sm_ to *r* following [38]. We transformed all correlation coefficients to Fisher’s *z*-scale (ES*_Zr_*) following [38].

The next step was to obtain accurate and comparable sample sizes across studies. Meta-analysis weights each study by its inverse variance, based on the assumption that studies with greater precision provide a more accurate estimate of the true effect. The variance of ES*_Zr_* is approximated as V*_Zr_*  = 1/(*n*-3), where *n* is the total sample size of the study [38]. This gives more weight to studies with larger sample sizes; however, this may overweight studies with pseudoreplication, a common problem in landscape ecology [39]. For example, within a given study area, studies that selected spatially independent wetlands in non-overlapping landscapes at a landscape size (i.e. scale) based on a species’ biology may have a lower apparent sample size than studies that sampled as many wetlands as possible without consideration of spatial independence. In the latter, the sample size would be inflated due to non-independence of sample points because almost identical (i.e. overlapping) landscapes were incorrectly used as multiple independent observations [39]. In addition, if neighbouring species sample points are closer together than the movement range of an individual, the same individual may be sampled more than once, again leading to violation of the assumption of independence. Therefore, we assessed the sample size of each study for pseudoreplication using process similar to that in Rytwinski and Fahrig [40], as follows. Our assessment was based on the assumption that each data point should represent a spatially independent sample. We assumed that an independent sample was equivalent to an independent individual in a spatially independent sampling location, such that it was unlikely the same individual was sampled at more than one wetland. We considered studies to have independent samples, and therefore accurate sample sizes, in two situations. First, studies that selected non-overlapping landscapes a priori based on the movement range of the species were assumed to be independent samples because it is unlikely that the same individual would be sampled in neighbouring sites. Second, in studies where each landscape represented the area around a sampled individual (e.g. a nest), the number of landscapes was already equivalent to the number of independent individuals. For all other studies, we adjusted sample size. When the distance between two sampling locations (e.g. wetlands) was less than the linear home range or territory size of the species, the two locations were counted as a single sample. For studies that compared population abundance in sampling locations to randomly selected locations where the species was known to be absent, and spatial information on these random locations was not available, the sample size was the number of spatially independent sampling locations plus one (to account for all random locations).

After determining the adjusted sample size (*n_adjusted_*) of each study, we calculated the inverse variance weight for each ES_Z*r*_ as *w = n_adjusted_* - 3 [41]. Studies with *w* <1 were excluded from the meta-analysis. Refer to Table S1 and Reference List S1 for studies included in the meta-analysis and associated country, species, effect sizes, adjusted sample sizes and study-design categories.

### Species Traits

Our main objective was to test if mobility and reproductive rate could explain variation in species responses to wetland habitat loss (Table 1). We collected data on these species traits from primary literature, theses and published species guides. We estimated mobility using two species traits, home range size and body size, which are both strongly correlated to dispersal distance across species of mammals and birds, independent of their migratory status [42]–[44]. Home range size for mammals was indexed as mean annual home range area (ha) [43]. For birds, the mean annual breeding territory size (ha) was used for species that forage primarily within their breeding territory [44]. For species that travel away from the nest site to forage (e.g. great blue heron, *Ardea herodias*), home range or foraging distance was reported in the literature rather than breeding territory. In these cases, mean annual home range area (ha) during the breeding season was used as a measure of territory size [44]. When annual home range estimates were not reported, we used the mean foraging distance as a diameter to calculate a circular home range area (ha). For studies conducted during the non-breeding season (e.g. migration, overwintering), we used territory size or foraging distances during the same season when available. For reptiles (turtles and snakes) and amphibians (anurans and salamanders), a cross-species relationship between either home range or body size and dispersal distance has not yet been demonstrated [45]. Therefore, we used adult annual movement ranges, which represented seasonal migration distances between breeding and summer (foraging) or overwintering sites [45]. This is often called a home range, but is conceptually different than a breeding home range or territory in mammals or birds. However, it is the only measure of space use that we could consistently obtain from the literature across all species of reptiles and amphibians. We assumed that species with larger adult annual movement ranges are more mobile in general and thus have larger dispersal distances than species with smaller adult annual movement ranges. Moreover, since body size is generally correlated with dispersal distance across several vertebrate taxa (including amphibians; [46]), it is also reasonable to assume that larger bodied reptile and amphibian species disperse greater distances than smaller bodied species ([47]; but see [48]). Therefore, for reptiles and amphibians, we used mean annual home range area (ha) as an index of mobility, preferably estimated using the minimum convex polygon method [49]. When minimum convex polygon estimates were not available, we estimated home range as a circular area (ha) with diameter equal to the mean seasonal migration distance [45]. All home range estimates were averaged between the sexes. Body size was measured as body length (mean body length across both sexes in centimeters); body mass was not used as it was unavailable for 37% of species (primarily amphibians), which represented half of the effect sizes included in the meta-analysis. We estimated reproductive rate as the mean litter or clutch size multiplied by the mean number of litters or clutches per year. Species traits were taken from sources as close to the study region of each study as possible. When trait data were available over multiple years, we used the mean value. Where studies reported a range in values, we used the mid-point of these ranges. For species complexes that were comprised of two species that could not be distinguished, we took the average of the traits of the species comprising the complex.

We classified effect sizes by taxonomic group at the class level: mammal, bird, reptile and amphibian, and by order within each taxonomic group. Since there were only two orders within amphibians, we also classified effect sizes by family for amphibians. Refer to Table S2 for life-history trait values and Reference List S2 for information sources.

### Meta-analysis

We first conducted a random-effects meta-analysis using the DerSimonian-Laird method to determine the summary weighted-mean effect size of the overall response of wetland species abundance to wetland loss at the landscape scale. Under the random-effects model, the weight assigned (w*) to each effect size is the inverse of the sum of two variance components w*  = 1/(*w*+*T* ^2^), where *w* (see above) is the unique sampling variance for each study (within-study error) and *T^2^* is the pooled variance of the true effects across all randomly selected studies (between-studies variance [38]. We also calculated the heterogeneity in true effects (*Q* statistic), which we compared against a chi-square distribution, to test whether the total variation in observed effect sizes (*Q*_T_) was significantly greater than that expected from sampling error (*Q*_E_). We then tested if moderator variables can explain true variation in the effect sizes (*Q*_M_), i.e. *Q*_T_ = *Q*_M_+*Q*_E_. All analyses were conducted using the ‘metafor’ package (version 1.7-0) in R 3.0 [50].

To test whether mobility, reproductive rate and/or study-level moderators explained a significant amount of heterogeneity in effect sizes, we performed a mixed-effects meta-regression using restricted maximum-likelihood estimation of heterogeneity. Since mobility and reproductive rate information was not available for all species, we removed all effect sizes with missing species trait data to have equal datasets. All species traits were log-transformed to meet test assumptions. We first tested if study design or taxonomic moderators influenced the effect sizes by performing univariate mixed-effects meta-analysis where, if study design or taxonomy explained significant heterogeneity in the effect sizes, we would then subset our data by that moderator variable to control for the effect of study design or taxonomy in analyses of the effects of mobility and reproductive rate. We then performed univariate mixed-effects meta-regression for home range, body length and reproductive rate.

We assessed publication bias by a rank correlation (Kendall’s tau) test of the relationship between ES_Z*r*_ and *n* in association with visual inspection of a scatterplot between these two variables [51].

## Results

Although we found more than 200 studies that examined the effect of wetland habitat amount in a landscape on population abundance of wetland vertebrates, only 90 studies met the inclusion criteria (Figure 1). These 90 studies generated 426 effect sizes across 220 species and 16 countries (Table S1). Studies were predominately from North America (60) and Europe (19), with remaining studies from Australia (5), Central and South America (3), Asia (2) and Africa (1). After removing effect sizes due to lack of information on mobility or reproductive rate, the total number of effect sizes was reduced to 334 across 137 species. The summary weighted-mean effect size from a random-effects meta-analysis across all taxa was 0.11 (95% CI: 0.089, 0.137; n  = 334), indicating an overall weak, positive effect of wetland amount in a landscape on wetland animal population abundance. The overall heterogeneity was *Q*  = 712.03 (p<0.0001), indicating highly significant variation in species responses to wetland amount. There was no strong evidence of publication bias as there was a weak relationship between effect size and sample size (Kendall's tau  = 0.03, p  = 0.36), and a scatterplot between these two variables showed effect sizes were symmetrically distributed around the summary effect and produced a funnel-shape with greater variation in studies at low sample sizes (Figure S1).

**Figure 2:**
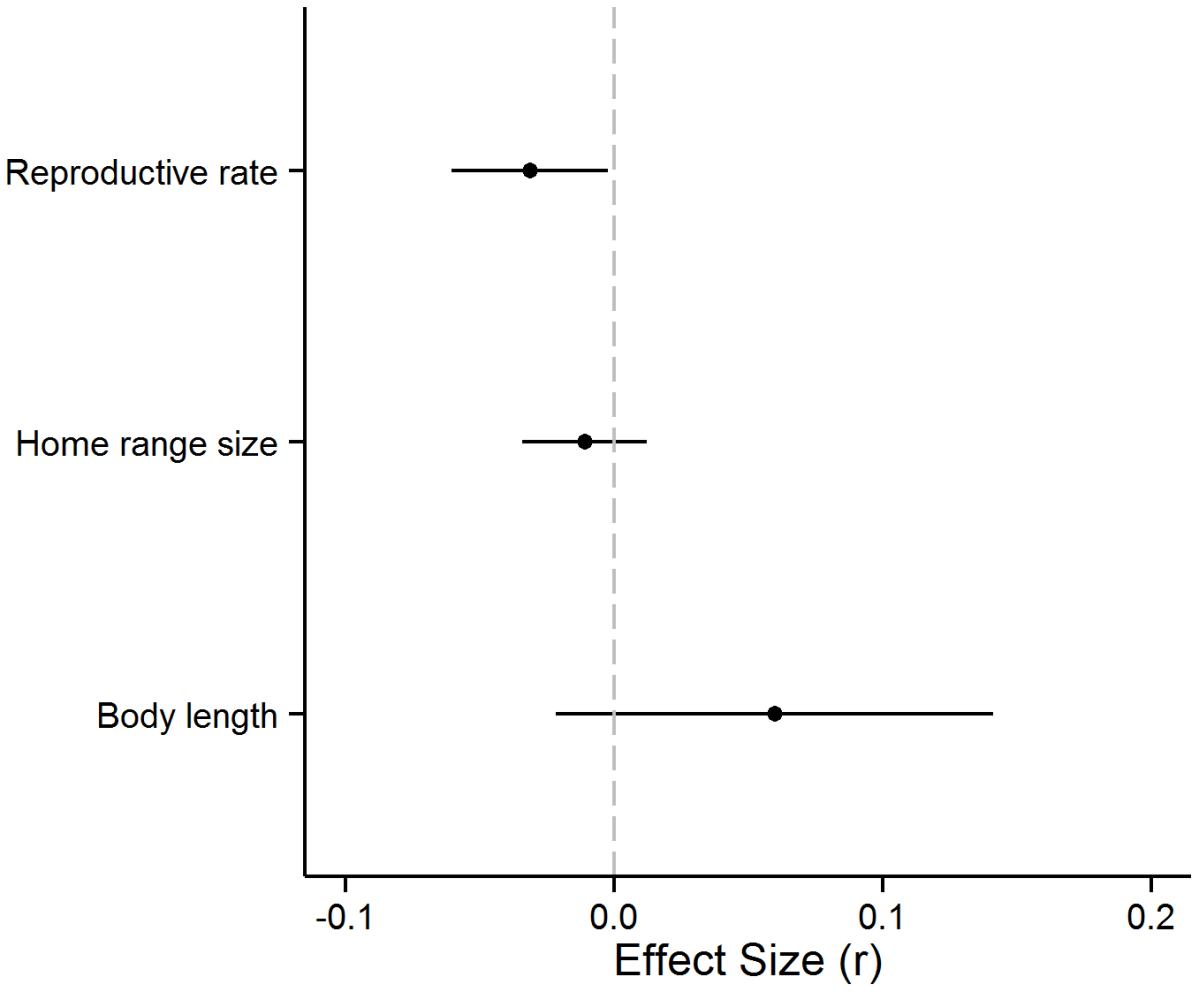
Effects of species traits on vertebrate response to wetland loss. Effects of reproductive rate and mobility (indexed as home range size and body length) on population response of wetland vertebrates to wetland habitat loss in a landscape, including mammals, birds, reptiles and amphibians (n  = 137 species). Points represent mean-weighted effect sizes (*z*-transformed correlation coefficients) from mixed-effects meta-regression and lines indicate 95% confidence intervals.

Mixed-effects meta-analysis across all taxa (n  = 334) showed that no study design moderator explained any significant heterogeneity in the effects (study type: *Q*_M_  = 0.65, p  = 0.42; sampling effort: *Q*_M_  = 4.34, p  = 0.11; sampled wetland area: *Q*_M_  = 1.26, p  = 0.26; Table S3). Therefore we did not control for study design in analyses of the influence of mobility and reproductive rate on species responses to habitat amount. Reproductive rate and body length explained significant heterogeneity in the effects of wetland amount on animal population abundance across all taxa (*Q*_M_  = 18.83, p<0.0001; *Q*_M_  = 16.02, p<0.0001, respectively; Table S3). Since the correlation between reproductive rate and body length was high (*r* = −0.72, p<0.0001, n  = 334), we performed a multiple meta-regression to test for independent effects of each moderator while accounting for the presence of the other (*Q*_M_  = 20.76, p<0.0001; Table S3). After controlling for the effect of body length, reproductive rate was negatively related to the effect of wetland amount on population abundance (ES_Z*r*_ = −0.03; 95% CI: −0.061, −0.002; p  = 0.03; Figure 2). In other words, species with lower reproductive rates were more sensitive to wetland amount in a landscape than species with higher reproductive rates. In contrast, after controlling for the effect of reproductive rate, the effect of body length was not significant (ES_Z*r*_  = 0.06; 95% CI: −0.025, 0.137; p  = 0.15; Figure 2).

Home range size did not explain any significant heterogeneity in effect sizes (*Q*_M_  = 0.54, p  = 0.46, n  = 334; Table S3). The correlation between home range and reproductive rate was *r* = −0.06, p  = 0.26, and between home range and body length was *r*  = 0.25, p<0.0001. Since home range did not explain significant heterogeneity and home range size information was missing for 92 of the total 426 effect sizes extracted across all taxa, we removed home range and re-analyzed the effect of body length and reproductive rate with a larger dataset (n  = 421). The multiple meta-regression containing body length and reproductive rate explained a significant amount of heterogeneity in the effect sizes of the larger dataset (*Q*_M_  = 25.38, p<0.0001). Consistent with the analysis with 334 effect sizes (above), reproductive rate was negatively related to the effect of wetland amount on population abundance (ES_Z*r*_ = −0.03; 95% CI: −0.061, −0.007; p  = 0.02) and body length had no significant effect (ES_Z*r*_  = 0.06; 95% CI: −0.021, 0.134; p  = 0.16). Refer to Table S4 for descriptive statistics of species traits across taxa.

**Figure 3:**
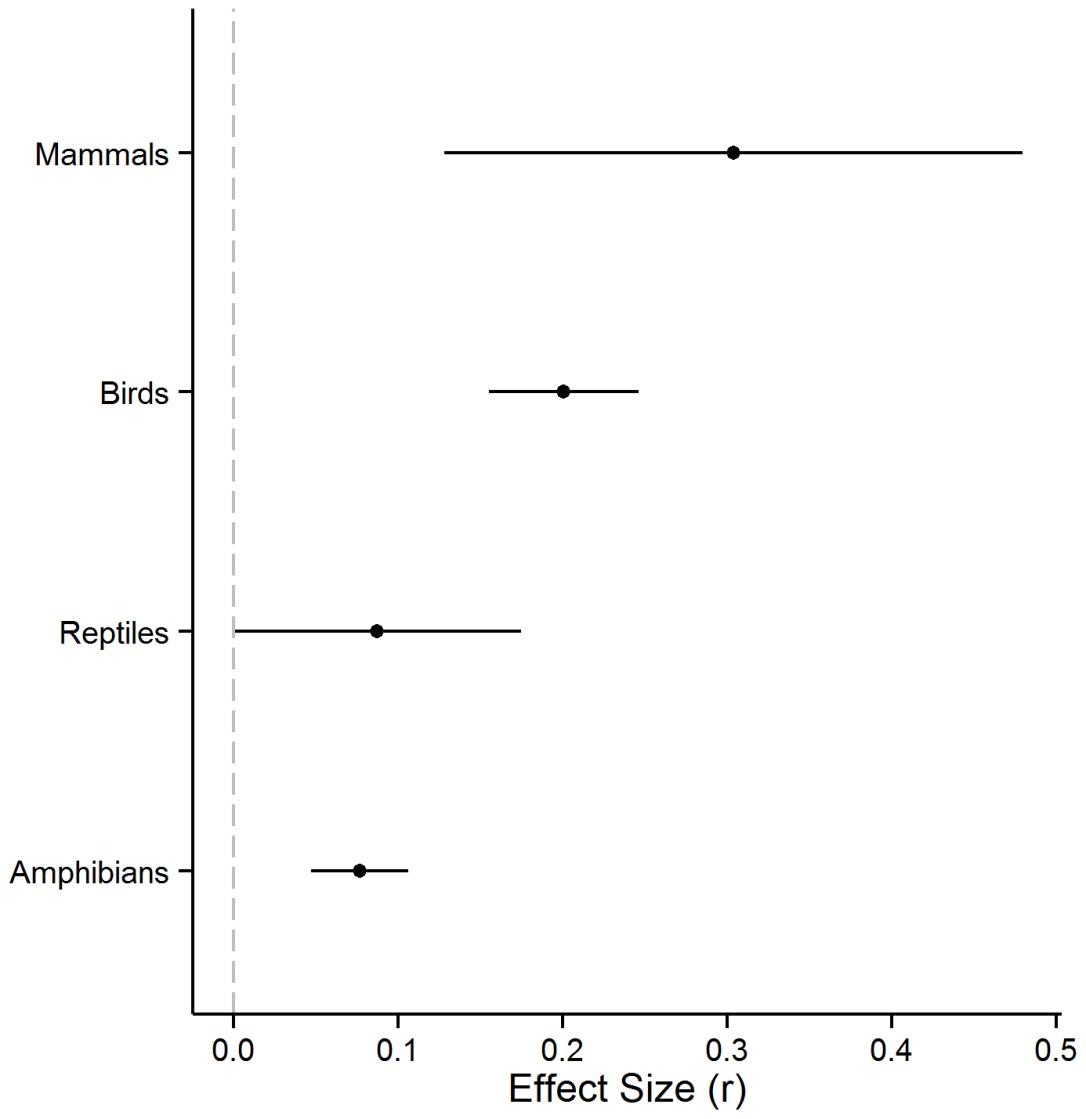
Wetland vertebrate response to wetland loss. Population-level responses of wetland vertebrate classes to wetland habitat loss in a landscape. Points represent mean-weighted effect sizes (*z*-transformed correlation coefficients) from mixed-effects meta-regression and lines indicate 95% confidence intervals.

The effect of the amount of wetland habitat in a landscape on animal abundance varied by taxonomic class (*Q*_M_  = 25.17, p<0.0001; Table S3). The weighted-mean effect size for mammals and birds was greater than that of reptiles and amphibians (Figure 3). The correlation between measures of mobility and reproductive rate differed across taxa, with negative correlations for mammals and birds, and positive correlations for reptiles and amphibians (Table 2). Since there was large heterogeneity of effect sizes within each taxon separately (birds: *Q*  = 168.96, p<0.0001, n  = 115; reptiles: *Q*  = 35.32, p  = 0.048, n  = 24; amphibians: *Q*  = 412.24, p<0.0001, n  = 189), we tested for effects of mobility and reproductive rate within taxa. Refer to Table S4 for descriptive statistics of species traits for each taxonomic group.

**Table 2:** Pearson correlation coefficients (above diagonals) and associated p-values (below diagonals and italicized) between species traits within each vertebrate class.

Taxa	# of effect sizes	Species Trait	log (home range^a^)	log (body length^b^)	log (reproductive rate^c^)
Mammals	6	log (home range)		−0.75	0.80
		log (body length)	*0.06*		−0.75
		log (reproductive rate)	*0.08*	*0.09*	
Birds	115	log (home range)		0.40	−0.55
		log (body length)	*<0.001*		−0.35
		log (reproductive rate)	*<0.001*	*<0.001*	
Reptiles	24	log (home range)		0.20	0.30
		log (body length)	*0.34*		0.55
		log (reproductive rate)	*0.15*	*0.01*	
Amphibians	189	log (home range)		-0.02	0.53
		log (body length)	*0.78*		0.07
		log (reproductive rate)	*<0.001*	*0.35*	

a home range (ha) = mean annual home range or seasonal migration distance across both sexes.

b body length (cm) = average total body length of the two sexes.

c reproductive rate = mean litter or clutch size multiplied by the mean number of litters or clutches per year.

For mammals, there were too few effect sizes (n  = 6) to meaningfully test for effects of species traits. For birds, differences in study design did not any explain any significant heterogeneity in effect sizes (study type: *Q*_M_  = 0.85, p  = 0.36, sampling effort: *Q*_M_  = 0.12, p  = 0.94, sampled wetland area: *Q*_M_  = 0.49, p  = 0.45; Table S3). Bird effect sizes did not vary significantly by Order (*Q*_M_  = 9.89, p  = 0.20; Table S3). Reproductive rate and body length explained significant heterogeneity in the effects of wetland amount on population abundance of birds (*Q*_M_  = 6.09, p  = 0.014; *Q*_M_  = 3.86, p  = 0.049, respectively; Table S3). A multiple meta-regression containing both body length and reproductive rate significantly explained heterogeneity (*Q*_M_  = 7.75, p  = 0.021). Reproductive rate was negatively related to the effect of wetland amount on bird population abundance and the effect of reproductive rate was marginally significant (ES_Z*r*_ = −0.25; 95% CI: −0.506, −0.001; p  = 0.050) while the effect of body length was non-significant (ES_Z*r*_  = 0.12; 95% CI: −0.056, 0.261; p  = 0.205). We were able to test for the effect of body mass, which was moderately correlated with body length in our bird dataset (r  = 0.47, p<0.001). Body mass did not explain heterogeneity in effect sizes for birds (*Q*_M_  = 0.315, p  = 0.58), nor did home range size (*Q*_M_  = 0.052, p  = 0.82).

For reptiles (turtles and snakes), response to wetland amount did not vary significantly by Order (*Q*_M_  = 0.66, p  = 0.42; Table S3). Population-level effects of wetland amount on reptiles varied by study type (*Q*_M_  = 6.10, p  = 0.01; Table S3); amount-based studies (area-based buffers) had a lower mean-weighted effect size (0.05) than configuration-based (isolation or connectivity) studies (0.31). However, we could not subset our data according to study type due to the small sample size (n  = 24). In any case, in the non-subsetted data, neither mobility nor reproductive rate explained any significant heterogeneity in the effect of wetland amount (Table S3). For amphibians (anurans and salamanders), population-level effects of wetland amount did not vary significantly by Order (*Q*_M_  = 0.183, p  = 0.668), but responses varying by Family was marginally significant (*Q*_M_  = 18.081, p  = 0.054; Table S3). Therefore, we subsetted our data by Family and tested for effects of mobility and reproductive rate within the largest subset, which was the Family Ranidae (n  = 62). Within the Ranidae subset, no study design moderator or species trait explained significant variation in the effect of wetland amount (Table S3). In the non-subsetted data, no study design moderator or species trait explained significant variation in the effect of wetland amount (Table S3).

## Discussion

Contrary to the widely-held assumption in metapopulation and landscape ecology, our results suggest that dispersal ability is not a useful predictor of species sensitivity to habitat loss, at least for wetland vertebrates. When analyzed across all taxa, we found no evidence to support the prediction that animals with greater mobility were less sensitive to wetland habitat loss than species with lower mobility, when measured as home range size or body length. Analyses within each taxon also showed no effect of home range size or body length on species responses to wetland loss, as well as no effect of body mass for bird responses. Bowne and Bowers [52] point out that despite the putative importance of mobility, there is very little evidence to validate the relationship between movement and population persistence. Consistent with our results, studies that compared the relative influence of mobility to other species traits found that home range or movement distances were weakly related to species responses to habitat loss [5]–[6], [20]. Moreover, studies that find strong effects of mobility are generally based on univariate models using indirect indices of mobility [53]. We found that body length explained heterogeneity in species responses to wetland loss in univariate models across all taxa and within birds, but the effect of body length was no longer significant when reproductive rate was controlled for. Consistent with our results, there is generally little empirical support for a relationship between body size and sensitivity to habitat loss in vertebrates [5]–[8], [33], [54].

A possible reason for the apparent lack of influence of mobility on species responses to habitat loss is that the effect of mobility varies. While several empirical studies have shown that more mobile species are less sensitive to habitat loss [53], [55], other studies have found the opposite, that greater mobility decreases tolerance to habitat loss [14]–[16]. If our meta-analysis included some species with mobility that positively influenced response to wetland amount and other species with mobility that negatively influenced response to wetland amount, it is possible that the two response types canceled each other out and resulted in no overall effect of mobility. Alternatively, no effect of mobility could have also resulted from a non-linear relationship with species responses to habitat loss. Thomas [56] found that butterflies with intermediate mobility were more vulnerable to habitat loss than butterflies with either low or high mobility. However, a scatterplot of species mobility and species responses to wetland loss was highly scattered in our dataset, indicating a very weak relationship rather than a non-linear one (Figure S2).

A second possible reason for the apparent lack of influence of mobility on species responses to habitat loss could be that mobility varies widely with landscape structure, such that the relative rankings of species’ mobility changes as the landscape changes [57]. For example, translocation experiments of two forest bird species in three different landscape types (forested, timber harvested, agricultural) showed that the relative ability of each species to move in a landscape changed depending on landscape context [58]. Ovenbirds (*Seiurus aurocapilla*; forest specialist) had greater return rates than white-throated sparrows (*Zonotrichia albicollis*; forest generalist) in forested landscapes, whereas the opposite was found in harvested or agricultural landscapes. Several studies have shown that species’ movement distances vary depending on landscape context [59]–[61] and that movement is a product of both species traits and landscape structure [62]. For example, home range sizes of northern saw-whet owls (*Aegolius acadicus*; [63]) and elk (*Cervus elaphus*; [64]) increased by an order of magnitude as the amount of forest cover increased in a landscape. Similar to the results of our meta-analysis, Ferraz et al. [65] found no effect of dispersal ability on patch occupancy responses of 55 tropical birds to forest patch isolation. They suggest as a possible explanation that species dispersal abilities change in disturbed landscapes, such that mobility estimated in continuous habitat is not useful to predict occupancy parameters in human-dominated landscapes. Fahrig [57] suggested that species mobility cannot be estimated independently of landscape structure and that to test dispersal ability the landscape context should match the location where movement data were collected. We were unable to test mobility using home range estimates that matched the location of each study included in our meta-analysis since home range estimates are generally very limited.

Lastly, it is possible that we did not find an effect of mobility because of high uncertainty in dispersal estimates for vertebrates [52]. We attempted to reduce this problem by using two measures of mobility, home range area and body size, that are known to be highly correlated with dispersal distance [42]–[44], [46]. However, the error associated with such estimates is still likely higher than the error associated with estimates of reproductive rate. For example, home range estimates for some species included in the meta-analysis varied by two orders of magnitude both within and between populations (e.g. [66]–[69]). If true, this means we were *a priori* more likely to find effects of reproductive rate than mobility on species sensitivity to habitat loss. Furthermore, although home range size is strongly correlated with dispersal distance in mammals and birds, we assumed this relationship for reptiles and amphibians (see Methods). If the relationship is weaker for reptiles and amphibians than for mammals and birds, body size is a more uncertain measure of mobility for reptiles and amphibians.

Body size is commonly used to index sensitivity to habitat loss in animal taxa [5]–[8], [54]. However, the exact inference one can make from a cross-species effect of body size ambiguous because body size is simultaneously correlated to several life-history attributes (reviewed in [2]). For example, in addition to its positive correlation with dispersal, body size is positively correlated with area requirements and trophic level, and these relationships occur indirectly through a negative correlation with natural abundances and population fluctuations [2]. We had expected that body size would be the best predictor of wetland vertebrate response to habitat loss because it is an indirect measure of several life-history mechanisms. However, we found that, in wetland vertebrates, the effect of body size was no longer significant once we had controlled for the effect of reproductive rate.

Our results provide support for the hypothesis that animals with lower reproductive rates are more negatively affected by habitat loss than are animals with higher reproductive rates. When analyzed across all taxa, reproductive rate was the main explanatory variable for population-level responses to habitat loss, and the effect remained strong when controlling for body size (Figure 2; Table S3). This suggests that reproductive rate affects species response to habitat loss, independent of its correlation with body size. Other studies have also found a greater effect of reproductive rate than movement-related traits. Simulation studies found that reproductive rate has a much larger effect on the amount of habitat required for population persistence than the per capita rate of emigration [17] and dispersal ability [18]. An empirical test of the relative effects of reproductive rate and mobility at the landscape scale [20] found a strong negative association between reproductive rate and minimum habitat required for a group of dead wood boring beetles, whereas the effect of emigration rate was no longer significant once reproductive rate was controlled for. The only other empirical test of the effect of reproductive rate at the landscape scale that we have found, Vance et al. [19], reported a strong negative cross-species relationship between reproductive rate and the amount of forest in a landscape for forest birds. In line with our results, a recent meta-analysis of road and/or traffic effects across the same vertebrate taxa found that reproductive rate explained a larger amount of variation in population responses of mammals and amphibians to roads than mobility (indexed as home range size) and body size [40]. Both types of landscape change – increased road density or habitat loss – both result in the loss of individuals. Therefore, the mechanism linking the landscape change to reproductive rate is the same: higher reproductive rates compensate for increased mortality and reduce local extinction risk [70].

In contrast, several patch scale studies have found no effect of reproductive rate on species response to patch size and isolation. In a large meta-analysis of patch occupancy of 785 animal species across several taxa, Prugh et al. [4] found no effect of fecundity on species responses to patch area and isolation. Similarly, no effect of reproductive rate was found on patch occupancy rates of 25 mid- and large-sized Neotropical mammals in sites within fragmented compared to continuous forest landscapes [5] or on the number of islands occupied by five lizard species [71]. However, consistent with our results, a meta-analysis of patch area effects on butterfly species richness showed a negative effect of reproductive rate [21]. Patch scale studies may not find an effect of reproductive rate if patch size is not correlated to habitat amount in the landscape. A greater amount of habitat in a landscape represents a greater number of potential colonists available to a patch via the mass effect that is the net flow of individuals from high abundance areas to low abundance areas [72]. For given amount of habitat in a landscape, species with higher reproductive rates will on average produce more colonists than species with lower reproductive rates. This high influx of individuals (immigration) from surrounding habitat would increase local population size with increasing habitat in the landscape [73]. This mechanism would only occur in patch-scale studies if the amount of habitat in the landscape is positively correlated with patch size [73].

On the other hand, the relationship between reproductive rate and species response to habitat loss could occur through a correlation between reproductive rate and another unmeasured variable. For example, reproductive rate was found to be highly correlated with habitat and diet breadth in mammals [5], and niche breadth (composite of habitat and diet) was found to have a greater influence on patch occupancy rates of mammals and amphibians than body size [54]. Similarly, feeding guild (index of diet) was most strongly related to fragmentation sensitivity in a review of Neotropical vertebrate (including mammals, birds, reptiles and amphibians) responses at the patch-scale compared to body size [7]. However, in both Swihart et al. [54] and Vetter et al. [7], reproductive rate was not tested. Interestingly, a meta-analysis by Newbold et al. [8] found that both generation length (surrogate for annual reproductive rate), habitat and diet breadth were in the top AIC models (ΔAIC <2) explaining population-level responses of pan-tropical birds to surrounding landuse intensity. This suggests that the mechanism behind the effect of reproductive rate on population response to habitat loss, i.e. greater reproductive output and potential colonists, may act independently of any correlation with niche breadth, at least for birds. We are unable to test for an effect of niche breadth due to the lack of detailed habitat and diet information for most species included in our meta-analysis.

We found that wetland mammals and birds were more sensitive to wetland amount in a landscape than were wetland reptiles and amphibians (Figure 3). This difference was also found by Prugh et al. [4] in a meta-analysis on patch area effects. These results may reflect the fact that many wetland reptile and amphibian species require more than one habitat type in a landscape to complete their life-cycle (e.g. foraging, nesting, hibernation) to sustain populations (i.e. landscape complementation) [74]. In fact, the amount of forest in a landscape was found to be more important than wetland amount or connectivity for the occurrence of wetland reptiles [75], [76] and many species of wetland-breeding amphibians have a strong, positive association with the amount of forest in a landscape [77]–[79]. If access to or quantity of complementary habitat is limited, local population sizes will be low despite high wetland amount in a landscape. In fact, reptiles and amphibians were found to be more susceptible to negative effects of roads than mammals and birds [40]. Taken together, these results suggest that wetland reptiles and amphibians are less limited by the amount of wetland habitat in a landscape than are wetland mammals and birds. Other factors, such as landscape complementation or road mortality, may have stronger effects than wetland loss on abundance and distribution of wetland reptiles and amphibians.

We present a comprehensive, worldwide review of wetland vertebrate responses to wetland habitat loss at a landscape scale (Checklist S1); however some limitations need to be considered. First, although we included study level moderators to control for potential bias at the study level, effect sizes obtained are likely influenced by study area attributes and the scale selected for analysis. Prugh et al. [4] found that patch area and isolation effects varied depending on the predominant land cover in a study area. While we did not find an effect of study area type (natural, agricultural, rural or urban) on responses to wetland amount (*Q*_M_  = 4.63, p  = 0.20; Table S3), we could not assess whether other landscape variables (e.g. roads) confounded the effect of wetland loss. Moreover, if studies that conduced analysis at multiple scales are more likely to find the scale of effect for a given species [27], then studies that selected only one scale of analysis may systematically have lower effect sizes. However, we did not find an effect of the number of scales selected by a study on species response to wetland amount (*Q*_M_  = 1.48, p  = 0.22; Table S3). At the review-level, although we attempted to include unpublished studies such as theses (4 studies) and government reports (2 studies) in our review, published literature was the primary data source in our meta-analysis, representing 93% of studies. While we did not find evidence of publication bias, our review may be biased towards species from geographical areas with high publication rates (North America, Europe). Lastly, since the majority of studies were conducted on birds and amphibians, our mammal and reptile results are less solid.

## Conclusions

Our synthesis shows that wetland habitat loss at the landscape scale has an overall negative effect on the population abundance of wetland vertebrates across many taxonomic groups and landscape contexts worldwide. Our results support the hypothesis that species with lower reproductive rates are more negatively affected by landscape-scale habitat loss than are species with higher reproductive rates. Surprisingly, we found no evidence that mobility influences species response to habitat loss. This implies that immigration and colonization rate is more strongly related to reproduction, which determines the total number of potential colonists, than it is to a species’ intrinsic mobility. From a conservation management perspective, our results suggest that priority should be placed on species with low reproductive rates. Also, our results suggest that conservation plans for declining wetland species should focus on actions aimed at increasing reproductive output, such as providing artificial nesting substrates or managing local wetland variables that increase reproductive success of target species (e.g. hydroperiod [80]; vegetation structure [81]–[82]).

## Supporting Information

Figure S1 **Relationship between** ***Z*** **-transformed correlation coefficients (ES_Z_**_***r***_ **) and sample size (n) to assess publication bias.** Dashed line is the summary mean-weighted effect size from random-effect meta-analysis across 426 effect sizes from 90 studies. There was no strong evidence of publication bias since effect sizes were symmetrically distributed around the summary effect and produced a funnel-shape with greater variation in studies at low sample sizes.
(TIF)

Figure S2 **Scatterplot of log home range size (ha) and response to wetland habitat loss in a landscape (ES_Z_**_***r***_ **) for all species included in the meta-analysis, including mammals, birds, reptiles and amphibians (n  = 334).**
(JPEG)

Table S1 **Studies included in the meta-analysis and associated species, effect sizes (ES** ***r*** **), adjusted sample sizes (n), and study design categories (study type, sampling effort, and patch area).**
(DOCX)

Table S2 **Species traits and reference information for the 220 species used in the meta-analysis.**
(DOCX)

Table S3 **Results from univariate mixed-effects meta-analytic models testing the effect of mobility (home range size and body length) and reproductive rate on species responses to wetland habitat loss at the population-level across and within taxonomic groups.**
(DOCX)

Table S4 **Descriptive statistics of species traits (untransformed values) across all wetland vertebrates and within taxonomic groups used in the meta-analysis.**
(DOCX)

Checkist S1 **PRISMA Checklist.**
(DOCX)

Reference List S1 **Studies included in the meta-analysis.**
(DOCX)

Reference List S2 **Species trait references for species used in the meta-analysis.**
(DOCX)
