# Supplementary figures and images for: Low Reproductive Rate Predicts Species Sensitivity to Habitat Loss: A Meta-Analysis of Wetland Vertebrates
Source: PLoS One. 2014 Mar 20;9(3):e90926. doi: 10.1371/journal.pone.0090926 (PMC3961235; doi:10.1371/journal.pone.0090926)

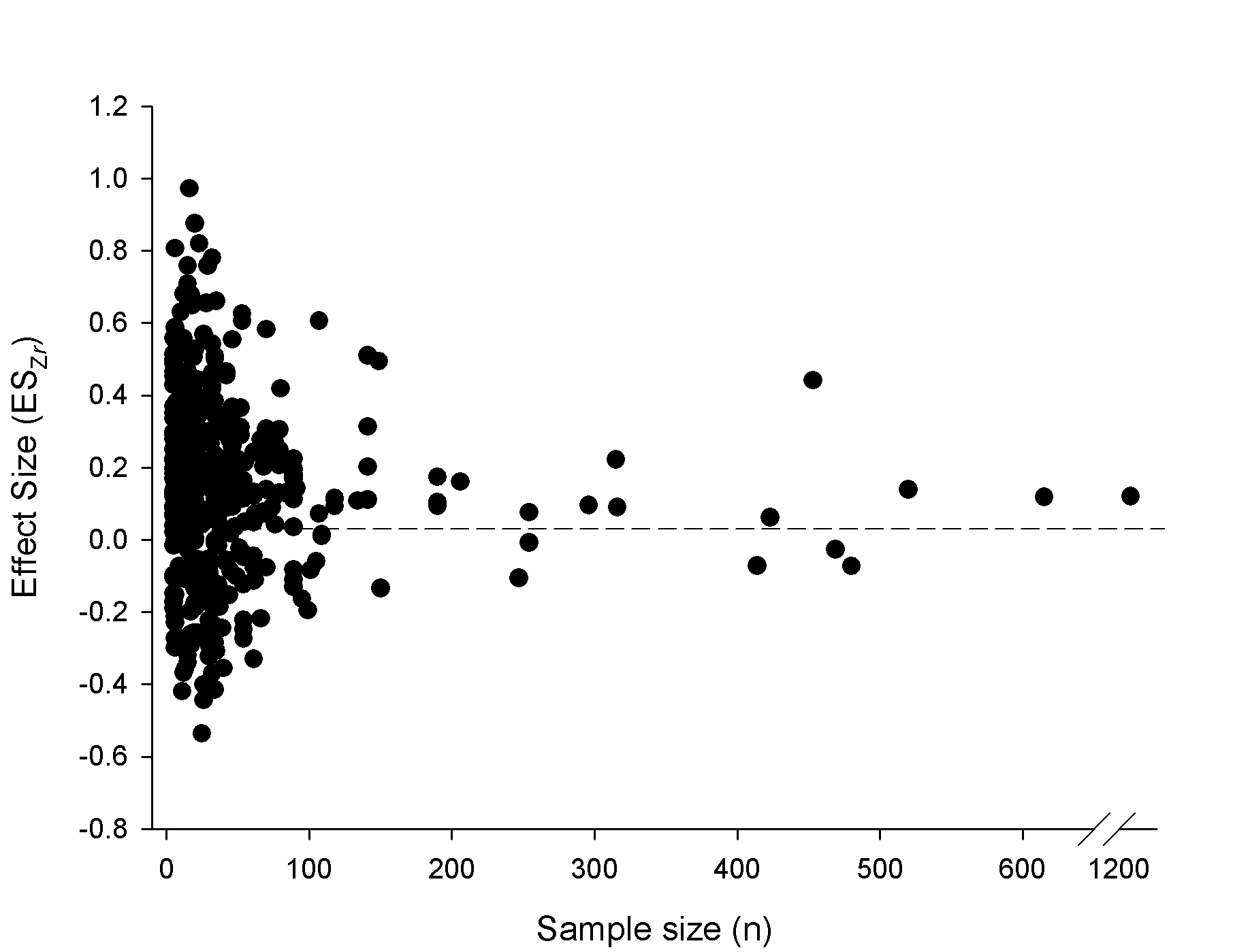

Supplement: Figure S1 — Relationship between Z -transformed correlation coefficients (ESZ r ) and sample size (n) to assess publication bias. Dashed line is the summary mean-weighted effect size from random-effect meta-analysis across 426 effect sizes from 90 studies. There was no strong evidence of publication bias since effect sizes were symmetrically distributed around the summary effect and produced a funnel-shape with greater variation in studies at low sample sizes. (TIF) [file pone.0090926.s001.tif]

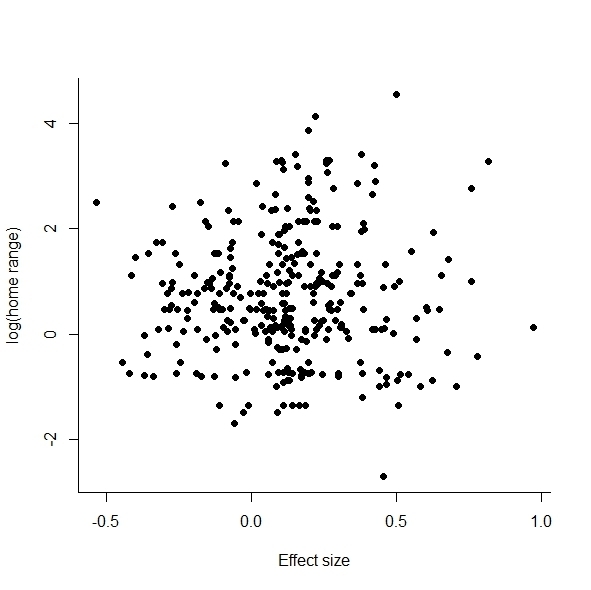

Supplement: Figure S2 — Scatterplot of log home range size (ha) and response to wetland habitat loss in a landscape (ESZ r ) for all species included in the meta-analysis, including mammals, birds, reptiles and amphibians (n = 334). (JPEG) [file pone.0090926.s002.jpeg]
