# Supplementary material for: Low Reproductive Rate Predicts Species Sensitivity to Habitat Loss: A Meta-Analysis of Wetland Vertebrates
Source: PLoS One. 2014 Mar 20;9(3):e90926. doi: 10.1371/journal.pone.0090926 (PMC3961235; doi:10.1371/journal.pone.0090926)
Supplement: Table S1 — Studies included in the meta-analysis and associated species, effect sizes (ES r ), adjusted sample sizes (n), and study design categories (study type, sampling effort, and patch area). (DOCX) [file pone.0090926.s003.docx]

Table S1: Studies included in the meta-analysis and associated species, effect sizes (ES*r*), adjusted sample sizes (n), and study design categories (study type, sampling effort, and patch area).

| Study* | Country | Taxa^+^ | Species | ES*r* | n | Study Type | Sampling Effort | Patch Area |
| --- | --- | --- | --- | --- | --- | --- | --- | --- |
| 1 | Canada | m | *Castor canadensis* | 0.28 | 24 | Amount | Independent | no |
| 2 | USA | m | *Oryzomys palustris* | 0.19 | 35 | Amount | Dependent | yes |
| 3 | USA | m | *Microtus pennsylvanicus* | 0.06 | 15 | Amount | Dependent | yes |
| 3 | USA | m | *Sorex fumeus* | 0.31 | 15 | Amount | Dependent | yes |
| 3 | USA | m | *Synaptomys cooperi* | 0.11 | 15 | Amount | Dependent | yes |
| 4 | USA | m | *Sylvilagus palustris hefneri* | 0.3 | 39 | Amount | Dependent | yes |
| 5 | USA | m | *Neofiber alleni* | 0.42 | 453 | Configuration | Dependent | no |
| 6 | Japan | b | *Egretta intermedia* | 0.14 | 32 | Amount | Independent | yes |
| 6 | Japan | b | *Nycticorax nycticorax* | 0.43 | 32 | Amount | Independent | yes |
| 6 | Japan | b | *Ardea cinerea* | 0.4 | 32 | Amount | Independent | yes |
| 7 | USA | b | *Botaurus lentiginosus* | 0.12 | 50 | Amount | Dependent | yes |
| 7 | USA | b | *Ixobrychus exilis* | 0.22 | 50 | Amount | Dependent | yes |
| 8 | USA | b | *Botaurus lentiginosus* | 0.14 | 83 | Amount | Independent | yes |
| 8 | USA | b | *Podilymbus podiceps* | 0.1 | 190 | Amount | Independent | yes |
| 8 | USA | b | *Fulica americana* | 0.17 | 190 | Amount | Independent | yes |
| 8 | USA | b | *Porzana carolina* | 0.09 | 190 | Amount | Independent | yes |
| 8 | USA | b | *Ixobrychus exilis* | 0.21 | 79 | Amount | Independent | yes |
| 9 | Spain | b | *Circus aeruginosus* | 0.15 | 35 | Configuration | Unknown | no |
| 10 | Netherlands | b | *Acrocephalus scirpaceus* | 0.09 | 316 | Amount | Dependent | no |
| 11 | USA | b | *Ardea herodia* | 0.64 | 29 | Amount | Dependent | no |
| 12 | USA | b | *Ardea herodia* | 0.28 | 29 | Amount | Independent | no |
| 13 | USA | b | *Butorides virescens* | -0.17 | 5 | Configuration | Dependent | no |
| 13 | USA | b | *Gallinago delicata* | 0.12 | 5 | Configuration | Dependent | no |
| 13 | USA | b | *Circus cyaneus* | 0.04 | 5 | Configuration | Dependent | no |
| 13 | USA | b | *Botaurus lentiginosus* | -0.15 | 5 | Configuration | Dependent | no |
| 13 | USA | b | *Podilymbus podiceps* | -0.1 | 5 | Configuration | Dependent | no |
| 13 | USA | b | *Rallus limicola* | -0.19 | 5 | Configuration | Dependent | no |
| 13 | USA | b | *Porzana carolina* | -0.02 | 5 | Configuration | Dependent | no |
| 14 | UK | b | *Botaurus stellaris* | 0.15 | 44 | Amount | Independent | yes |
| 15 | USA | b | *Cistothorus palustris* | 0.09 | 11 | Amount | Independent | yes |
| 15 | USA | b | *Agelaius phoeniceus* | 0.17 | 11 | Amount | Dependent | yes |
| 15 | USA | b | *Cistothorus platensis* | -0.4 | 11 | Amount | Independent | yes |
| 16 | Brazil | b | *Plegadis chihi* | 0.4 | 9 | Amount | Dependent | yes |
| 16 | Brazil | b | *Platalea ajaja* | 0.22 | 5 | Amount | Dependent | yes |
| 16 | Brazil | b | *Theristicus caerulescens* | 0.47 | 5 | Amount | Dependent | yes |
| 16 | Brazil | b | *Nycticorax nycticorax* | 0.06 | 32 | Amount | Dependent | yes |
| 16 | Brazil | b | *Egretta thula* | 0.25 | 9 | Amount | Dependent | yes |
| 16 | Brazil | b | *Ardea cocoi* | 0.53 | 6 | Amount | Dependent | yes |
| 16 | Brazil | b | *Mycteria americana* | 0.19 | 5 | Amount | Dependent | yes |
| 16 | Brazil | b | *Syrigma sibilatrix* | 0.07 | 5 | Amount | Dependent | yes |
| 16 | Brazil | b | *Ciconia maguari* | 0.42 | 5 | Amount | Dependent | yes |
| 16 | Brazil | b | *Phimosus infuscatus* | 0.51 | 5 | Amount | Dependent | yes |
| 16 | Brazil | b | *Butorides striata* | 0.25 | 5 | Amount | Dependent | yes |
| 16 | Brazil | b | *Circus buffoni* | 0.13 | 5 | Amount | Dependent | yes |
| 16 | Brazil | b | *Ardea alba* | 0.08 | 6 | Amount | Dependent | yes |
| 16 | Brazil | b | *Chauna torquata* | 0.17 | 5 | Amount | Dependent | yes |
| 16 | Brazil | b | *Gallinago paraguaiae* | 0.32 | 5 | Amount | Dependent | yes |
| 16 | Brazil | b | *Himantopus himantopus* | 0.13 | 42 | Amount | Dependent | yes |
| 16 | Brazil | b | *Pardirallus sanguinolentus* | 0.18 | 5 | Amount | Dependent | yes |
| 16 | Brazil | b | *Gallinula melanops* | -0.1 | 5 | Amount | Dependent | yes |
| 16 | Brazil | b | *Rollandia rolland* | 0.43 | 42 | Amount | Dependent | yes |
| 16 | Brazil | b | *Jacana jacana* | 0.43 | 42 | Amount | Dependent | yes |
| 16 | Brazil | b | *Anas flavirostris* | 0.28 | 5 | Amount | Dependent | yes |
| 16 | Brazil | b | *Amazonetta brasiliensis* | 0.43 | 5 | Amount | Dependent | yes |
| 16 | Brazil | b | *Anas versicolor* | 0.22 | 5 | Amount | Dependent | yes |
| 16 | Brazil | b | *Dendrocygna viduata* | 0.27 | 5 | Amount | Dependent | yes |
| 16 | Brazil | b | *Callonetta leucophrys* | 0.12 | 5 | Amount | Dependent | yes |
| 16 | Brazil | b | *Rostrhamus sociabilis* | 0.26 | 9 | Amount | Dependent | yes |
| 16 | Brazil | b | *Dendrocygna bicolor* | 0.22 | 5 | Amount | Dependent | yes |
| 16 | Brazil | b | *Netta peposaca* | 0.35 | 5 | Amount | Dependent | yes |
| 16 | Brazil | b | *Aramus guarauna* | 0.05 | 35 | Amount | Dependent | yes |
| 16 | Brazil | b | *Fulica leucoptera* | 0.02 | 5 | Amount | Dependent | yes |
| 16 | Brazil | b | *Gallinula galeata* | 0.58 | 35 | Amount | Dependent | yes |
| 16 | Brazil | b | *Aramides ypecaha* | 0.09 | 5 | Amount | Dependent | yes |
| 16 | Brazil | b | *Podilymbus podiceps* | 0.06 | 35 | Amount | Dependent | yes |
| 17 | Canada | b | *Botaurus lentiginosus* | 0.27 | 141 | Amount | Dependent | yes |
| 17 | Canada | b | *Ixobrychus exilis* | 0.47 | 141 | Amount | Independent | yes |
| 17 | Canada | b | *Podilymbus podiceps* | 0.3 | 141 | Amount | Independent | yes |
| 17 | Canada | b | *Rallus limicola* | 0.2 | 141 | Amount | Independent | yes |
| 17 | Canada | b | *Porzana carolina* | 0.11 | 141 | Amount | Independent | yes |
| 18 | USA | b | *Ardea alba* | 0.67 | 23 | Amount | Independent | yes |
| 18 | USA | b | *Ardea herodias* | 0.39 | 32 | Amount | Independent | yes |
| 19 | Uganda | b | *Serinus koliensis* | 0.35 | 17 | Configuration | Dependent | no |
| 19 | Uganda | b | *Laniarius mufumbiri* | 0.01 | 17 | Configuration | Dependent | no |
| 19 | Uganda | b | *Chloropeta gracilirostris* | 0.01 | 17 | Configuration | Dependent | no |
| 19 | Uganda | b | *Acrocephalus rufescens* | 0.36 | 17 | Configuration | Dependent | no |
| 19 | Uganda | b | *Bradypterus carpalis* | 0.28 | 17 | Configuration | Dependent | no |
| 19 | Uganda | b | *Cisticola carruthersi* | 0.18 | 17 | Configuration | Dependent | no |
| 20 | USA | b | *Circus cyaneus* | -0.26 | 17 | Amount | Independent | yes |
| 21 | Spain | b | *Circus aeruginosus* | 0.36 | 7 | Configuration | Dependent | no |
| 21 | Spain | b | *Acrocephalus arundinaceus* | -0.34 | 13 | Configuration | Dependent | no |
| 21 | Spain | b | *Remiz pendulinus* | 0.36 | 14 | Configuration | Dependent | no |
| 21 | Spain | b | *Cettia cetti* | 0.43 | 11 | Configuration | Dependent | no |
| 21 | Spain | b | *Acrocephalus scirpaceus* | -0.03 | 15 | Configuration | Dependent | no |
| 21 | Spain | b | *Rallus aquaticus* | -0.33 | 15 | Configuration | Dependent | no |
| 22 | USA | b | *Limosa fedoa* | 0.12 | 118 | Amount | Dependent | yes |
| 22 | USA | b | *Phalaropus tricolor* | 0.09 | 118 | Amount | Dependent | yes |
| 22 | USA | b | *Recurvirostra americana* | 0.11 | 118 | Amount | Dependent | yes |
| 22 | USA | b | *Tringa semipalmata* | 0.11 | 118 | Amount | Dependent | yes |
| 23 | USA | b | *Ixobrychus exilis* | 0.07 | 5 | Amount | Dependent | no |
| 23 | USA | b | *Porphyrio martinica* | 0.45 | 5 | Amount | Dependent | no |
| 23 | USA | b | *Gallinula galeata* | 0.4 | 5 | Amount | Dependent | no |
| 23 | USA | b | *Rallus elegans* | 0.29 | 5 | Amount | Dependent | no |
| 23 | USA | b | *Dendrocygna bicolor* | 0.09 | 5 | Amount | Dependent | no |
| 24 | Canada | b | *Ixobrychus exilis* | 0.24 | 70 | Amount | Dependent | yes |
| 24 | Canada | b | *Melospiza georgiana* | 0.24 | 70 | Amount | Dependent | yes |
| 24 | Canada | b | *Setophaga petechia* | 0.07 | 70 | Amount | Dependent | yes |
| 24 | Canada | b | *Cistothorus palustris* | 0.52 | 70 | Amount | Dependent | yes |
| 24 | Canada | b | *Podilymbus podiceps* | 0.3 | 70 | Amount | Dependent | yes |
| 24 | Canada | b | *Rallus limicola* | 0.29 | 70 | Amount | Dependent | yes |
| 24 | Canada | b | *Geothlypis trichas* | 0.14 | 70 | Amount | Dependent | yes |
| 25 | USA | b | *Botaurus lentiginosus* | 0.29 | 10 | Amount | Independent | yes |
| 25 | USA | b | *Ixobrychus exilis* | 0.03 | 15 | Amount | Independent | yes |
| 25 | USA | b | *Porzana carolina* | 0.18 | 16 | Amount | Independent | yes |
| 25 | USA | b | *Rallus limicola* | 0.14 | 16 | Amount | Independent | yes |
| 25 | USA | b | *Podilymbus podiceps* | 0.75 | 16 | Amount | Independent | yes |
| 26 | Spain | b | *Himantopus himantopus* | 0.24 | 21 | Configuration | Dependent | no |
| 26 | Spain | b | *Tachybaptus ruficollis* | 0.24 | 21 | Configuration | Dependent | no |
| 27 | Thailand | b | *Limnodromus semipalmatus* | 0.38 | 20 | Amount | Independent | yes |
| 27 | Thailand | b | *Charadrius mongolus/C. leschenaultii* | 0.29 | 20 | Amount | Independent | yes |
| 27 | Thailand | b | *Calidris canutus* | 0.19 | 20 | Amount | Independent | yes |
| 27 | Thailand | b | *Recurvirostra avosetta* | 0.19 | 20 | Amount | Independent | yes |
| 27 | Thailand | b | *Calidris ferruginea* | 0.16 | 20 | Amount | Independent | yes |
| 27 | Thailand | b | *Actitis hypoleucos* | 0.03 | 20 | Amount | Independent | yes |
| 27 | Thailand | b | *Arenaria interpres* | 0.19 | 20 | Amount | Independent | yes |
| 27 | Thailand | b | *Calidris ruficollis* | 0.38 | 20 | Amount | Independent | yes |
| 27 | Thailand | b | *Calidris subminuta* | 0.7 | 20 | Amount | Independent | yes |
| 27 | Thailand | b | *Calidris tenuirostris* | 0.11 | 20 | Amount | Independent | yes |
| 27 | Thailand | b | *Charadrius dubius* | 0.36 | 20 | Amount | Independent | yes |
| 27 | Thailand | b | *Eurynorhynchus pygmeus* | 0.16 | 20 | Amount | Independent | yes |
| 27 | Thailand | b | *Gallinago gallinago* | 0 | 20 | Amount | Independent | yes |
| 27 | Thailand | b | *Limicola falcinellus* | 0.22 | 20 | Amount | Independent | yes |
| 27 | Thailand | b | *Limosa lapponica* | 0.16 | 20 | Amount | Independent | yes |
| 27 | Thailand | b | *Limosa limosa* | -0.08 | 20 | Amount | Independent | yes |
| 27 | Thailand | b | *Numenius arquata* | 0.1 | 20 | Amount | Independent | yes |
| 27 | Thailand | b | *Numenius madagascariensis* | 0.19 | 20 | Amount | Independent | yes |
| 27 | Thailand | b | *Numenius phaeopus* | -0.13 | 20 | Amount | Independent | yes |
| 27 | Thailand | b | *Philomachus pugnax* | 0.27 | 20 | Amount | Independent | yes |
| 27 | Thailand | b | *Pluvialis fulva* | 0.1 | 20 | Amount | Independent | yes |
| 27 | Thailand | b | *Pluvialis squatarola* | -0.17 | 20 | Amount | Independent | yes |
| 27 | Thailand | b | *Tringa erythropus* | 0.48 | 20 | Amount | Independent | yes |
| 27 | Thailand | b | *Tringa glareola* | -0.13 | 20 | Amount | Independent | yes |
| 27 | Thailand | b | *Tringa nebularia* | 0.19 | 20 | Amount | Independent | yes |
| 27 | Thailand | b | *Tringa stagnatilis* | 0.36 | 20 | Amount | Independent | yes |
| 27 | Thailand | b | *Tringa totanus* | 0.11 | 20 | Amount | Independent | yes |
| 27 | Thailand | b | *Xenus cinereus* | 0.01 | 20 | Amount | Independent | yes |
| 27 | Thailand | b | *Charadrius alexandrinus* | 0.17 | 20 | Amount | Independent | yes |
| 27 | Thailand | b | *Calidris alba* | 0.25 | 20 | Amount | Independent | yes |
| 27 | Thailand | b | *Calidris temminckii* | 0.42 | 20 | Amount | Independent | yes |
| 28 | USA | b | *Calidris alpina* | 0.46 | 5 | Amount | Dependent | yes |
| 29 | France | b | *Nycticorax nycticorax* | 0.2 | 25 | Amount | Dependent | yes |
| 29 | France | b | *Egretta garzetta* | -0.49 | 25 | Amount | Dependent | yes |
| 29 | France | b | *Bubulcus ibis* | 0.37 | 25 | Amount | Dependent | yes |
| 29 | France | b | *Ardeola ralloides* | 0.26 | 25 | Amount | Dependent | yes |
| 30 | Canada | b | *Ixobrychus exilis* | 0.64 | 15 | Amount | Independent | no |
| 30 | Canada | b | *Melospiza georgiana* | 0.47 | 15 | Amount | Independent | no |
| 30 | Canada | b | *Cistothorus palustris* | 0.61 | 15 | Amount | Independent | no |
| 30 | Canada | b | *Agelaius phoeniceus* | 0.44 | 15 | Amount | Independent | no |
| 30 | Canada | b | *Rallus limicola* | 0.2 | 15 | Amount | Independent | no |
| 30 | Canada | b | *Geothlypis trichas* | -0.31 | 15 | Amount | Independent | no |
| 30 | Canada | b | *Gallinula galeata* | 0.42 | 15 | Amount | Independent | no |
| 30 | Canada | b | *Porzana carolina* | 0.13 | 15 | Amount | Independent | no |
| 31 | USA | b | *Anas wyvilliana* | 0.34 | 5 | Amount | Dependent | yes |
| 32 | USA | b | *Ixobrychus exilis* | -0.08 | 62 | Amount | Independent | yes |
| 32 | USA | b | *Porphyrio martinica* | 0.01 | 109 | Amount | Independent | yes |
| 32 | USA | b | *Gallinula galeata* | 0.01 | 109 | Amount | Independent | yes |
| 33 | USA | b | *Grus canadensis* | 0.56 | 10 | Amount | Dependent | yes |
| 33 | USA | b | *Chlidonias niger* | 0.08 | 22 | Amount | Dependent | yes |
| 33 | USA | b | *Geothlypis trichas* | 0.65 | 32 | Amount | Dependent | yes |
| 33 | USA | b | *Empidonax alnorum* | 0.12 | 10 | Amount | Dependent | yes |
| 33 | USA | b | *Melospiza georgiana* | 0.49 | 32 | Amount | Dependent | yes |
| 33 | USA | b | *Xanthocephalus xanthocephalus* | 0.04 | 12 | Amount | Dependent | yes |
| 33 | USA | b | *Gallinago delicata* | 0.2 | 10 | Amount | Dependent | yes |
| 33 | USA | b | *Setophaga petechia* | 0.36 | 32 | Amount | Dependent | yes |
| 33 | USA | b | *Cistothorus palustris* | 0.42 | 22 | Amount | Dependent | yes |
| 33 | USA | b | *Agelaius phoeniceus* | -0.35 | 32 | Amount | Dependent | yes |
| 33 | USA | b | *Podilymbus podiceps* | 0.09 | 22 | Amount | Dependent | yes |
| 33 | USA | b | *Cistothorus platensis* | 0.36 | 22 | Amount | Dependent | yes |
| 33 | USA | b | *Rallus limicola* | -0.25 | 22 | Amount | Dependent | yes |
| 33 | USA | b | *Fulica americana* | -0.05 | 22 | Amount | Dependent | yes |
| 33 | USA | b | *Anas platyrhynchos* | 0.08 | 32 | Amount | Dependent | yes |
| 33 | USA | b | *Anas discors* | 0.06 | 22 | Amount | Dependent | yes |
| 33 | USA | b | *Porzana carolina* | 0.2 | 32 | Amount | Dependent | yes |
| 34 | USA | r | *Emydoidea blandingii* | 0.09 | 7 | Configuration | Dependent | no |
| 34 | USA | r | *Nerodia erythrogaster neglecta* | 0.13 | 12 | Configuration | Dependent | no |
| 34 | USA | r | *Nerodia sipedon sipedon* | 0.21 | 21 | Configuration | Dependent | no |
| 34 | USA | r | *Chrysemys picta marginata* | 0.26 | 31 | Configuration | Dependent | no |
| 35 | USA | r | *Chrysemys picta* | 0.4 | 80 | Configuration | Dependent | no |
| 36 | Canada | r | *Chrysemys picta marginata* | -0.19 | 20 | Amount | Dependent | yes |
| 37 | USA | r | *Emydoidea blandingii* | 0.5 | 6 | Amount | Dependent | yes |
| 37 | USA | r | *Clemmys guttata* | 0.43 | 10 | Amount | Dependent | yes |
| 38 | USA | r | *Chrysemys picta* | 0.22 | 35 | Amount | Independent | no |
| 39 | USA | r | *Nerodia erythrogaster neglecta* | 0.12 | 76 | Amount | Independent | yes |
| 39 | USA | r | *Nerodia rhombifer rhombifer* | 0.26 | 76 | Amount | Independent | yes |
| 39 | USA | r | *Nerodia sipedon pleuralis* | 0.04 | 76 | Amount | Independent | yes |
| 40 | USA | r | *Glyptemys muhlenbergii* | 0.09 | 43 | Amount | Unknown | yes |
| 24 | Canada | r | *Sternotherus odoratus* | -0.21 | 66 | Amount | Independent | yes |
| 24 | Canada | r | *Emydoidea blandingii* | -0.08 | 70 | Amount | Independent | yes |
| 41 | USA | r | *Apalone spinifera spinifera* | -0.09 | 30 | Amount | Independent | yes |
| 41 | USA | r | *Trachemys scripta elegans* | 0.21 | 55 | Amount | Independent | yes |
| 41 | USA | r | *Chelydra serpentina serpentina* | -0.13 | 150 | Amount | Independent | yes |
| 41 | USA | r | *Chrysemys picta marginata* | 0.16 | 206 | Amount | Independent | yes |
| 42 | Australia | r | *Chelodina longicollis* | 0.23 | 9 | Configuration | Dependent | no |
| 43 | USA | r | *Chelydra serpentina* | 0.04 | 25 | Amount | Independent | no |
| 43 | USA | r | *Chrysemys picta* | 0.18 | 25 | Amount | Independent | no |
| 44 | USA | r | *Chelydra serpentina* | -0.06 | 42 | Amount | Independent | no |
| 44 | USA | r | *Chrysemys picta* | -0.06 | 42 | Amount | Independent | no |
| 45 | USA | a | *Ambystoma maculatum* | -0.23 | 56 | Configuration | Dependent | no |
| 45 | USA | a | *Rana sylvatica* | -0.29 | 56 | Configuration | Dependent | no |
| 1 | Canada | a | *Bufo boreas* | -0.07 | 9 | Amount | Independent | no |
| 1 | Canada | a | *Pseudacris maculata* | -0.27 | 15 | Amount | Independent | no |
| 1 | Canada | a | *Rana sylvatica* | -0.27 | 15 | Amount | Independent | no |
| 46 | Switzerland | a | *Bufo bufo* | -0.3 | 34 | Configuration | Dependent | yes |
| 46 | Switzerland | a | *Triturus cristatus* | 0.11 | 38 | Configuration | Dependent | yes |
| 46 | Switzerland | a | *Rana temporaria* | -0.15 | 44 | Configuration | Dependent | yes |
| 46 | Switzerland | a | *Hyla arborea* | 0.27 | 46 | Configuration | Dependent | yes |
| 46 | Switzerland | a | *Ichthyosaura alpestris* | 0.14 | 49 | Configuration | Dependent | yes |
| 46 | Switzerland | a | *Rana esculenta* | 0.28 | 52 | Configuration | Dependent | yes |
| 46 | Switzerland | a | *Rana lessonae* | 0.3 | 52 | Configuration | Dependent | yes |
| 46 | Switzerland | a | *Lissotriton vulgaris* | 0.27 | 66 | Configuration | Dependent | yes |
| 47 | USA | a | *Hemidactylium scutatum* | -0.27 | 7 | Configuration | Dependent | yes |
| 48 | USA | a | *Ambystoma tigrinum tigrinum* | 0.19 | 89 | Configuration | Dependent | no |
| 49 | USA | a | *Rana catesbeiana* | 0.36 | 29 | Amount | Dependent | yes |
| 49 | USA | a | *Rana palustris* | 0.08 | 29 | Amount | Dependent | yes |
| 49 | USA | a | *Pseudacris crucifer* | 0.21 | 39 | Amount | Dependent | yes |
| 49 | USA | a | *Rana clamitans* | 0.15 | 39 | Amount | Dependent | yes |
| 49 | USA | a | *Ambystoma maculatum* | 0.11 | 45 | Amount | Dependent | yes |
| 49 | USA | a | *Rana sylvatica* | 0.02 | 45 | Amount | Dependent | yes |
| 49 | USA | a | *Notophthalmus viridescens* | 0.19 | 67 | Amount | Dependent | yes |
| 50 | USA | a | *Rana draytonii* | 0.37 | 22 | Configuration | Dependent | no |
| 50 | USA | a | *Rana catesbeiana* | 0.58 | 28 | Configuration | Dependent | no |
| 50 | USA | a | *Pseudacris regilla* | -0.14 | 32 | Configuration | Independent | no |
| 51 | Belgium | a | *Ichthyosaura alpestris* | -0.01 | 254 | Configuration | Dependent | yes |
| 51 | Belgium | a | *Lissotriton vulgaris* | 0.08 | 254 | Configuration | Dependent | yes |
| 52 | France | a | *Lissotriton helveticus* | -0.08 | 101 | Configuration | Dependent | yes |
| 53 | Canada | a | *Bufo americanus* | -0.12 | 36 | Amount | Independent | no |
| 53 | Canada | a | *Hyla versicolor* | 0.22 | 36 | Amount | Independent | no |
| 53 | Canada | a | *Pseudacris crucifer* | 0.33 | 36 | Amount | Independent | no |
| 53 | Canada | a | *Rana clamitans* | 0.13 | 36 | Amount | Dependent | no |
| 53 | Canada | a | *Rana pipiens* | -0.16 | 36 | Amount | Independent | no |
| 53 | Canada | a | *Rana sylvatica* | 0.05 | 36 | Amount | Independent | no |
| 54 | USA | a | *Rana luteiventris* | -0.16 | 95 | Amount | Independent | no |
| 54 | USA | a | *Pseudacris regilla* | -0.19 | 99 | Amount | Independent | no |
| 54 | USA | a | *Ambystoma macrodactylum* | -0.06 | 105 | Amount | Independent | no |
| 55 | USA | a | *Ambystoma tigrinum mavortium* | -0.35 | 12 | Amount | Dependent | yes |
| 55 | USA | a | *Bufo cognatus* | -0.07 | 12 | Amount | Dependent | yes |
| 55 | USA | a | *Spea bombifrons* | 0.59 | 12 | Amount | Dependent | yes |
| 55 | USA | a | *Spea multiplicata* | 0.51 | 12 | Amount | Dependent | yes |
| 56 | USA | a | *Ambystoma laterale* | 0.04 | 89 | Amount | Dependent | yes |
| 56 | USA | a | *Ambystoma maculatum* | -0.08 | 89 | Amount | Dependent | yes |
| 56 | USA | a | *Bufo americanus* | 0.22 | 89 | Amount | Independent | yes |
| 56 | USA | a | *Notopthalmus viridescens* | -0.11 | 89 | Amount | Dependent | yes |
| 56 | USA | a | *Rana clamitans* | 0.11 | 89 | Amount | Independent | yes |
| 56 | USA | a | *Rana pipiens* | 0.18 | 89 | Amount | Independent | yes |
| 56 | USA | a | *Rana septentrionalis* | 0.17 | 89 | Amount | Independent | yes |
| 56 | USA | a | *Rana sylvatica* | -0.13 | 89 | Amount | Independent | yes |
| 57 | Sweden | a | *Triturus cristatus* | 0.11 | 134 | Amount | Independent | no |
| 58 | Australia | a | *Limnodynastes dumerilii* | 0.13 | 36 | Amount | Independent | yes |
| 58 | Australia | a | *Limnodynastes peronii* | 0.29 | 36 | Amount | Independent | yes |
| 58 | Australia | a | *Litoria ewingii/verreauxii* | -0.02 | 36 | Amount | Independent | yes |
| 58 | Australia | a | *Crinia signifera* | 0.11 | 44 | Amount | Independent | yes |
| 58 | Australia | a | *Paracrinia haswelli* | 0.1 | 44 | Amount | Independent | yes |
| 59 | Australia | a | *Crinia signifera* | 0.15 | 30 | Amount | Dependent | yes |
| 59 | Australia | a | *Limnodynastes dumerilii* | 0.07 | 30 | Amount | Dependent | yes |
| 59 | Australia | a | *Limnodynastes peronii* | -0.17 | 30 | Amount | Dependent | yes |
| 59 | Australia | a | *Limnodynastes tasmaniensis* | 0.18 | 30 | Amount | Dependent | yes |
| 59 | Australia | a | *Litoria ewingii* | -0.31 | 30 | Amount | Dependent | yes |
| 59 | Australia | a | *Litoria fallax* | -0.06 | 30 | Amount | Dependent | yes |
| 59 | Australia | a | *Litoria raniformis* | -0.11 | 30 | Amount | Dependent | yes |
| 59 | Australia | a | *Litoria verreauxii* | -0.09 | 30 | Amount | Dependent | yes |
| 60 | Romania | a | *Bombina variegata* | 0.11 | 54 | Amount | Dependent | no |
| 60 | Romania | a | *Bufo bufo* | 0.16 | 54 | Amount | Dependent | no |
| 60 | Romania | a | *Hyla arborea* | -0.05 | 54 | Amount | Dependent | no |
| 60 | Romania | a | *Lissotriton vulgaris* | -0.22 | 54 | Amount | Dependent | no |
| 60 | Romania | a | *Pelobates fuscus* | -0.04 | 54 | Amount | Dependent | no |
| 60 | Romania | a | *Rana dalmatina* | -0.12 | 54 | Amount | Dependent | no |
| 60 | Romania | a | *Rana esculenta* | 0.12 | 54 | Amount | Dependent | no |
| 60 | Romania | a | *Rana temporaria* | -0.27 | 54 | Amount | Dependent | no |
| 60 | Romania | a | *Triturus cristatus* | -0.24 | 54 | Amount | Dependent | no |
| 61 | Australia | a | *Litoria raniformis* | 0.54 | 107 | Configuration | Dependent | no |
| 62 | Canada | a | *Bufo americanus* | 0.17 | 34 | Configuration | Dependent | no |
| 62 | Canada | a | *Hyla versicolor* | 0.06 | 34 | Configuration | Dependent | no |
| 62 | Canada | a | *Notophthalmus viridescens* | 0.11 | 34 | Configuration | Dependent | no |
| 62 | Canada | a | *Pseudacris crucifer* | 0.11 | 34 | Configuration | Dependent | no |
| 62 | Canada | a | *Pseudacris triseriata* | 0.17 | 34 | Configuration | Dependent | no |
| 62 | Canada | a | *Rana clamitans* | 0.13 | 34 | Configuration | Dependent | no |
| 62 | Canada | a | *Rana pipiens* | 0.16 | 34 | Configuration | Dependent | no |
| 62 | Canada | a | *Rana sylvatica* | 0 | 34 | Configuration | Dependent | no |
| 63 | USA | a | *Ambystoma maculatum* | 0.07 | 62 | Amount | Unknown | no |
| 63 | USA | a | *Rana sylvatica* | -0.11 | 62 | Amount | Unknown | no |
| 64 | Canada | a | *Ambystoma laterale* | 0.11 | 46 | Amount | Dependent | no |
| 64 | Canada | a | *Ambystoma maculatum* | 0.32 | 46 | Amount | Dependent | no |
| 64 | Canada | a | *Bufo americanus* | 0.09 | 46 | Amount | Dependent | no |
| 64 | Canada | a | *Hyla versicolor* | 0.35 | 46 | Amount | Dependent | no |
| 64 | Canada | a | *Notophthalmus viridescens* | 0.16 | 46 | Amount | Dependent | no |
| 64 | Canada | a | *Pseudacris crucifer* | 0.12 | 46 | Amount | Dependent | no |
| 64 | Canada | a | *Pseudacris triseriata* | 0.25 | 46 | Amount | Dependent | no |
| 64 | Canada | a | *Rana catesbeiana* | 0.16 | 46 | Amount | Dependent | no |
| 64 | Canada | a | *Rana clamitans* | 0.13 | 46 | Amount | Dependent | no |
| 64 | Canada | a | *Rana pipiens* | 0.19 | 46 | Amount | Dependent | no |
| 64 | Canada | a | *Rana septentrionalis* | 0.5 | 46 | Amount | Dependent | no |
| 64 | Canada | a | *Rana sylvatica* | 0.26 | 46 | Amount | Dependent | no |
| 65 | Canada | a | *Ambystoma laterale/A. maculatum* | -0.28 | 34 | Amount | Dependent | no |
| 65 | Canada | a | *Bufo americanus* | 0.18 | 34 | Amount | Independent | no |
| 65 | Canada | a | *Notopthalmus viridescens* | 0.47 | 34 | Amount | Dependent | no |
| 65 | Canada | a | *Pseudacris crucifer* | -0.23 | 34 | Amount | Independent | no |
| 65 | Canada | a | *Rana catesbeiana* | -0.39 | 34 | Amount | Dependent | no |
| 65 | Canada | a | *Rana clamitans* | 0.23 | 34 | Amount | Dependent | no |
| 65 | Canada | a | *Rana palustris* | 0.33 | 34 | Amount | Dependent | no |
| 65 | Canada | a | *Rana septentrionalis* | 0.46 | 34 | Amount | Dependent | no |
| 65 | Canada | a | *Rana sylvatica* | -0.25 | 34 | Amount | Dependent | no |
| 66 | USA | a | *Rana pipiens* | -0.06 | 31 | Amount | Independent | no |
| 67 | France | a | *Ichthyosaura alpestris* | 0.13 | 79 | Configuration | Independent | yes |
| 67 | France | a | *Lissotriton helveticus* | 0.24 | 79 | Configuration | Independent | yes |
| 67 | France | a | *Triturus cristatus* | 0.3 | 79 | Configuration | Independent | yes |
| 68 | USA | a | *Rana muscosa* | 0.12 | 1231 | Amount | Dependent | no |
| 69 | USA | a | *Rana palustris* | -0.15 | 22 | Amount | Independent | yes |
| 69 | USA | a | *Rana pipiens* | 0.19 | 22 | Amount | Independent | yes |
| 69 | USA | a | *Bufo americanus* | -0.13 | 27 | Amount | Independent | yes |
| 69 | USA | a | *Hyla versicolor* | -0.31 | 35 | Amount | Independent | yes |
| 69 | USA | a | *Pseudacris crucifer* | -0.18 | 37 | Amount | Independent | yes |
| 69 | USA | a | *Ambystoma tigrinum* | -0.24 | 39 | Amount | Independent | yes |
| 69 | USA | a | *Pseudacris triseriata* | -0.16 | 39 | Amount | Independent | yes |
| 69 | USA | a | *Rana clamitans* | 0.08 | 39 | Amount | Independent | yes |
| 70 | USA | a | *Hyla versicolor/H. chrysoscelis* | 0.26 | 21 | Amount | Independent | yes |
| 70 | USA | a | *Ambystoma tigrinum tigrinum* | -0.42 | 26 | Amount | Independent | yes |
| 70 | USA | a | *Bufo americanus* | -0.25 | 26 | Amount | Independent | yes |
| 70 | USA | a | *Bufo cognatus* | -0.38 | 26 | Amount | Independent | yes |
| 70 | USA | a | *Pseudacris triseriata* | 0.51 | 26 | Amount | Independent | yes |
| 70 | USA | a | *Rana pipiens* | 0.21 | 26 | Amount | Independent | yes |
| 70 | USA | a | *Rana sylvatica* | 0.29 | 26 | Amount | Independent | yes |
| 71 | Panama | a | *Engystomops pustulosus* | 0.51 | 7 | Configuration | Unknown | no |
| 72 | Canada | a | *Rana clamitans* | 0.13 | 21 | Amount | Dependent | yes |
| 73 | USA | a | *Bufo fowleri* | -0.05 | 22 | Amount | Independent | no |
| 73 | USA | a | *Hyla versicolor* | -0.08 | 22 | Amount | Independent | no |
| 73 | USA | a | *Pseudacris crucifer* | 0.33 | 22 | Amount | Independent | no |
| 73 | USA | a | *Rana catesbeiana* | 0.14 | 22 | Amount | Independent | no |
| 74 | USA | a | *Rana virgatipes* | 0.32 | 38 | Amount | Independent | no |
| 75 | Switzerland | a | *Hyla arborea* | 0.08 | 68 | Amount | Independent | no |
| 76 | Switzerland | a | *Hyla arborea* | 0.2 | 68 | Configuration | Independent | no |
| 77 | USA | a | *Acris crepitans* | 0.13 | 61 | Amount | Independent | no |
| 77 | USA | a | *Bufo americanus* | -0.11 | 61 | Amount | Independent | no |
| 77 | USA | a | *Hyla chrysoscelis* | 0.05 | 61 | Amount | Independent | no |
| 77 | USA | a | *Hyla versicolor* | 0.12 | 61 | Amount | Independent | no |
| 77 | USA | a | *Pseudacris triseriata* | 0.06 | 61 | Amount | Independent | no |
| 77 | USA | a | *Rana catesbeiana* | -0.07 | 61 | Amount | Independent | no |
| 77 | USA | a | *Rana pipiens* | -0.05 | 61 | Amount | Independent | no |
| 78 | Romania | a | *Bufo bufo* | -0.07 | 26 | Amount | Dependent | yes |
| 78 | Romania | a | *Rana temporaria* | 0.13 | 44 | Amount | Dependent | yes |
| 78 | Romania | a | *Hyla arborea* | 0.18 | 45 | Amount | Dependent | yes |
| 78 | Romania | a | *Bombina variegata* | -0.02 | 51 | Amount | Dependent | yes |
| 78 | Romania | a | *Rana dalmatina* | 0.21 | 51 | Amount | Dependent | yes |
| 78 | Romania | a | *Rana esculenta* | 0.21 | 51 | Amount | Dependent | yes |
| 78 | Romania | a | *Triturus cristatus* | 0.35 | 52 | Amount | Dependent | yes |
| 78 | Romania | a | *Lissotriton vulgaris* | 0.54 | 53 | Amount | Dependent | yes |
| 78 | Romania | a | *Salamandra salamandra* | 0.56 | 53 | Amount | Dependent | yes |
| 79 | Switzerland | a | *Bufo bufo* | -0.32 | 61 | Configuration | Unknown | yes |
| 79 | Switzerland | a | *Bufo calamita* | 0.24 | 61 | Configuration | Unknown | yes |
| 79 | Switzerland | a | *Ichthyosaura alpestris* | 0.14 | 91 | Configuration | Unknown | yes |
| 80 | USA | a | *Bufo americanus/B. woodhouseii fowleri* | -0.06 | 27 | Configuration | Dependent | no |
| 80 | USA | a | *Hyla versicolor* | -0.14 | 27 | Configuration | Dependent | no |
| 80 | USA | a | *Rana catesbeiana* | 0.05 | 27 | Configuration | Dependent | no |
| 80 | USA | a | *Rana palustris* | -0.38 | 27 | Configuration | Dependent | no |
| 80 | USA | a | *Ambystoma maculatum/A. jeffersonianum* | 0.44 | 34 | Configuration | Dependent | no |
| 80 | USA | a | *Notophthalmus viridescens* | -0.01 | 34 | Configuration | Dependent | no |
| 80 | USA | a | *Pseudacris crucifer* | 0 | 34 | Configuration | Dependent | no |
| 80 | USA | a | *Rana clamitans* | -0.13 | 34 | Configuration | Dependent | no |
| 80 | USA | a | *Rana sylvatica* | 0.37 | 34 | Configuration | Dependent | no |
| 81 | USA | a | *Rana pipiens* | 0.22 | 73 | Amount | Independent | no |
| 82 | USA | a | *Rana blairi/R. sphenocephalus* | 0.02 | 38 | Configuration | Dependent | no |
| 82 | USA | a | *Bufo americanus* | -0.34 | 40 | Configuration | Dependent | no |
| 82 | USA | a | *Rana catesbeiana* | 0.27 | 44 | Configuration | Dependent | no |
| 82 | USA | a | *Hyla versicolor/H. chrysoscelis* | 0.23 | 45 | Configuration | Dependent | no |
| 82 | USA | a | *Pseudacris crucifer* | 0.02 | 45 | Configuration | Dependent | no |
| 82 | USA | a | *Pseudacris maculata* | -0.09 | 45 | Configuration | Dependent | no |
| 82 | USA | a | *Rana clamitans* | 0.03 | 48 | Configuration | Dependent | no |
| 82 | USA | a | *Acris crepitans* | 0.12 | 49 | Configuration | Dependent | no |
| 82 | USA | a | *Ambystoma texanum* | 0.17 | 49 | Configuration | Dependent | no |
| 83 | Brazil | a | *Dendropsophus elianeae* | 0.14 | 6 | Configuration | Dependent | no |
| 83 | Brazil | a | *Dendropsophus minutus* | 0.25 | 6 | Configuration | Dependent | no |
| 83 | Brazil | a | *Dendropsophus nanus* | 0.06 | 6 | Configuration | Dependent | no |
| 83 | Brazil | a | *Dermatonotus muelleri* | 0.24 | 6 | Configuration | Dependent | no |
| 83 | Brazil | a | *Elachistocleis bicolor* | 0.22 | 6 | Configuration | Dependent | no |
| 83 | Brazil | a | *Eupemphix nattereri* | -0.15 | 6 | Configuration | Dependent | no |
| 83 | Brazil | a | *Hypsiboas albopunctatus* | 0 | 6 | Configuration | Dependent | no |
| 83 | Brazil | a | *Hypsiboas raniceps* | -0.23 | 6 | Configuration | Dependent | no |
| 83 | Brazil | a | *Leptodactylus chaquensis* | 0.1 | 6 | Configuration | Dependent | no |
| 83 | Brazil | a | *Leptodactylus fuscus* | 0.27 | 6 | Configuration | Dependent | no |
| 83 | Brazil | a | *Leptodactylus labyrinthicus* | 0.08 | 6 | Configuration | Dependent | no |
| 83 | Brazil | a | *Leptodactylus latrans* | -0.27 | 6 | Configuration | Dependent | no |
| 83 | Brazil | a | *Leptodactylus mystacinus* | 0.67 | 6 | Configuration | Dependent | no |
| 83 | Brazil | a | *Leptodactylus podicipinus* | 0.18 | 6 | Configuration | Dependent | no |
| 83 | Brazil | a | *Physalaemus centralis* | 0.16 | 6 | Configuration | Dependent | no |
| 83 | Brazil | a | *Physalaemus cuvieri* | 0.21 | 6 | Configuration | Dependent | no |
| 83 | Brazil | a | *Pseudis platensis* | 0.14 | 6 | Configuration | Dependent | no |
| 83 | Brazil | a | *Pseudopaludicola falcipes* | -0.29 | 6 | Configuration | Dependent | no |
| 83 | Brazil | a | *Rhinella schneideri* | -0.11 | 6 | Configuration | Dependent | no |
| 83 | Brazil | a | *Scinax fuscomarginatus* | -0.21 | 6 | Configuration | Dependent | no |
| 83 | Brazil | a | *Scinax similis* | 0.21 | 6 | Configuration | Dependent | no |
| 83 | Brazil | a | *Scinax fuscovarius* | 0.59 | 17 | Configuration | Dependent | no |
| 84 | USA | a | *Rana palustris* | 0.2 | 12 | Amount | Dependent | no |
| 84 | USA | a | *Rana utricularia* | 0.35 | 12 | Amount | Dependent | no |
| 84 | USA | a | *Bufo americanus* | -0.26 | 17 | Amount | Dependent | no |
| 84 | USA | a | *Hyla versicolor/chrysoscelis* | 0.37 | 17 | Amount | Dependent | no |
| 84 | USA | a | *Notophthalmus viridescens* | 0.14 | 17 | Amount | Dependent | no |
| 84 | USA | a | *Pseudacris crucifer* | -0.28 | 17 | Amount | Dependent | no |
| 84 | USA | a | *Rana catesbeiana* | -0.2 | 17 | Amount | Dependent | no |
| 84 | USA | a | *Rana clamitans* | 0.11 | 17 | Amount | Dependent | no |
| 84 | USA | a | *Rana sylvatica* | 0.57 | 18 | Amount | Dependent | no |
| 84 | USA | a | *Acris crepitans* | 0.47 | 19 | Amount | Dependent | no |
| 84 | USA | a | *Bufo fowleri* | -0.25 | 19 | Amount | Dependent | no |
| 85 | USA | a | *Ambystoma maculatum* | 0.05 | 55 | Amount | Dependent | yes |
| 85 | USA | a | *Rana sylvatica* | 0.05 | 55 | Amount | Dependent | yes |
| 86 | Australia | a | *Crinia parinsignifera* | -0.05 | 30 | Amount | Independent | yes |
| 86 | Australia | a | *Crinia signifera* | -0.12 | 30 | Amount | Independent | yes |
| 86 | Australia | a | *Limnodynastes dumerilii* | -0.22 | 30 | Amount | Independent | yes |
| 86 | Australia | a | *Limnodynastes peronii* | -0.13 | 30 | Amount | Independent | yes |
| 86 | Australia | a | *Limnodynastes tasmaniensis* | -0.28 | 30 | Amount | Independent | yes |
| 86 | Australia | a | *Litoria ewingii/L. paraewingi* | 0.29 | 30 | Amount | Independent | yes |
| 86 | Australia | a | *Litoria peronii* | -0.1 | 30 | Amount | Independent | yes |
| 87 | USA | a | *Ambystoma maculatum* | 0.18 | 49 | Amount | Dependent | no |
| 87 | USA | a | *Rana sylvatica* | -0.1 | 49 | Amount | Dependent | no |
| 88 | Netherlands | a | *Rana arvalis* | 0.09 | 74 | Amount | Unknown | yes |
| 89 | Netherlands | a | *Hyla arborea* | 0.46 | 149 | Configuration | Unknown | yes |
| 90 | USA | a | *Pseudacris crucifer* | -0.11 | 13 | Configuration | Dependent | no |
| 90 | USA | a | *Pseudacris triseriata* | -0.15 | 26 | Configuration | Dependent | no |
| 91 | Switzerland | a | *Bufo bufo* | 0.07 | 107 | Amount | Unknown | yes |
| 91 | Switzerland | a | *Bufo calamita* | -0.1 | 247 | Amount | Unknown | yes |
| 91 | Switzerland | a | *Rana temporaria* | 0.1 | 296 | Amount | Unknown | yes |
| 91 | Switzerland | a | *Hyla arborea* | 0.22 | 315 | Amount | Unknown | yes |
| 91 | Switzerland | a | *Ichthyosaura alpestris* | -0.07 | 414 | Amount | Unknown | yes |
| 91 | Switzerland | a | *Rana ridibunda* | 0.06 | 423 | Amount | Unknown | yes |
| 91 | Switzerland | a | *Lissotriton helveticus* | -0.03 | 469 | Amount | Unknown | yes |
| 91 | Switzerland | a | *Bombina variegata* | -0.07 | 480 | Amount | Unknown | yes |
| 91 | Switzerland | a | *Rana dalmatina* | 0.14 | 520 | Amount | Unknown | yes |
| 91 | Switzerland | a | *Lissotriton vulgaris* | 0.12 | 615 | Amount | Unknown | yes |

*Studies cited in Reference List S1. Studies conducted in the same study area by the same researchers are identified as one study in the meta-analysis

^+^Taxa: m = mammal, b = bird, r = reptile, a = amphibian
