# Supplementary material for: Low Reproductive Rate Predicts Species Sensitivity to Habitat Loss: A Meta-Analysis of Wetland Vertebrates
Source: PLoS One. 2014 Mar 20;9(3):e90926. doi: 10.1371/journal.pone.0090926 (PMC3961235; doi:10.1371/journal.pone.0090926)
Supplement: Table S2 — Species traits and reference information for the 220 species used in the meta-analysis. (DOCX) [file pone.0090926.s004.docx]

Table S2: Species traits and reference information for the 220 species used in the meta-analysis.

| Study* | Taxa^+^ | Species | Order | Repro^a^ | Source^§^ | Home Range^b^ (ha) | Source | Mass^c^ (g) | Source | Length^d^ (cm) | Source |
| --- | --- | --- | --- | --- | --- | --- | --- | --- | --- | --- | --- |
| 1 | m | *Castor canadensis* | Rodentia | 3.0 | Baker and Hill 2003 | 13 | Baker and Hill 2003 | 23750 | Baker and Hill 2003 | 120 | Baker and Hill 2003 |
| 2 | m | *Oryzomys palustris* | Rodentia | 15.2 | Jones et al. 2009; Linzey and NatureServe 2008 | 0.73 | Eubanks et al. 2011 | 53.26 | Jones et al. 2009 | 13.35 | Jones et al. 2009 |
| 3 | m | *Microtus pennsylvanicus* | Rodentia | 33.8 | Linzey and NatureServe 2008 | 0.17 | Reich 1981 | 44.1 | Reich 1981 | 16.7 | Reich 1981 |
| 3 | m | *Sorex fumeus* | Rodentia | 8.5 | Jones et al. 2009 | 0.12 | Linzey 1983 | 35.7 | Linzey 1983 | 12.4 | Linzey 1983 |
| 3 | m | *Synaptomys cooperi* | Soricomorpha | 11.0 | Owen 1984; Weinstein 1999 | n/a |  | 7.6 | Owen 1984 | 11.1 | Owen 1984 |
| 4 | m | *Sylvilagus palustris hefneri* | Lagomorpha | 6.5 | Forys 1995 | 3.96 | Forys and Humphrey 1996 | 1224.1 | Forys 1995 | 33.9 | Forys 1995 |
| 5 | m | *Neofiber alleni* | Rodentia | 11.5 | Baker and Hill 2003 | 0.21 | Schooley and Branch 2006 | 270.5 | Baker and Hill 2003 | 33.3 | Baker and Hill 2003 |
| 6 | b | *Ardea cinerea* | Pelecaniformes | 4.5 | Martínez-Vilalta and Motis 1992 | 767.5 | van Vessem et al. 1984 | 1546.5 | Martínez-Vilalta and Motis 1992 | 94 | Martínez-Vilalta and Motis 1992 |
| 6 | b | *Egretta intermedia* | Pelecaniformes | 4.0 | Martínez-Vilalta and Motis 1992 | 28.26 | Amano and Katayama 2009 | 400 | Martínez-Vilalta and Motis 1992 | 64 | Martínez-Vilalta and Motis 1992 |
| 6 | b | *Nycticorax nycticorax* | Pelecaniformes | 4.0 | Martínez-Vilalta and Motis 1992 | 21.022 | Wong et al. 1999 | 662.5 | Martínez-Vilalta and Motis 1992 | 60.5 | Martínez-Vilalta and Motis 1992 |
| 7 | b | *Botaurus lentiginosus* | Ciconiiformes | 4.1 | Lowther et al. 2009 | 109.28 | Lor 2007 | 435 | Lowther et al. 2009 | 72.5 | Lowther et al. 2009 |
| 7 | b | *Ixobrychus exilis* | Ciconiiformes | 4.5 | Poole et al. 2009 | 223.2 | Griffin et al. 2009 | 80 | Poole et al. 2009 | 32 | Poole et al. 2009 |
| 8 | b | *Botaurus lentiginosus* | Ciconiiformes | 4.1 | Lowther et al. 2009 | 109.28 | Lor 2007 | 435 | Lowther et al. 2009 | 72.5 | Lowther et al. 2009 |
| 8 | b | *Fulica americana* | Gruiformes | 8.5 | Brisbin et al. 2002 | 0.15 | Brisbin et al. 2002 | 637.5 | Brisbin et al. 2002 | 37.5 | Brisbin et al. 2002 |
| 8 | b | *Ixobrychus exilis* | Ciconiiformes | 3.8 | Poole et al. 2009 | 223.2 | Griffin et al. 2009 | 80 | Poole et al. 2009 | 32 | Poole et al. 2009 |
| 8 | b | *Podilymbus podiceps* | Podicipediformes | 7.0 | Muller and Storer 1999 | 1.31 | Muller and Storer 1999 | 410.5 | Muller and Storer 1999 | 34.3 | Muller and Storer 1999 |
| 8 | b | *Porzana carolina* |  | 10.3 | Melvin and Gibbs 2012 | 0.19 | Johnson and Dinsmore 1985 | 80 | Melvin and Gibbs 2012 | 22.5 | Melvin and Gibbs 2012 |
| 9 | b | *Circus aeruginosus* | Falconiformes | 4.5 | Dijkstra and Zijlstra 1997; Orta 1994 | 2517.3 | Cardador et al. 2009 | 603 | Thiollay 1994 | 56.5 | Thiollay 1994 |
| 10 | b | *Acrocephalus scirpaceus* | Passeriformes | 7.8 | Catchpole 1974 | 0.033 | Catchpole 1972; Chernetsov and Titov 2001 | 12.3 | Dyrcz 2006 | 13 | Dyrcz 2006 |
| 11 | b | *Ardea herodia* | Ciconiiformes | 4.1 | Vennesland and Butler 2011 | 572.27 | Custer and Galli 2002 | 2300 | Vennesland and Butler 2011 | 117 | Vennesland and Butler 2011 |
| 12 | b | *Ardea herodia* | Ciconiiformes | 4.1 | Vennesland and Butler 2011 | 572.27 | Custer and Galli 2002 | 2300 | Vennesland and Butler 2011 | 117 | Vennesland and Butler 2011 |
| 13 | b | *Botaurus lentiginosus* | Ciconiiformes | 4.6 | Lor and Malecki. 2006 | 109.28 | Lor 2007 | 435 | Lowther et al. 2009 | 72.5 | Lowther et al. 2009 |
| 13 | b | *Butorides virescens* | Ciconiiformes | 3.9 | Davis and Kushlan 1994 | n/a |  | 241 | Davis and Kushlan 1994 | 43.5 | Davis and Kushlan 1994 |
| 13 | b | *Circus cyaneus* | Falconiformes | 4.4 | Smith et al. 2011 | 260 | Smith et al. 2011 | 458 | Smith et al. 2011 | 47.5 | Thiollay 1994 |
| 13 | b | *Gallinago delicata* | Charadriiformes | 3.9 | Mueller 1999 | 235 | Cline and Haig 2011 | 100 | Mueller 1999 | 28 | Mueller 1999 |
| 13 | b | *Podilymbus podiceps* | Podicipediformes | 6.8 | Muller and Storer 1999 | 1.31 | Muller and Storer 1999 | 410.5 | Muller and Storer 1999 | 34.3 | Muller and Storer 1999 |
| 13 | b | *Porzana carolina* | Gruiformes | 11.5 | Lor and Malecki 2006 | 0.19 | Johnson and Dinsmore 1985 | 80 | Melvin and Gibbs 2012 | 22.5 | Melvin and Gibbs 2012 |
| 13 | b | *Rallus limicola* | Gruiformes | 7.9 | Lor and Malecki 2006 | 0.18 | Johnson and Dinsmore 1985 | 87 | Conway 1995 | 24.5 | Conway 1995 |
| 14 | b | *Botaurus stellaris* | Ciconiiformes | 4.0 | Gilbert et al. 2007 | 21.437 | Gilbert et al. 2005 | 1403.5 | Martínez-Vilalta and Motis 1992 | 72 | Martínez-Vilalta and Motis 1992 |
| 15 | b | *Agelaius phoeniceus* | Passeriformes | 5.6 | Yasukawa and Searcy 1995 | 0.16 | Yasukawa and Searcy 1995 | 57.2 | Yasukawa and Searcy 1995 | 22 | Rosenthal 2004 |
| 15 | b | *Cistothorus palustris* | Passeriformes | 5.0 | Kroodsma and Verner 1997 | 0.1 | Leonard and Picman 1986; Verner 1965 | 11.5 | Kroodsma and Verner 1997 | 122 | Kroodsma and Verner 1997 |
| 15 | b | *Cistothorus platensis* | Passeriformes | 7.0 | Herkert et al. 2001 | 0.178 | Herkert et al. 2001 | 8.5 | Herkert et al. 2001 | 11 | Herkert et al. 2001 |
| 16 | b | *Amazonetta brasiliensis* | Anseriformes | 7.0 | Carboneras 1992b | n/a |  | 415 | Carboneras 1992b | 37.5 | Carboneras 1992b |
| 16 | b | *Anas flavirostris* | Anseriformes | 6.5 | Carboneras 1992b | n/a |  | 715 | Carboneras 1992b | 40 | Carboneras 1992b |
| 16 | b | *Anas versicolor* | Anseriformes | 8.0 | Carboneras 1992b | n/a |  | 407.5 | Carboneras 1992b | 40.5 | Carboneras 1992b |
| 16 | b | *Aramides ypecaha* | Gruiformes | 13.5 | Taylor 1996 | n/a |  | 765 | Taylor 1996 | 43 | Taylor 1996 |
| 16 | b | *Aramus guarauna* | Gruiformes | 11.0 | Bryan 1996, 2002 | 2.105 | Bryan 2002 | 1180 | Bryan 1996 | 63.5 | Bryan 1996 |
| 16 | b | *Ardea alba* | Ciconiiformes | 3.6 | Pretelli et al. 2012 | 1865.6 | Custer and Osborn 1978; Stolen et al. 2007 | 1100 | Martínez-Vilalta and Motis 1992 | 92 | Martínez-Vilalta and Motis 1992 |
| 16 | b | *Ardea cocoi* | Ciconiiformes | 3.0 | Martínez-Vilalta and Motis 1992 | n/a |  | n/a |  | 111 | Martínez-Vilalta and Motis 1992 |
| 16 | b | *Butorides striata* | Ciconiiformes | 3.5 | Martínez-Vilalta and Motis 1992 | n/a |  | 192.5 | Martínez-Vilalta and Motis 1992 | 40.5 | Martínez-Vilalta and Motis 1992 |
| 16 | b | *Callonetta leucophrys* | Anseriformes | 9.0 | Carboneras 1992b | n/a |  | 275 | Carboneras 1992b | 36.5 | Carboneras 1992b |
| 16 | b | *Chauna torquata* | Anseriformes | 4.0 | Carboneras 1992a | n/a |  | 4400 | Carboneras 1992a | 89 | Carboneras 1992a |
| 16 | b | *Ciconia maguari* | Ciconiiformes | 3.1 | González 1998 | n/a |  | n/a |  | 99.5 | Elliot 1992 |
| 16 | b | *Circus buffoni* | Falconiformes | 3.5 | Thiollay 1994 | n/a |  | 501 | Thiollay 1994 | 52 | Thiollay 1994 |
| 16 | b | *Dendrocygna bicolor* | Anseriformes | 10.0 | Carboneras 1992b | n/a |  | 688 | Carboneras 1992b | 49 | Carboneras 1992b |
| 16 | b | *Dendrocygna viduata* | Anseriformes | 8.5 | Carboneras 1992b | n/a |  | 661 | Carboneras 1992b | 43 | Carboneras 1992b |
| 16 | b | *Egretta thula* | Ciconiiformes | 2.9 | Petry and Da Silva Fonseca 2005 | 1734.1 | Stolen et al. 2007 | 370 | Martínez-Vilalta and Motis 1992 | 57.8 | Martínez-Vilalta and Motis 1992 |
| 16 | b | *Fulica leucoptera* | Gruiformes | 11.0 | Taylor 1996 | n/a |  | 528.5 | Taylor 1996 | 39 | Taylor 1996 |
| 16 | b | *Gallinago paraguaiae* | Charadriiformes | 4.0 | van Gils and Wiersma 1996 | n/a |  | 136 | van Gils and Wiersma 1996 | 25.5 | van Gils and Wiersma 1996 |
| 16 | b | *Gallinula galeata* | Gruiformes | 11.4 | McRae 2011, Taylor 1996 | n/a |  | 305 | Taylor 1996 | 34 | Taylor 1996 |
| 16 | b | *Gallinula melanops* | Gruiformes | 6.0 | Taylor 1996 | n/a |  | 154 | Taylor 1996 | 26 | Taylor 1996 |
| 16 | b | *Himantopus himantopus* | Charadriiformes | 4.0 | Pierce 1996 | 0.221 | Robinson et al. 1999 | 185.5 | Pierce 1996 | 37.5 | Pierce 1996 |
| 16 | b | *Jacana jacana* | Charadriiformes | 6.4 | Emlen et al. 1998 | 0.113 | Emlen et al. 1998 | 124.5 | Jenni 1996 | 23 | Jenni 1996 |
| 16 | b | *Mycteria americana* | Ciconiiformes | 3.0 | González 1999 | 7084.6 | Bryan et al. 2012 | 2500 | Elliot 1992 | 92.5 | Elliot 1992 |
| 16 | b | *Netta peposaca* | Anseriformes | 10.0 | Carboneras 1992b | n/a |  | 1100 | Carboneras 1992b | 55.5 | Carboneras 1992b |
| 16 | b | *Nycticorax nycticorax* | Ciconiiformes | 2.9 | Petry and Da Silva Fonseca 2005 | 21.022 | Wong et al. 1999 | 662.5 | Martínez-Vilalta and Motis 1992 | 60.5 | Martínez-Vilalta and Motis 1992 |
| 16 | b | *Pardirallus sanguinolentus* | Gruiformes | 5.0 | Taylor 1996 | n/a |  | 212.3 | Taylor 1996 | 34 | Taylor 1996 |
| 16 | b | *Phimosus infuscatus* | Ciconiiformes | 3.1 | Petry and Da Silva Fonseca 2005 | n/a |  | n/a |  | 50 | Matheu and del Hoyo 1992 |
| 16 | b | *Platalea ajaja* | Ciconiiformes | 2.5 | Matheu and del Hoyo 1992 | 13070 | Dumas 2000 | 1400 | Matheu and del Hoyo 1992 | 77.5 | Matheu and del Hoyo 1992 |
| 16 | b | *Plegadis chihi* | Ciconiiformes | 2.3 | Petry and Da Silva Fonseca 2005 | 1589.6 | Bray and Klebenow 1988 | 612.5 | Ryder and Manry 1994 | 56 | Matheu and del Hoyo 1992 |
| 16 | b | *Podilymbus podiceps* | Podicipediformes | 16.5 | Llimona and del Hoyo 1992 | 1.31 | Muller and Storer 1999 | 398.5 | Llimona and del Hoyo 1992 | 34 | Llimona and del Hoyo 1992 |
| 16 | b | *Rollandia rolland* | Podicipediformes | 6.0 | Burger 1974; Llimona and del Hoyo 1992 | 0.002 | Burger 1974 | n/a |  | 30 | Llimona and del Hoyo 1992 |
| 16 | b | *Rostrhamus sociabilis* | Falconiformes | 9.0 | Angehr 1999; Thiollay 1994 | 1163.6 | Beissinger and Snyder 1987 | 376.5 | Thiollay 1994 | 42.5 | Thiollay 1994 |
| 16 | b | *Syrigma sibilatrix* | Ciconiiformes | 3.0 | Martínez-Vilalta and Motis 1992 | n/a |  | n/a |  | 55.5 | Martínez-Vilalta and Motis 1992 |
| 16 | b | *Theristicus caerulescens* | Ciconiiformes | 2.5 | Matheu and del Hoyo 1992 | n/a |  | n/a |  | 73.5 | Matheu and del Hoyo 1992 |
| 17 | b | *Botaurus lentiginosus* | Ciconiiformes | 4 | Lowther et al. 2009 | 109.28 | Lor 2007 | 435 | Lowther et al. 2009 | 72.5 | Lowther et al. 2009 |
| 17 | b | *Ixobrychus exilis* | Ciconiiformes | 3.8 | Poole et al. 2009 | 9.7 | Bogner and Baldassarre 2002 | 80 | Poole et al. 2009 | 32 | Poole et al. 2009 |
| 17 | b | *Podilymbus podiceps* | Podicipediformes | 7.3 | Muller and Storer 1999 | 1.31 | Muller and Storer 1999 | 410.5 | Muller and Storer 1999 | 34.3 | Muller and Storer 1999 |
| 17 | b | *Porzana carolina* | Gruiformes | 12.2 | Melvin and Gibbs 2012 | 0.19 | Johnson and Dinsmore 1985 | 80 | Melvin and Gibbs 2012 | 22.5 | Melvin and Gibbs 2012 |
| 17 | b | *Rallus limicola* | Gruiformes | 8.6 | Kaufmann 1989 | 0.18 | Johnson and Dinsmore 1985 | 87 | Conway 1995 | 24.5 | Conway 1995 |
| 18 | b | *Ardea alba* | Ciconiiformes | 2.9 | Pratt and Winkler 1985 | 1865.6 | Custer and Osborn 1978, Stolen et al. 2007 | 1000 | Mccrimmon et al. 2011 | 99 | Mccrimmon et al. 2011 |
| 18 | b | *Ardea herodias* | Ciconiiformes | 3.2 | Pratt and Winkler 1985 | 452.16 | Butler 1991 | 2300 | Vennesland and Butler 2011 | 117 | Vennesland and Butler 2011 |
| 19 | b | *Acrocephalus rufescens* | Passeriformes | 2.5 | Dyrcz 2006, Urban et al. 1997 | 0.063 | Urban et al. 1997 | 22.8 | Dyrcz 2006 | 17 | Dyrcz 2006 |
| 19 | b | *Bradypterus carpalis* | Passeriformes | 2.5 | Pearson 2006, Urban et al. 1997 | n/a |  | 14 | Pearson 2006 | 14 | Pearson 2006 |
| 19 | b | *Chloropeta gracilirostris* | Passeriformes | 2.4 | Jetz et al. 2008 | n/a |  | 14.4 | Pearson 2006 | 13.5 | Pearson 2006 |
| 19 | b | *Cisticola carruthersi* | Passeriformes | 3 | Madge 2006, Urban et al. 1997 | n/a |  | 11.3 | Madge 2006 | 12.5 | Madge 2006 |
| 19 | b | *Laniarius mufumbiri* | Passeriformes | 2 | Jetz et al. 2008 | n/a |  | 40 | Fry 2009 | 18 | Fry 2009 |
| 19 | b | *Serinus koliensis* | Passeriformes | 1.5 | Collar et al. 2010, Fry and Keith 2004 | n/a |  | 13.5 | Collar et al. 2010 | 10.8 | Collar et al. 2010 |
| 20 | b | *Circus cyaneus* | Falconiformes | 4.4 | Smith et al. 2011 | 260 | Smith et al. 2011 | 458 | Smith et al. 2011 | 47.5 | Thiollay 1994 |
| 21 | b | *Acrocephalus arundinaceus* | Passeriformes | 5 | Dyrcz 2006 | 0.405 | Catchpole et al. 1985 | 27.2 | Dyrcz 2006 | 19.5 | Dyrcz 2006 |
| 21 | b | *Acrocephalus scirpaceus* | Passeriformes | 7.8 | Catchpole 1974 | 0.033 | Catchpole 1972, Chernetsov and Titov 2001 | 12.3 | Dyrcz 2006 | 13 | Dyrcz 2006 |
| 21 | b | *Cettia cetti* | Passeriformes | 6 | Clement 2006 | 1.275 | Clement 2006 | 13.3 | Clement 2006 | 14 | Clement 2006 |
| 21 | b | *Circus aeruginosus* | Falconiformes | 4.5 | Dijkstra and Zijlstra 1997; Orta 1994 | 2517.3 | Cardador et al. 2009 | 603 | Thiollay 1994 | 56.5 | Thiollay 1994 |
| 21 | b | *Rallus aquaticus* | Gruiformes | 17 | Taylor 1992 | 0.159 | Jenkins et al. 1995 | 115 | Taylor 1992 | 26.5 | Taylor 1992 |
| 21 | b | *Remiz pendulinus* | Passeriformes | 5 | Madge 2008; Pogány et al. 2012 | 87.87 | Mészáros et al. 2006 | 10.3 | Madge 2008 | 10.5 | Madge 2008 |
| 22 | b | *Limosa fedoa* | Charadriiformes | 4 | Gratto-Trevor 2000 | 90 | Gratto-Trevor 2000 | 369.5 | Gratto-Trevor 2000 | 45 | Gratto-Trevor 2000 |
| 22 | b | *Phalaropus tricolor* | Charadriiformes | 4 | Colwell and Jehl 1994 | 78.5 | Colwell and Jehl 1994 | 62.1 | Colwell and Jehl 1994 | 23 | Colwell and Jehl 1994 |
| 22 | b | *Recurvirostra americana* | Charadriiformes | 4 | Robinson et al. 1997 | 1316 | Demers et al. 2008 | 312.5 | Robinson et al. 1997 | 45 | Robinson et al. 1997 |
| 22 | b | *Tringa semipalmata* | Charadriiformes | 4 | Lowther et al. 2001 | 44.3 | Ryan and Renken 1987 | 265 | Lowther et al. 2001 | 37 | Lowther et al. 2001 |
| 23 | b | *Dendrocygna bicolor* | Anseriformes | 12.4 | Pierluissi 2006 | n/a |  | 765.5 | Hohman and Lee 2001 | 46.5 | Hohman and Lee 2001 |
| 23 | b | *Gallinula galeata* | Gruiformes | 8.4 | Pierluissi 2006 | 1.22 | Matthews 1983 | 383 | Bannor and Kiviat 2002 | 33.5 | Bannor and Kiviat 2002 |
| 23 | b | *Ixobrychus exilis* | Ciconiiformes | 4.2 | Pierluissi 2006 | 223.2 | Griffin et al. 2009 | 80 | Poole et al. 2009 | 32 | Poole et al. 2009 |
| 23 | b | *Porphyrio martinica* | Gruiformes | 7.8 | Pierluissi 2006 | 1.03 | West and Hess 2002 | 248 | West and Hess 2002 | 31.5 | West and Hess 2002 |
| 23 | b | *Rallus elegans* | Gruiformes | 9.1 | Pierluissi 2006 | 14.53 | Pickens 2012 | 337.3 | Poole et al. 2005 | 43 | Poole et al. 2005 |
| 24 | b | *Cistothorus palustris* | Passeriformes | 5 | Kroodsma and Verner 1997 | 0.1 | Leonard and Picman 1986, Verner 1965 | 11.5 | Kroodsma and Verner 1997 | 122 | Kroodsma and Verner 1997 |
| 24 | b | *Geothlypis trichas* | Passeriformes | 8 | Abroe et al. 2007 | 1.2 | Guzy and Ritchison 1999 | 9.5 | Guzy and Ritchison 1999 | 12 | Guzy and Ritchison 1999 |
| 24 | b | *Ixobrychus exilis* | Ciconiiformes | 3.8 | Poole et al. 2009 | 9.7 | Bogner and Baldassarre 2002 | 80 | Poole et al. 2009 | 32 | Poole et al. 2009 |
| 24 | b | *Melospiza georgiana* | Passeriformes | 3.8 | Mowbray 1997 | 0.17 | Mowbray 1997 | 17.8 | Mowbray 1997 | 13.5 | Mowbray 1997 |
| 24 | b | *Podilymbus podiceps* | Podicipediformes | 6.4 | Lor and Malecki 2006 | 1.31 | Muller and Storer 1999 | 410.5 | Muller and Storer 1999 | 34.3 | Muller and Storer 1999 |
| 24 | b | *Rallus limicola* | Gruiformes | 7.9 | Lor and Malecki 2006 | 0.18 | Johnson and Dinsmore 1985 | 87 | Conway 1995 | 24.5 | Conway 1995 |
| 24 | b | *Setophaga petechia* | Passeriformes | 3.8 | Lowther et al. 1999 | 0.284 | Lowther et al. 1999 | 10 | Lowther et al. 1999 | 12.5 | Lowther et al. 1999 |
| 25 | b | *Botaurus lentiginosus* | Ciconiiformes | 4.6 | Lor and Malecki 2006 | 109.28 | Lor 2007 | 435 | Lowther et al. 2009 | 72.5 | Lowther et al. 2009 |
| 25 | b | *Ixobrychus exilis* | Ciconiiformes | 5.3 | Bogner and Baldassarre 2002 | 9.7 | Bogner and Baldassarre 2002 | 80 | Poole et al. 2009 | 32 | Poole et al. 2009 |
| 25 | b | *Podilymbus podiceps* | Podicipediformes | 6.4 | Lor and Malecki 2006 | 1.31 | Muller and Storer 1999 | 410.5 | Muller and Storer 1999 | 34.3 | Muller and Storer 1999 |
| 25 | b | *Porzana carolina* | Gruiformes | 11.5 | Lor and Malecki 2006 | 0.19 | Johnson and Dinsmore 1985 | 80 | Melvin and Gibbs 2012 | 22.5 | Melvin and Gibbs 2012 |
| 25 | b | *Rallus limicola* | Gruiformes | 7.9 | Lor and Malecki 2006 | 0.18 | Johnson and Dinsmore 1985 | 87 | Conway 1995 | 24.5 | Conway 1995 |
| 26 | b | *Himantopus himantopus* | Charadriiformes | 4 | Cuervo 2005; José Javier Cuervo, personal communication | n/a |  | 185.5 | Pierce 1996 | 37.5 | Pierce 1996 |
| 26 | b | *Tachybaptus ruficollis* | Podicipediformes | 8 | Llimona and del Hoyo 1992 | 1.693 | Gutiérrez and Figuerola 1997 | 183 | Llimona and del Hoyo 1992 | 27 | Llimona and del Hoyo 1992 |
| 27 | b | *Actitis hypoleucos* | Charadriiformes | 4 | van Gils and Wiersma 1996 | n/a |  | 58.5 | van Gils and Wiersma 1996 | 20 | van Gils and Wiersma 1996 |
| 27 | b | *Arenaria interpres* | Charadriiformes | 4 | van Gils and Wiersma 1996 | 754.39 | Rehfisch et al. 2003 | 137 | van Gils and Wiersma 1996 | 23.5 | van Gils and Wiersma 1996 |
| 27 | b | *Calidris alba* | Charadriiformes | 8 | van Gils and Wiersma 1996 | 1962.5 | Macwhirter et al. 2002 | 71.5 | van Gils and Wiersma 1996 | 20.5 | van Gils and Wiersma 1996 |
| 27 | b | *Calidris canutus* | Charadriiformes | 3.5 | van Gils and Wiersma 1996 | 880 | Leyrer et al. 2006 | 152.5 | van Gils and Wiersma 1996 | 24 | van Gils and Wiersma 1996 |
| 27 | b | *Calidris ferruginea* | Charadriiformes | 3.8 | van Gils and Wiersma 1996 | n/a |  | 80.5 | van Gils and Wiersma 1996 | 20.5 | van Gils and Wiersma 1996 |
| 27 | b | *Calidris ruficollis* | Charadriiformes | 4 | van Gils and Wiersma 1996 | n/a |  | 34.5 | van Gils and Wiersma 1996 | 14.5 | van Gils and Wiersma 1996 |
| 27 | b | *Calidris subminuta* | Charadriiformes | 4 | van Gils and Wiersma 1996 | n/a |  | 28.5 | van Gils and Wiersma 1996 | 14.5 | van Gils and Wiersma 1996 |
| 27 | b | *Calidris temminckii* | Charadriiformes | 10 | van Gils and Wiersma 1996 | n/a |  | 25.5 | van Gils and Wiersma 1996 | 14 | van Gils and Wiersma 1996 |
| 27 | b | *Calidris tenuirostris* | Charadriiformes | 4 | van Gils and Wiersma 1996 | n/a |  | 181.5 | van Gils and Wiersma 1996 | 27 | van Gils and Wiersma 1996 |
| 27 | b | *Charadrius alexandrinus* | Charadriiformes | 6 | Wiersma 1992 | 36 | Brindock and Colwell 2011 | 44 | Wiersma 1992 | 16.3 | Wiersma 1992 |
| 27 | b | *Charadrius dubius* | Charadriiformes | 4 | Wiersma 1992 | n/a |  | 39.5 | Wiersma 1992 | 15.5 | Wiersma 1992 |
| 27 | b | *Charadrius mongolus/C. leschenaultii* | Charadriiformes | 3 | Wiersma 1992 | n/a |  | 81.3 | Wiersma 1992 | 21.5 | Wiersma 1992 |
| 27 | b | *Eurynorhynchus pygmeus* | Charadriiformes | 4 | van Gils and Wiersma 1996 | n/a |  | 31.8 | van Gils and Wiersma 1996 | 15 | van Gils and Wiersma 1996 |
| 27 | b | *Gallinago gallinago* | Charadriiformes | 4 | van Gils and Wiersma 1996 | n/a |  | 126.5 | van Gils and Wiersma 1996 | 26 | van Gils and Wiersma 1996 |
| 27 | b | *Limicola falcinellus* | Charadriiformes | 4 | van Gils and Wiersma 1996 | n/a |  | 48 | van Gils and Wiersma 1996 | 17 | van Gils and Wiersma 1996 |
| 27 | b | *Limnodromus semipalmatus* | Charadriiformes | 2 | van Gils and Wiersma 1996 | n/a |  | 86 | van Gils and Wiersma 1996 | 34.5 | van Gils and Wiersma 1996 |
| 27 | b | *Limosa lapponica* | Charadriiformes | 4 | van Gils and Wiersma 1996 | 1519.8 | Rehfisch et al. 2003 | 370.5 | van Gils and Wiersma 1996 | 39 | van Gils and Wiersma 1996 |
| 27 | b | *Limosa limosa* | Charadriiformes | 4 | van Gils and Wiersma 1996 | n/a |  | 336 | van Gils and Wiersma 1996 | 40 | van Gils and Wiersma 1996 |
| 27 | b | *Numenius arquata* | Charadriiformes | 4 | van Gils and Wiersma 1996 | 78.5 | Rehfisch et al. 2003 | 813.8 | van Gils and Wiersma 1996 | 55 | van Gils and Wiersma 1996 |
| 27 | b | *Numenius madagascariensis* | Charadriiformes | 4 | van Gils and Wiersma 1996 | 393.88 | Finn et al. 2007 | 925 | van Gils and Wiersma 1996 | 59.5 | van Gils and Wiersma 1996 |
| 27 | b | *Numenius phaeopus* | Charadriiformes | 4 | van Gils and Wiersma 1996 | n/a |  | 433.3 | van Gils and Wiersma 1996 | 43 | van Gils and Wiersma 1996 |
| 27 | b | *Philomachus pugnax* | Charadriiformes | 4 | van Gils and Wiersma 1996 | n/a |  | 156 | van Gils and Wiersma 1996 | 25.8 | van Gils and Wiersma 1996 |
| 27 | b | *Pluvialis fulva* | Charadriiformes | 4 | Wiersma 1992 | 1962.5 | Johnson and Connors 2010 | 146 | Wiersma 1992 | 24.5 | Wiersma 1992 |
| 27 | b | *Pluvialis squatarola* | Charadriiformes | 4 | Wiersma 1992 | 314 | Rehfisch et al.1996 | 247 | Wiersma 1992 | 29 | Wiersma 1992 |
| 27 | b | *Recurvirostra avosetta* | Charadriiformes | 3.5 | Pierce 1992 | n/a |  | 311 | Pierce 1996 | 43.5 | Pierce 1996 |
| 27 | b | *Tringa erythropus* | Charadriiformes | 4 | van Gils and Wiersma 1996 | n/a |  | 163.5 | van Gils and Wiersma 1996 | 30.5 | van Gils and Wiersma 1996 |
| 27 | b | *Tringa glareola* | Charadriiformes | 4 | van Gils and Wiersma 1996 | n/a |  | 66 | van Gils and Wiersma 1996 | 21 | van Gils and Wiersma 1996 |
| 27 | b | *Tringa nebularia* | Charadriiformes | 4 | van Gils and Wiersma 1996 | n/a |  | 207.5 | van Gils and Wiersma 1996 | 32.5 | van Gils and Wiersma 1996 |
| 27 | b | *Tringa stagnatilis* | Charadriiformes | 4 | van Gils and Wiersma 1996 | n/a |  | 81.5 | van Gils and Wiersma 1996 | 24 | van Gils and Wiersma 1996 |
| 27 | b | *Tringa totanus* | Charadriiformes | 4 | van Gils and Wiersma 1996 | 1808.6 | Rehfisch et al. 2003 | 120 | van Gils and Wiersma 1996 | 28 | van Gils and Wiersma 1996 |
| 27 | b | *Xenus cinereus* | Charadriiformes | 4 | van Gils and Wiersma 1996 | n/a |  | 88 | van Gils and Wiersma 1996 | 23.5 | van Gils and Wiersma 1996 |
| 28 | b | *Calidris alpina* | Charadriiformes | 4 | Warnock and Gill 1996 | 35600 | Taft et al. 2008 | 56 | Warnock and Gill 1996 | 19 | Warnock and Gill 1996 |
| 29 | b | *Ardeola ralloides* | Ciconiiformes | 4.5 | Hafner et al. 2001 | 1962.5 | Hafner et al. 2001 | 300 | Martínez-Vilalta and Motis 1992 | 44.5 | Martínez-Vilalta and Motis 1992 |
| 29 | b | *Bubulcus ibis* | Ciconiiformes | 4.3 | Prosper and Hafner 1996 | 122.66 | Telfair 2006 | 365 | Martínez-Vilalta and Motis 1992 | 51 | Martínez-Vilalta and Motis 1992 |
| 29 | b | *Egretta garzetta* | Ciconiiformes | 4.1 | Bennetts et al. 2000 | 314 | Hafner and Britton 1983 | 459 | Martínez-Vilalta and Motis 1992 | 60 | Martínez-Vilalta and Motis 1992 |
| 29 | b | *Nycticorax nycticorax* | Ciconiiformes | 4 | Martínez-Vilalta and Motis 1992 | 21.022 | Wong et al. 1999 | 662.5 | Martínez-Vilalta and Motis 1992 | 60.5 | Martínez-Vilalta and Motis 1992 |
| 30 | b | *Agelaius phoeniceus* | Passeriformes | 5.6 | Yasukawa and Searcy 1995 | 0.15 | Yasukawa and Searcy 1995 | 57.2 | Yasukawa and Searcy 1995 | 22 | Rosenthal 2004 |
| 30 | b | *Cistothorus palustris* | Passeriformes | 5 | Kroodsma and Verner 1997 | 0.1 | Leonard and Picman 1986, Verner 1965 | 11.5 | Kroodsma and Verner 1997 | 122 | Kroodsma and Verner 1997 |
| 30 | b | *Gallinula galeata* | Gruiformes | 8 | Brackney et al. 1982 | 1.22 | Matthews 1983 | 383 | Bannor and Kiviat 2002 | 33.5 | Bannor and Kiviat 2002 |
| 30 | b | *Geothlypis trichas* | Passeriformes | 8 | Abroe et al. 2007 | 1.2 | Guzy and Ritchison 1999 | 9.5 | Guzy and Ritchison 1999 | 12 | Guzy and Ritchison 1999 |
| 30 | b | *Ixobrychus exilis* | Ciconiiformes | 3.8 | Poole et al. 2009 | 9.7 | Bogner and Baldassarre 2002 | 80 | Poole et al. 2009 | 32 | Poole et al. 2009 |
| 30 | b | *Melospiza georgiana* | Passeriformes | 3.8 | Mowbray 1997 | 0.17 | Mowbray 1997 | 17.8 | Mowbray 1997 | 13.5 | Mowbray 1997 |
| 30 | b | *Porzana carolina* | Gruiformes | 11.5 | Lor and Malecki 2006 | 0.19 | Johnson and Dinsmore 1985 | 80 | Melvin and Gibbs 2012 | 22.5 | Melvin and Gibbs 2012 |
| 30 | b | *Rallus limicola* | Gruiformes | 7.9 | Lor and Malecki 2006 | 0.18 | Johnson and Dinsmore 1985 | 87 | Conway 1995 | 24.5 | Conway 1995 |
| 31 | b | *Anas wyvilliana* | Anseriformes | 8.3 | Engilis et al. 2002 | n/a |  | 532 | Engilis et al. 2002 | 31.5 | Carboneras 1992b |
| 32 | b | *Gallinula galeata* | Gruiformes | 6.7 | Helm et al. 1987 | 1.22 | Matthews 1983 | 383 | Bannor and Kiviat 2002 | 33.5 | Bannor and Kiviat 2002 |
| 32 | b | *Ixobrychus exilis* | Ciconiiformes | 3.8 | Poole et al. 2009 | 223.2 | Griffin et al. 2009 | 80 | Poole et al. 2009 | 32 | Poole et al. 2009 |
| 32 | b | *Porphyrio martinica* | Gruiformes | 5.2 | West and Hess 2002 | 1.03 | West and Hess 2002 | 248 | West and Hess 2002 | 31.5 | West and Hess 2002 |
| 33 | b | *Agelaius phoeniceus* | Passeriformes | 5.6 | Yasukawa and Searcy 1995 | 0.16 | Yasukawa and Searcy 1995 | 57.2 | Yasukawa and Searcy 1995 | 22 | Rosenthal 2004 |
| 33 | b | *Anas discors* | Anseriformes | 10.1 | Rohwer et al. 2002 | 0.69 | Stewart and Titman 1980 | 431 | Rohwer et al. 2002 | 38.5 | Mingo 2008 |
| 33 | b | *Anas platyrhynchos* | Anseriformes | 8.7 | Drilling et al. 2002 | 225 | Gilmer et al. 1975 | 1150 | Drilling et al. 2002 | 57.5 | Drilling et al. 2002 |
| 33 | b | *Chlidonias niger* | Charadriiformes | 2.8 | Maxson et al. 2007 | 452.16 | Chapman Mosher 1986 | 55 | Heath et al. 2009 | 24.5 | Heath et al. 2009 |
| 33 | b | *Cistothorus palustris* | Passeriformes | 5 | Kroodsma and Verner 1997 | 0.1 | Leonard and Picman 1986, Verner 1965 | 11.5 | Kroodsma and Verner 1997 | 122 | Kroodsma and Verner 1997 |
| 33 | b | *Cistothorus platensis* | Passeriformes | 7 | Herkert et al. 2001 | 0.178 | Herkert et al. 2001 | 8.5 | Herkert et al. 2001 | 11 | Herkert et al. 2001 |
| 33 | b | *Empidonax alnorum* | Passeriformes | 3.7 | Lowther 1999 | 2.15 | Lowther 1999 | 13 | Lowther 1999 | 15 | Lowther 1999 |
| 33 | b | *Fulica americana* | Gruiformes | 8.5 | Brisbin et al. 2002 | 0.15 | Brisbin et al. 2002 | 637.5 | Brisbin et al. 2002 | 37.5 | Brisbin et al. 2002 |
| 33 | b | *Gallinago delicata* | Charadriiformes | 3.9 | Mueller 1999 | 235 | Cline and Haig 2011 | 100 | Mueller 1999 | 28 | Mueller 1999 |
| 33 | b | *Geothlypis trichas* | Passeriformes | 3.5 | Abroe et al. 2007 | 0.38 | Guzy and Ritchison 1999 | 9.5 | Guzy and Ritchison 1999 | 12 | Guzy and Ritchison 1999 |
| 33 | b | *Grus canadensis* | Gruiformes | 2 | Tacha et al. 1992 | 85.1 | Tacha et al. 1992 | 3545 | Tacha et al. 1992 | 120 | Tacha et al. 1992 |
| 33 | b | *Melospiza georgiana* | Passeriformes | 3.8 | Mowbray 1997 | 0.17 | Mowbray 1997 | 17.8 | Mowbray 1997 | 13.5 | Mowbray 1997 |
| 33 | b | *Podilymbus podiceps* | Podicipediformes |  |  |  |  |  |  |  |  |
| 33 | b | *Porzana carolina* | Gruiformes | 10.2 | Melvin and Gibbs 2012 | 0.19 | Johnson and Dinsmore 1985 | 80 | Melvin and Gibbs 2012 | 22.5 | Melvin and Gibbs 2012 |
| 33 | b | *Rallus limicola* | Gruiformes | 7.5 | Kaufmann 1989 | 0.18 | Johnson and Dinsmore 1985 | 87 | Conway 1995 | 24.5 | Conway 1995 |
| 33 | b | *Setophaga petechia* | Passeriformes | 4.1 | Lowther et al. 1999 | 0.284 | Lowther et al. 1999 | 10 | Lowther et al. 1999 | 12.5 | Lowther et al. 1999 |
| 33 | b | *Xanthocephalus xanthocephalus* | Passeriformes | 3.8 | Twedt and Crawford 1995 | 78.5 | Twedt and Crawford 1995 | 80 | Twedt and Crawford 1995 | 240 | Twedt and Crawford 1995 |
| 24 | r | *Emydoidea blandingii* | Testudine | 8 | MacCulloch and Weller 1988 | 12 | Millar and Blouin-Demers 2011 | 1601 | Millar 2010 | 22.4 | Millar 2010 |
| 24 | r | *Sternotherus odoratus* | Testudine | 4.7 | COSEWIC 2002 | 6.2 | Carrière 2007 | 173.5 | Edmonds 1999 | 10.1 | Edmonds 1999 |
| 34 | r | *Chrysemys picta marginata* | Testudine | 12.4 | Rowe et al. 2003 | 1.2 | Rowe 2003 | 354.4 | Rowe 2003 | 14.4 | Rowe 2003 |
| 34 | r | *Emydoidea blandingii* | Testudine | 10.2 | Congdon and van Loben Sels 1993 | 48.99 | Congdon et al. 2011 | 1200 | Congdon and van Loben Sels 1993 | 19.5 | Congdon and van Loben Sels 1993 |
| 34 | r | *Nerodia erythrogaster neglecta* | Squamata | 17.7 | Gibbons and Dorcas 2004 | 15.8 | Roe et al. 2004 | 401 | Gibbons and Dorcas 2004 | 99 | Conant and Collins 1991 |
| 34 | r | *Nerodia sipedon sipedon* | Squamata | 27.2 | Ernst and Ernst 2003 | 4 | Roe et al. 2004 | 165 | Roe et al. 2004 | 83.9 | Conant and Collins 1991 |
| 35 | r | *Chrysemys picta* | Testudine | 20 | Christiansen and Moll 1973 | 1.2 | Rowe 2003 | 354.4 | Rowe 2003 | 14.4 | Rowe 2003 |
| 36 | r | *Chrysemys picta marginata* | Testudine | 14.4 | Whillans and Crossman 1977; Ernst and Lovich 2009 | 1.2 | Rowe 2003 | 500 | Brooks et al 2003 | 15 | MacCulloch 2002 |
| 37 | r | *Clemmys guttata* | Testudine | 5.3 | Joyal 1996 | 7.6 | Joyal et al. 2001 | 183 | Litzgus and Mousseau 2006 | 11.2 | Litzgus and Brooks 1998 |
| 37 | r | *Emydoidea blandingii* | Testudine | 8.5 | Joyal et al. 2000 | 36.3 | Joyal et al. 2001 | 1342.5 | Joyal et al. 2000 | 20.9 | Joyal et al. 2000 |
| 38 | r | *Chrysemys picta* | Testudine | 14.4 | Whillans and Crossman 1977; Ernst and Lovich 2009 | 1.2 | Rowe 2003 | 392.5 | Steen and Gibbs 2004 | 13.3 | Gibbs et al. 2007 |
| 39 | r | *Nerodia erythrogaster neglecta* | Squamata | 17.7 | Gibbons and Dorcas 2004 | 26.5 | Gibbons and Dorcas 2004 | 401 | Gibbons and Dorcas 2004 | 99 | Conant and Collins 1991 |
| 39 | r | *Nerodia rhombifer rhombifer* | Squamata | 23.1 | Plummer 1992 | 3.5 | Gibbons and Dorcas 2004 | 553.4 | Plummer 1992 | 99 | Conant and Collins 1991 |
| 39 | r | *Nerodia sipedon pleuralis* | Squamata | 27.2 | Ernst and Ernst 2003 | 2.82 | Roth and Greene 2006 | n/a |  | 79 | Conant and Collins 1991 |
| 40 | r | *Glyptemys muhlenbergii* | Testudine | 3.5 | Whitlock 2002 | 0.55 | Whitlock 2002 | 114.7 | Whitlock 2002 | 8.3 | Gibbs et al. 2007 |
| 41 | r | *Apalone spinifera spinifera* | Testudine | 35.8 | Ernst and Lovich 2009; Robinson and Murphy 1978 | 1740.5 | Galois et al. 2002 | n/a |  | 25.6 | Gibbs et al. 2007 |
| 41 | r | *Chelydra serpentina serpentina* | Testudine | 35.7 | Iverson et al. 1997 | 11.13 | Paisley et al. 2009 | 5600 | Paisley et al. 2009 | 29.9 | Paisley et al. 2009 |
| 41 | r | *Chrysemys picta marginata* | Testudine | 12.4 | Rowe et al. 2003 | 32.45 | Tran et al. 2007 | 354.4 | Rowe 2003 | 14.4 | Rowe 2003 |
| 41 | r | *Trachemys scripta elegans* | Testudine | 36.6 | Tucker et al. 1998; Tucker 2001 | 327.6 | Jaeger and Cobb 2012 | 1719.7 | Tucker et al. 1998; Tucker 2001 | 19.3 | Gibbons and Lovich 1990 |
| 42 | r | *Chelodina longicollis* | Testudine | 8.4 | Kennett and Georges 1990; Arthur Georges, personal communication | 11.5 | Roe and Georges 2008 | 602 | Jarrett 2011 | 25 | Jarrett 2011 |
| 43 | r | *Chelydra serpentina* | Testudine | 30.9 | Petokas and Alexander 1980. | 5.98 | Pettit et al. 1995 | 6005 | Steen and Gibbs 2004 | 28.3 | Gibbs et al. 2007 |
| 43 | r | *Chrysemys picta* | Testudine | 14.4 | Whillans and Crossman 1977 | 1.2 | Rowe 2003 | 392.5 | Steen and Gibbs 2004 | 13.3 | Gibbs et al. 2007 |
| 44 | r | *Chelydra serpentina* | Testudine | 30.9 | Petokas and Alexander 1980 | 5.98 | Pettit et al. 1995 | 6005 | Steen and Gibbs 2004 | 28.3 | Gibbs et al. 2007 |
| 44 | r | *Chrysemys picta* | Testudine | 14.4 | Whillans and Crossman 1977; Ernst and Lovich 2009 | 1.2 | Rowe 2003 | 392.5 | Steen and Gibbs 2004 | 13.3 | Gibbs et al. 2007 |
| 1 | a | *Bufo boreas* | Anura | 12000 | Samollow 1980, Vonesh and Cruz 2002 | 41.775 | Browne and Paszkowski 2010 | n/a |  | 9 | Russell et al. 2000 |
| 1 | a | *Pseudacris maculata* | Anura | 325 | Whiting 2010 | 7.292 | Spencer 1964 | n/a |  | 3 | Russell et al. 2000 |
| 1 | a | *Rana sylvatica* | Anura | 876 | Corn and Livo 1989 | 2.92 | Rittenhouse and Semlitsch 2009 | n/a |  | 4 | Russell et al. 2000 |
| 45 | a | *Ambystoma maculatum* | Caudata | 268.8 | Karraker 2007 | 1.093 | Semlitsch and Bodie 2003 | n/a |  | 20 | Petranka 2010 |
| 45 | a | *Rana sylvatica* | Anura | 690 | Karraker and Gibbs 2009 | 2.9089 | Baldwin et al. 2006 |  |  | 5.25 | Gibbs et al. 2007 |
| 46 | a | *Bufo bufo* | Anura | 3100 | Cooper et al. 2008 | 54.73 | Sinsch 1988 | n/a |  | 11.5 | Cooper et al. 2008 |
| 46 | a | *Hyla arborea* | Anura | 1100 | Kuzmin 2013b | 7.864 | Pellet et al. 2006 | n/a |  | 4.3 | Cooper et al. 2008 |
| 46 | a | *Ichthyosaura alpestris* | Caudata | 150 | Griffiths 1996 | 3.05 | Kovar et al. 2009 | n/a |  | 10 | Spellerberg 2002 |
| 46 | a | *Lissotriton vulgaris* | Caudata | 180 | Kuzmin 2013c | 2.8 | Kovar et al. 2009 | n/a |  | 9 | Spellerberg 2002 |
| 46 | a | *Rana esculenta* | Anura | 11000 | Cooper et al. 2008 | 1.766 | Holenweg Peter 2001 | n/a |  | 9 | Cooper et al. 2008 |
| 46 | a | *Rana lessonae* | Anura | 2420 | Kuzmin and Cavagnaro 2013 | 1.539 | Holenweg Peter 2001 | n/a |  | 6.8 | Cooper et al. 2008 |
| 46 | a | *Rana temporaria* | Anura | 2585 | Cooper et al 2008 | 9.397 | Kovar et al. 2009 | n/a |  | 10 | Cooper et al. 2008 |
| 46 | a | *Triturus cristatus* | Caudata | 200 | Griffiths 1996 | 20.82 | Jehle and Arntzen 2000, Sinsch 2007 | 7.4 | Jehle and Arntzen 2000 | 13 | Spellerberg 2002 |
| 47 | a | *Hemidactylium scutatum* | Caudata | 47 | Petranka 2010 | 3.54 | Richmond 1999 | n/a |  | 7.5 | Petranka 2010 |
| 48 | a | *Ambystoma tigrinum tigrinum* | Caudata | 421 | Petranka 2010 | 0.287 | Madison and Farrand 1998 | 30.3 | Madison and Farrand 1998 | 34 | Petranka 2010 |
| 49 | a | *Ambystoma maculatum* | Caudata | 268.8 | Karraker 2007 | 1.093 | Semlitsch and Bodie 2003 | n/a |  | 20 | Petranka 2010 |
| 49 | a | *Notophthalmus viridescens* | Caudata | 304 | Petranka 2010 | 0.045 | Healy 1975 | n/a |  | 8.9 | Petranka 2010 |
| 49 | a | *Pseudacris crucifer* | Anura | 900 | Gibbs et al. 2007 | 5.89 | Delzell 1958 | n/a |  | 2.5 | Conant and Collins 1991; Gibbs et al. 2007 |
| 49 | a | *Rana catesbeiana* | Anura | 16000 | Gibbs et al. 2007 | 12.94 | Semlitsch and Bodie 2003 | n/a |  | 12 | Conant and Collins 1991; Gibbs et al. 2007 |
| 49 | a | *Rana clamitans* | Anura | 3250 | Gibbs et al. 2007 | 3.83 | Lamoureux et al 2002 | n/a |  | 7.3 | Gibbs et al. 2007 |
| 49 | a | *Rana palustris* | Anura | 2500 | Gibbs et al. 2007 | n/a |  | n/a |  | 6 | Gibbs et al. 2007 |
| 49 | a | *Rana sylvatica* | Anura | 690 | Karraker and Gibbs 2009 | 2.909 | Baldwin et al 2006 | n/a |  | 5.3 | Conant and Collins 1991; Gibbs et al. 2007 |
| 50 | a | *Pseudacris regilla* | Anura | 325 | Whiting 2010 | 7.292 | Spencer 1964 | n/a |  | 2.8 | Conant and Collins 1991 |
| 50 | a | *Rana catesbeiana* | Anura | 16000 | Gibbs et al. 2007 | 12.94 | Semlitsch and Bodie 2003 | n/a |  | 12 | Conant and Collins 1991; Gibbs et al. 2007 |
| 50 | a | *Rana draytonii* | Anura | 2000 | Fellers 2013 | 94.985 | Bulger et al. 2003 | 131 | Bulger et al. 2003 | 10.5 | Bulger et al. 2003 |
| 51 | a | *Ichthyosaura alpestris* | Caudata | 150 | Griffiths 1996 | 3.05 | Kovar et al. 2009 | n/a |  | 10 | Spellerberg 2002 |
| 51 | a | *Lissotriton vulgaris* | Caudata | 180 | Kuzmin 2013c | 2.8 | Kovar et al. 2009 | n/a |  | 9 | Spellerberg 2002 |
| 52 | a | *Lissotriton helveticus* | Caudata | 365 | van der Meijden and Cavagnaro 2013 | 1.766 | Diego-Rasilla and Luengo 2007 | n/a |  | 6.5 | Spellerberg 2002 |
| 53 | a | *Bufo americanus* | Anura | 8000 | Gibbs et al. 2007 | 34.1 | Forester et al. 2006 | n/a |  | 8.5 | Fisher et al. 2007 |
| 53 | a | *Hyla versicolor* | Anura | 2000 | Gibbs et al. 2007 | 9.075 | Johnson et al 2007 | n/a |  | 4 | Conant and Collins 1991; Gibbs et al. 2007 |
| 53 | a | *Pseudacris crucifer* | Anura | 900 | Fisher et al. 2007 | 5.89 | Delzell 1958 | n/a |  | 2.5 | Conant and Collins 1991; Gibbs et al. 2007 |
| 53 | a | *Rana clamitans* | Anura | 3250 | Gibbs et al. 2007 | 1.47 | Oldham 1967 | n/a |  | 8.5 | Fisher et al. 2007 |
| 53 | a | *Rana pipiens* | Anura | 3000 | Gibbs et al. 2007; Gilbert et al. 1994 | 135.95 | Blomquist and Hunter 2009 | n/a |  | 7 | Fisher et al. 2007 |
| 53 | a | *Rana sylvatica* | Anura | 690 | Karraker and Gibbs 2009 | 2.909 | Baldwin et al 2006 | n/a |  | 5.3 | Conant and Collins 1991; Gibbs et al. 2007 |
| 54 | a | *Ambystoma macrodactylum* | Caudata | 121 | Howard and Wallace 1985 | 0.02 | Pilliod and Fronzuto 2013 | n/a |  | 13.5 | Petranka 2010 |
| 54 | a | *Pseudacris regilla* | Anura | 575 | Rorabaugh and Lannoo 2013 | 3.97 | Rorabaugh and Lannoo 2013 | n/a |  | 3.7 | Rorabaugh and Lannoo 2013 |
| 54 | a | *Rana luteiventris* | Anura | 600 | Reaser and Pilliod 2013 | 7.3 | Pilliod et al. 2002 | n/a |  | 7.3 | Russell et al. 2000 |
| 55 | a | *Ambystoma tigrinum mavortium* | Caudata | 2385 | Rose and Armentrout 1976 | 0.923 | Steen et al. 2006 | 34.3 | Gray 2002 | 34 | Petranka 2010 |
| 55 | a | *Bufo cognatus* | Anura | 11074 | Krupa 1994 | 28.26 | Graves and Krupa 2013 | 54 | Gray 2002 | 6.9 | Conant and Collins 1991 |
| 55 | a | *Spea bombifrons* | Anura | 1600 | Woodward 1987 | 25.95 | Landreth and Christensen 1971 | 11.2 | Gray 2002 | 4.5 | Conant and Collins 1991 |
| 55 | a | *Spea multiplicata* | Anura | 1062 | Pfenning and Pfenning 2005, Woodward 1987 | n/a |  | 8.7 | Gray 2002 | 4.5 | Conant and Collins 1991 |
| 56 | a | *Ambystoma laterale* | Caudata | 206 | Petranka 2010 | 1.469 | Faccio 2003 | n/a |  | 10.3 | Petranka 2010 |
| 56 | a | *Ambystoma maculatum* | Caudata | 268.8 | Karraker 2007 | 1.093 | Semlitsch and Bodie 2003 | n/a |  | 20 | Petranka 2010 |
| 56 | a | *Bufo americanus* | Anura | 8000 | Gibbs et al. 2007 | 34.1 | Forester et al. 2006 | n/a |  | 7 | Conant and Collins 1991; Gibbs et al. 2007 |
| 56 | a | *Notopthalmus viridescens* | Caudata | 304 | Petranka 2010 | 0.045 | Healy 1975 | n/a |  | 8.9 | Petranka 2010 |
| 56 | a | *Rana clamitans* | Anura | 3250 | Gibbs et al. 2007 | 3.83 | Lamoureux et al 2002 | n/a |  | 7.3 | Gibbs et al. 2007 |
| 56 | a | *Rana pipiens* | Anura | 3000 | Gibbs et al. 2007; Gilbert et al. 1994 | 135.95 | Blomquist and Hunter 2009 | n/a |  | 7 | Conant and Collins 1991; Gibbs et al. 2007 |
| 56 | a | *Rana septentrionalis* | Anura | 1250 | Shirose and Brooks 1995 | n/a |  | n/a |  | 6.3 | Gibbs et al. 2007 |
| 56 | a | *Rana sylvatica* | Anura | 690 | Karraker and Gibbs 2009 | 2.909 | Baldwin et al 2006 | n/a |  | 5.3 | Conant and Collins 1991; Gibbs et al. 2007 |
| 57 | a | *Triturus cristatus* | Caudata | 200 | Griffiths 1996 | 20.82 | Jehle and Arntzen 2000, Sinsch 2007 | 7.4 | Jehle and Arntzen 2000 | 13 | Spellerberg 2002 |
| 58 | a | *Crinia signifera* | Anura | 125 | Hero et al. 2005 | 0.502 | Westgate et al. 2012 | n/a |  | 2.5 | Hero et al. 2005 |
| 58 | a | *Limnodynastes dumerilii* | Anura | 3900 | Hero 2013 | 1.96 | Carthew et al. 2009 | n/a |  | 6.9 | Tyler and Knight 2009 |
| 58 | a | *Limnodynastes peronii* | Anura | 857 | Hero et al. 2005 | 0.159 | Martin Westgate, personal communication | n/a |  | 5.9 | Tyler and Knight 2009 |
| 58 | a | *Litoria ewingii/verreauxii* | Anura | 675 | Hero and Bishop 2013, Hero et al. 2013, Turner 2004 | n/a |  | n/a |  | 3.4 | Tyler and Knight 2009 |
| 58 | a | *Paracrinia haswelli* | Anura | 188.3 | Anstis 2007 | 0.502 | Westgate et al. 2012 | n/a |  | 3.3 | Tyler and Knight 2009 |
| 59 | a | *Crinia signifera* | Anura | 125 | Hero et al. 2005 | 0.502 | Westgate et al. 2012 | n/a |  | 2.5 | Hero et al. 2005 |
| 59 | a | *Limnodynastes dumerilii* | Anura | 3900 | Hero 2013 | 1.96 | Carthew et al. 2009 | n/a |  | 6.9 | Tyler and Knight 2009 |
| 59 | a | *Limnodynastes peronii* | Anura | 857 | Hero et al. 2005 | 0.159 | Martin Westgate, personal communication | n/a |  | 5.9 | Tyler and Knight 2009 |
| 59 | a | *Limnodynastes tasmaniensis* | Anura | 1000 | Wilson et al. 2012 | n/a |  | n/a |  | 3.8 | Tyler and Knight 2009 |
| 59 | a | *Litoria ewingii* | Anura | 600 | Hero and Bishop 2013 | n/a |  | n/a |  | 3.5 | Tyler and Knight 2009 |
| 59 | a | *Litoria fallax* | Anura | 250 | Turner 2004 | n/a |  | n/a |  | 2.6 | Tyler and Knight 2009 |
| 59 | a | *Litoria raniformis* | Anura | 3387 | Heard et al. 2012a | 3.14 | Heard et al. 2012b | n/a |  | 7.1 | Tyler and Knight 2009 |
| 59 | a | *Litoria verreauxii* | Anura | 750 | Hero et al. 2013, Turner 2004 | n/a |  | n/a |  | 3.3 | Tyler and Knight 2009 |
| 60 | a | *Bombina variegata* | Anura | 72.5 | Kuzmin 2013a | 1.766 | Beshkov and Jameson 1980, Hartel 2008 | n/a |  | 4.1 | Spellerberg 2002 |
| 60 | a | *Bufo bufo* | Anura | 3100 | Cooper et al. 2008 | 54.73 | Sinsch 1988 | n/a |  | 11.5 | Cooper et al. 2008 |
| 60 | a | *Hyla arborea* | Anura | 1100 | Kuzmin 2013b | 7.864 | Pellet et al. 2006 | n/a |  | 4.3 | Cooper et al. 2008 |
| 60 | a | *Lissotriton vulgaris* | Caudata | 180 | Kuzmin 2013c | 2.8 | Kovar et al. 2009 | n/a |  | 9 | Spellerberg 2002 |
| 60 | a | *Pelobates fuscus* | Anura | 1740 | Kuzmin and Andreone 2013 | 4.906 | Hels 2002 | n/a |  | 7.3 | Spellerberg 2002 |
| 60 | a | *Rana dalmatina* | Anura | 910 | Ponsero and Joly 1998 | 0.93 | Ponsero and Joly 1998 | n/a |  | 7.5 | Cooper et al. 2008 |
| 60 | a | *Rana esculenta* | Anura | 11000 | Cooper et al. 2008 | 1.766 | Holenweg Peter 2001 | n/a |  | 9 | Cooper et al. 2008 |
| 60 | a | *Rana temporaria* | Anura | 2585 | Cooper et al 2008 | 9.397 | Kovar et al. 2009 | n/a |  | 10 | Cooper et al. 2008 |
| 60 | a | *Triturus cristatus* | Caudata | 200 | Griffiths 1996 | 20.82 | Jehle and Arntzen 2000, Sinsch 2007 | 7.4 | Jehle and Arntzen 2000 | 13 | Spellerberg 2002 |
| 61 | a | *Litoria raniformis* | Anura | 3387 | Heard et al. 2012a | 3.14 | Heard et al. 2012b | n/a |  | 7.1 | Tyler and Knight 2009 |
| 62 | a | *Bufo americanus* | Anura | 8000 | Gibbs et al. 2007 | 34.1 | Forester et al. 2006 | n/a |  | 7 | Conant and Collins 1991; Gibbs et al. 2007 |
| 62 | a | *Hyla versicolor* | Anura | 2000 | Gibbs et al. 2007 | 9.075 | Johnson et al 2007 | n/a |  | 4 | Conant and Collins 1991; Gibbs et al. 2007 |
| 62 | a | *Notophthalmus viridescens* | Caudata | 304 | Petranka 2010 | 0.045 | Healy 1975 | n/a |  | 8.9 | Petranka 2010 |
| 62 | a | *Pseudacris crucifer* | Anura | 900 | Fisher et al. 2007 | 5.89 | Delzell 1958 | n/a |  | 2.5 | Conant and Collins 1991; Gibbs et al. 2007 |
| 62 | a | *Pseudacris triseriata* | Anura | 1000 | Gibbs et al. 2007, Cooper et al. 2008 | 0.785 | Kramer 1973 | n/a |  | 3 | Conant and Collins 1991; Gibbs et al. 2007 |
| 62 | a | *Rana clamitans* | Anura | 3250 | Gibbs et al. 2007 | 1.47 | Oldham 1967 | n/a |  | 8.5 | Fisher et al. 2007 |
| 62 | a | *Rana pipiens* | Anura | 3000 | Gibbs et al. 2007; Gilbert et al. 1994 | 135.95 | Blomquist and Hunter 2009 | n/a |  | 7 | Fisher et al. 2007 |
| 62 | a | *Rana sylvatica* | Anura | 690 | Karraker and Gibbs 2009 | 2.909 | Baldwin et al 2006 | n/a |  | 5.3 | Conant and Collins 1991; Gibbs et al. 2007 |
| 63 | a | *Ambystoma maculatum* | Caudata | 224 | Petranka 2010 | 1.093 | Semlitsch and Bodie 2003 | n/a |  | 20 | Petranka 2010 |
| 63 | a | *Rana sylvatica* | Anura | 690 | Karraker and Gibbs 2009 | 2.909 | Baldwin et al 2006 | n/a |  | 5.3 | Conant and Collins 1991; Gibbs et al. 2007 |
| 64 | a | *Ambystoma laterale* | Caudata | 206 | Petranka 2010 | 1.469 | Faccio 2003 | n/a |  | 10.3 | Petranka 2010 |
| 64 | a | *Ambystoma maculatum* | Caudata | 268.8 | Karraker 2007 | 1.093 | Semlitsch and Bodie 2003 | n/a |  | 20 | Petranka 2010 |
| 64 | a | *Bufo americanus* | Anura | 8000 | Gibbs et al. 2007 | 34.1 | Forester et al. 2006 | n/a |  | 8.5 | Fisher et al. 2007 |
| 64 | a | *Hyla versicolor* | Anura | 2000 | Gibbs et al. 2007 | 9.075 | Johnson et al 2007 | n/a |  | 4 | Conant and Collins 1991; Gibbs et al. 2007 |
| 64 | a | *Notophthalmus viridescens* | Caudata | 304 | Petranka 2010 | 0.045 | Healy 1975 | n/a |  | 8.9 | Petranka 2010 |
| 64 | a | *Pseudacris crucifer* | Anura | 900 | Fisher et al. 2007 | 5.89 | Delzell 1958 | n/a |  | 2.5 | Conant and Collins 1991; Gibbs et al. 2007 |
| 64 | a | *Pseudacris triseriata* | Anura | 1000 | Gibbs et al. 2007, Cooper et al. 2008 | 0.785 | Kramer 1973 | n/a |  | 3 | Conant and Collins 1991; Gibbs et al. 2007 |
| 64 | a | *Rana catesbeiana* | Anura | 13000 | Howard 1978 | 12.94 | Semlitsch and Bodie 2003 | n/a |  | 12 | Conant and Collins 1991; Gibbs et al. 2007 |
| 64 | a | *Rana clamitans* | Anura | 3250 | Gibbs et al. 2007 | 1.47 | Oldham 1967 | n/a |  | 8.5 | Fisher et al. 2007 |
| 64 | a | *Rana pipiens* | Anura | 3000 | Gibbs et al. 2007; Gilbert et al. 1994 | 135.95 | Blomquist and Hunter 2009 | n/a |  | 7 | Fisher et al. 2007 |
| 64 | a | *Rana septentrionalis* | Anura | 1250 | Shirose and Brooks 1995 | n/a |  | n/a |  | 6.5 | Fisher et al. 2007 |
| 64 | a | *Rana sylvatica* | Anura | 690 | Karraker and Gibbs 2009 | 2.909 | Baldwin et al 2006 | n/a |  | 5.3 | Conant and Collins 1991; Gibbs et al. 2007 |
| 65 | a | *Ambystoma laterale/A. maculatum* | Caudata | 259.4 | Karraker 2007, Petranka 2010 | 1.28 | Faccio 2003, Semlitsch and Bodie 2003 | n/a |  | 15.2 | Petranka 2010 |
| 65 | a | *Bufo americanus* | Anura | 8000 | Gibbs et al. 2007 | 34.1 | Forester et al. 2006 | n/a |  | 8.5 | Fisher et al. 2007 |
| 65 | a | *Notopthalmus viridescens* | Caudata | 304 | Petranka 2010 | 0.045 | Healy 1975 | n/a |  | 8.9 | Petranka 2010 |
| 65 | a | *Pseudacris crucifer* | Anura | 900 | Fisher et al. 2007 | 5.89 | Delzell 1958 | n/a |  | 2.5 | Conant and Collins 1991; Gibbs et al. 2007 |
| 65 | a | *Rana catesbeiana* | Anura | 13000 | Howard 1978 | 12.94 | Semlitsch and Bodie 2003 | n/a |  | 12 | Conant and Collins 1991; Gibbs et al. 2007 |
| 65 | a | *Rana clamitans* | Anura | 3250 | Gibbs et al. 2007 | 1.47 | Oldham 1967 | n/a |  | 8.5 | Fisher et al. 2007 |
| 65 | a | *Rana palustris* | Anura | 2500 | Gibbs et al. 2007 | n/a |  | n/a |  | 6.5 | Fisher et al. 2007 |
| 65 | a | *Rana septentrionalis* | Anura | 1250 | Shirose and Brooks 1995 | n/a |  | n/a |  | 6.5 | Fisher et al. 2007 |
| 65 | a | *Rana sylvatica* | Anura | 690 | Karraker and Gibbs 2009 | 2.909 | Baldwin et al 2006 | n/a |  | 5.3 | Conant and Collins 1991; Gibbs et al. 2007 |
| 66 | a | *Rana pipiens* | Anura | 3045 | Corn and Livo 1989 | 135.95 | Blomquist and Hunter 2009 | n/a |  | 7 | Conant and Collins 1991; Gibbs et al. 2007 |
| 67 | a | *Ichthyosaura alpestris* | Caudata | 150 | Griffiths 1996 | 3.05 | Kovar et al. 2009 | n/a |  | 10 | Spellerberg 2002 |
| 67 | a | *Lissotriton helveticus* | Caudata | 365 | van der Meijden and Cavagnaro 2013 | 1.766 | Diego-Rasilla and Luengo 2007 | n/a |  | 6.5 | Spellerberg 2002 |
| 67 | a | *Triturus cristatus* | Caudata | 200 | Griffiths 1996 | 20.82 | Jehle and Arntzen 2000, Sinsch 2007 | 7.4 | Jehle and Arntzen 2000 | 13 | Spellerberg 2002 |
| 68 | a | *Rana muscosa* | Anura | 233 | Vredenburg et al. 2013 | 0.53 | Matthews and Pope 1999 | 26.9 | Matthews and Miaud 2007 | 6 | Matthews and Miaud 2007 |
| 69 | a | *Ambystoma tigrinum* | Caudata | 421 | Petranka 2010 | 0.287 | Madison and Farrand 1998 | 30.3 | Madison and Farrand 1998 | 34 | Petranka 2010 |
| 69 | a | *Bufo americanus* | Anura | 8000 | Gibbs et al. 2008 | 34.1 | Forester et al. 2006 | n/a |  | 7 | Conant and Collins 1991; Gibbs et al. 2007 |
| 69 | a | *Hyla versicolor* | Anura | 2000 | Gibbs et al. 2007 | 9.075 | Johnson et al 2007 | n/a |  | 4 | Conant and Collins 1991; Gibbs et al. 2007 |
| 69 | a | *Pseudacris crucifer* | Anura | 900 | Fisher et al. 2007 | 5.89 | Delzell 1958 | n/a |  | 2.5 | Conant and Collins 1991; Gibbs et al. 2007 |
| 69 | a | *Pseudacris triseriata* | Anura | 1000 | Gibbs et al. 2007, Cooper et al. 2008 | 0.785 | Kramer 1973 | n/a |  | 3 | Conant and Collins 1991; Gibbs et al. 2007 |
| 69 | a | *Rana clamitans* | Anura | 3250 | Gibbs et al. 2007 | 1.47 | Oldham 1967 | n/a |  | 8.5 | Fisher et al. 2007 |
| 69 | a | *Rana palustris* | Anura | 2500 | Gibbs et al. 2007 | n/a |  | n/a |  | 6 |  |
| 69 | a | *Rana pipiens* | Anura | 3000 | Gibbs et al. 2007 | 135.95 | Blomquist and Hunter 2009 | n/a |  | 7 | Conant and Collins 1991; Gibbs et al. 2007 |
| 70 | a | *Ambystoma tigrinum tigrinum* | Caudata | 421 | Petranka 2010 | 0.287 | Madison and Farrand 1998 | 30.3 | Madison and Farrand 1998 | 34 | Petranka 2010 |
| 70 | a | *Bufo americanus* | Anura | 8000 | Gibbs et al. 2008 | 34.1 | Forester et al. 2006 | n/a |  | 7 | Conant and Collins 1991; Gibbs et al. 2007 |
| 70 | a | *Bufo cognatus* | Anura | 11075 | Krupa 1995 | 28.26 | Graves and Krupa 2013 | 54 | Gray 2002 | 6.9 | Conant and Collins 1991 |
| 70 | a | *Hyla versicolor/H. chrysoscelis* | Anura | 1683 | Ritke et al. 1990, Jacqueline Doyle, personal communication | 9.075 | Johnson et al 2007 | n/a |  | 4 | Conant and Collins 1991 |
| 70 | a | *Pseudacris triseriata* | Anura | 1000 | Gibbs et al. 2007, Cooper et al. 2008 | 0.785 | Kramer 1973 | n/a |  | 3 | Conant and Collins 1991; Gibbs et al. 2007 |
| 70 | a | *Rana pipiens* | Anura | 3000 | Gibbs et al. 2007 | 135.95 | Blomquist and Hunter 2009 | n/a |  | 7 | Conant and Collins 1991; Gibbs et al. 2007 |
| 70 | a | *Rana sylvatica* | Anura | 650 | Berven 2009 | 2.909 | Baldwin et al 2006 | n/a |  | 5.3 | Conant and Collins 1991; Gibbs et al. 2007 |
| 71 | a | *Engystomops pustulosus* | Anura | 234.2 | Ryan 1983 | 2.005 | Marsh et al. 1999 | n/a |  | 2.8 | Marsh et al. 1999 |
| 72 | a | *Rana clamitans* | Anura | 3250 | Gibbs et al. 2007 | 1.47 | Oldham 1967 | n/a |  | 8.5 | Fisher et al. 2007 |
| 73 | a | *Bufo fowleri* | Anura | 3700 | Green 2012 | 0.64 | Boenke 2011 | n/a |  | 6.3 | Conant and Collins 1991; Gibbs et al. 2007 |
| 73 | a | *Hyla versicolor* | Anura | 1305 | Jacqueline Doyle, personal communication | 9.075 | Johnson et al 2007 | n/a |  | 4 | Conant and Collins 1991; Gibbs et al. 2007 |
| 73 | a | *Pseudacris crucifer* | Anura | 900 | Gibbs et al. 2007 | 5.89 | Delzell 1958 | n/a |  | 2.5 | Conant and Collins 1991; Gibbs et al. 2007 |
| 73 | a | *Rana catesbeiana* | Anura | 16000 | Gibbs et al. 2007 | 12.94 | Semlitsch and Bodie 2003 | n/a |  | 12 | Conant and Collins 1991; Gibbs et al. 2007 |
| 74 | a | *Rana virgatipes* | Anura | 400 | Mitchell 2012 | 0.816 | Given 1988 | n/a |  | 5.4 | Conant and Collins 1991 |
| 75 | a | *Hyla arborea* | Anura | 1100 | Kuzmin 2013b | 7.864 | Pellet et al. 2006 | n/a |  | 4.3 | Cooper et al. 2008 |
| 76 | a | *Hyla arborea* | Anura | 1100 | Kuzmin 2013b | 7.864 | Pellet et al. 2006 | n/a |  | 4.3 | Cooper et al. 2008 |
| 77 | a | *Acris crepitans* | Anura | 248 | Cooper et al. 2008 | 0.132 | Gray 1983, Lemckert 2004 | n/a |  | 2.5 | Conant and Collins 1991 |
| 77 | a | *Bufo americanus* | Anura | 6000 | Cooper et al. 2008 | 34.1 | Forester et al. 2006 | n/a |  | 7 | Conant and Collins 1991 |
| 77 | a | *Hyla chrysoscelis* | Anura | 2060 | Ritke et al. 1990 | n/a |  | 9.4 | Ritke et al. 1990 | 5 | Ritke et al. 1990 |
| 77 | a | *Hyla versicolor* | Anura | 1305 | Jacqueline Doyle, personal communication | 9.075 | Johnson et al 2007 | n/a |  | 4 | Conant and Collins 1991 |
| 77 | a | *Pseudacris triseriata* | Anura | 1000 | Cooper et al. 2008 | 0.785 | Kramer 1973 | n/a |  | 3 | Conant and Collins 1991 |
| 77 | a | *Rana catesbeiana* | Anura | 13000 | Howard 1978 | 12.94 | Semlitsch and Bodie 2003 | n/a |  | 12 | Conant and Collins 1991; Gibbs et al. 2007 |
| 77 | a | *Rana pipiens* | Anura | 3000 | Gibbs et al. 2007 | 135.95 | Blomquist and Hunter 2009 | n/a |  | 7 | Conant and Collins 1991 |
| 78 | a | *Bombina variegata* | Anura | 72.5 | Kuzmin 2013a | 1.766 | Beshkov and Jameson 1980, Hartel 2008 | n/a |  | 4.1 | Spellerberg 2002 |
| 78 | a | *Bufo bufo* | Anura | 3100 | Cooper et al. 2008 | 54.73 | Sinsch 1988 | n/a |  | 11.5 | Cooper et al. 2008 |
| 78 | a | *Hyla arborea* | Anura | 1100 | Kuzmin 2013b | 7.864 | Pellet et al. 2006 | n/a |  | 4.3 | Cooper et al. 2008 |
| 78 | a | *Lissotriton vulgaris* | Caudata | 180 | Kuzmin 2013c | 2.8 | Kovar et al. 2009 | n/a |  | 9 | Spellerberg 2002 |
| 78 | a | *Rana dalmatina* | Anura | 910 | Ponsero and Joly 1998 | 0.93 | Ponsero and Joly 1998 | n/a |  | 7.5 | Cooper et al. 2008 |
| 78 | a | *Rana esculenta* | Anura | 11000 | Cooper et al. 2008 | 1.766 | Holenweg Peter 2001 | n/a |  | 9 | Cooper et al. 2008 |
| 78 | a | *Rana temporaria* | Anura | 2585 | Cooper et al 2008 | 9.397 | Kovar et al. 2009 | n/a |  | 10 | Cooper et al. 2008 |
| 78 | a | *Salamandra salamandra* | Caudata | 40 | Rebelo and Leclair 2003; Warburg et al. 1979 | 0.13 | Schulte et al. 2007 | n/a |  | 20 | Griffiths 1996, Spellerberg 2002 |
| 78 | a | *Triturus cristatus* | Caudata | 200 | Griffiths 1996 | 20.82 | Jehle and Arntzen 2000, Sinsch 2007 | 7.4 | Jehle and Arntzen 2000 | 13 | Spellerberg 2002 |
| 79 | a | *Bufo bufo* | Anura | 3100 | Cooper et al. 2008 | 54.73 | Sinsch 1988 | n/a |  | 11.5 | Cooper et al. 2008 |
| 79 | a | *Bufo calamita* | Anura | 3500 | Cooper et al. 2008 | 14.655 | Miaud and Sanuy 2005 | n/a |  | 8 | Cooper et al. 2008 |
| 79 | a | *Ichthyosaura alpestris* | Caudata | 150 | Griffiths 1996 | 3.05 | Kovar et al. 2009 | n/a |  | 10 | Spellerberg 2002 |
| 80 | a | *Ambystoma maculatum/A. jeffersonianum* | Caudata | 230.4 | Petranka 2010 | 1.899 | Faccio 2003, Semlitsch and Bodie 2003 | n/a |  | 17.2 | Conant and Collins 1991, Petranka 2010 |
| 80 | a | *Bufo americanus/B. woodhouseii fowleri* | Anura | 5850 | Gibbs et al. 2007, Green 2013 | 17.37 | Boenke 2011, Forester et al. 2006 | n/a |  | 6.6 | Conant and Collins 1991; Gibbs et al. 2007 |
| 80 | a | *Hyla versicolor* | Anura | 1305 | Jacqueline Doyle, personal communication | 9.075 | Johnson et al 2007 | n/a |  | 4 | Conant and Collins 1991; Gibbs et al. 2007 |
| 80 | a | *Notophthalmus viridescens* | Caudata | 304 | Petranka 2010 | 0.045 | Healy 1975 | n/a |  | 8.9 | Petranka 2010 |
| 80 | a | *Pseudacris crucifer* | Anura | 900 | Gibbs et al. 2007 | 5.89 | Delzell 1958 | n/a |  | 2.5 | Conant and Collins 1991; Gibbs et al. 2007 |
| 80 | a | *Rana catesbeiana* | Anura | 16000 | Gibbs et al. 2007 | 12.94 | Semlitsch and Bodie 2003 | n/a |  | 12 | Conant and Collins 1991; Gibbs et al. 2007 |
| 80 | a | *Rana clamitans* | Anura | 3250 | Gibbs et al. 2007 | 3.83 | Lamoureux et al 2002 | n/a |  | 7.3 | Gibbs et al. 2007 |
| 80 | a | *Rana palustris* | Anura | 2500 | Gibbs et al. 2007 | n/a |  | n/a |  | 6 | Gibbs et al. 2007 |
| 80 | a | *Rana sylvatica* | Anura | 690 | Karraker and Gibbs 2009 | 2.909 | Baldwin et al 2006 | n/a |  | 5.3 | Conant and Collins 1991; Gibbs et al. 2007 |
| 81 | a | *Rana pipiens* | Anura | 3000 | Gibbs et al. 2007 | 135.95 | Blomquist and Hunter 2009 | n/a |  | 7 | Conant and Collins 1991; Gibbs et al. 2007 |
| 82 | a | *Acris crepitans* | Anura | 300 | Gibbs et al. 2007 | 0.132 | Gray 1983, Lemckert 2004 | n/a |  | 2.5 | Conant and Collins 1991; Gibbs et al. 2007 |
| 82 | a | *Ambystoma texanum* | Caudata | 625 | Trauth 2012 | 0.21 | Semlitsch and Bodie 2003 | n/a |  | 15 | Petranka 2010 |
| 82 | a | *Bufo americanus* | Anura | 6000 | Cooper et al. 2008 | 34.1 | Forester et al. 2006 | n/a |  | 7 | Conant and Collins 1991 |
| 82 | a | *Hyla versicolor/H. chrysoscelis* | Anura | 1683 | Ritke et al. 1990, Jacqueline Doyle, personal communication | 9.075 | Johnson et al 2007 | n/a |  | 4 | Conant and Collins 1991 |
| 82 | a | *Pseudacris crucifer* | Anura | 900 | Gibbs et al. 2007 | 5.89 | Delzell 1958 | n/a |  | 2.5 | Conant and Collins 1991; Gibbs et al. 2007 |
| 82 | a | *Pseudacris maculata* | Anura | 325 | Whiting 2010 | 7.292 | Spencer 1964 | n/a |  | 2.8 | Conant and Collins 1991 |
| 82 | a | *Rana blairi/R. sphenocephalus* | Anura | 4625 | Crawford et al. 2013, Gibbs et al. 2007 | 706.5 | Meade 2008 | n/a |  | 6.8 | Conant and Collins 1991 |
| 82 | a | *Rana catesbeiana* | Anura | 13000 | Howard 1978 | 12.94 | Semlitsch and Bodie 2003 | n/a |  | 12 | Conant and Collins 1991; Gibbs et al. 2007 |
| 82 | a | *Rana clamitans* | Anura | 3250 | Gibbs et al. 2007 | 3.83 | Lamoureux et al 2002 | n/a |  | 6.5 | Conant and Collins 1991 |
| 83 | a | *Dendropsophus elianeae* | Anura | n/a |  | n/a |  | n/a |  | n/a |  |
| 83 | a | *Dendropsophus minutus* | Anura | 400 | Lima et al. 2006 | n/a |  | n/a |  | 2.3 | Lima et al. 2006 |
| 83 | a | *Dendropsophus nanus* | Anura | 242 | Prado and Haddad 2005 | n/a |  | 0.6 | Prado and Haddad 2005 | 2 | Brasileiro et al. 2005, Prado and Haddad 2005 |
| 83 | a | *Dermatonotus muelleri* | Anura | 6000 | Fausto Nomura, personal communication | n/a |  | n/a |  | 4.5 | Provete 2013a |
| 83 | a | *Elachistocleis bicolor* | Anura | 478 | Prado and Haddad 2005 | n/a |  | 1.4 | Prado and Haddad 2005 | 2.6 | Prado and Haddad 2005 |
| 83 | a | *Eupemphix nattereri* | Anura | 2516 | Giaretta and Facure 2006 | n/a |  | n/a |  | 4.2 | Giaretta and Facure 2006 |
| 83 | a | *Hypsiboas albopunctatus* | Anura | 899 | Guimarães et al. 2011 | n/a |  | n/a |  | 5.3 | Brasileiro et al. 2005, Guimarães et al. 2011 |
| 83 | a | *Hypsiboas raniceps* | Anura | 1991 | Prado and Haddad 2005 | n/a |  | 12.3 | Prado and Haddad 2005 | 6 | Prado and Haddad 2005 |
| 83 | a | *Leptodactylus chaquensis* | Anura | 4936 | Prado and Haddad 2005 | n/a |  | 35.3 | Prado et al. 2000 | 7.1 | Prado and Haddad 2005 |
| 83 | a | *Leptodactylus fuscus* | Anura | 214 | Prado and Haddad 2005 | n/a |  | 8.6 | Prado and Haddad 2005 | 4.4 | Prado and Haddad 2005 |
| 83 | a | *Leptodactylus labyrinthicus* | Anura | 2101 | Silva et al. 2005 | n/a |  | n/a |  | 13.2 | Silva et al. 2005 |
| 83 | a | *Leptodactylus latrans* | Anura | n/a |  | n/a |  | n/a |  | 10 | Dietl et al. 2013 |
| 83 | a | *Leptodactylus mystacinus* | Anura | 401 | Filho and Giaretta 2008 | n/a |  | n/a |  | 5.8 | Reisman 2013 |
| 83 | a | *Leptodactylus podicipinus* | Anura | 2102 | Prado and Haddad 2005 | n/a |  | 5.2 | Prado et al. 2000 | 3.7 | Prado et al. 2000 |
| 83 | a | *Physalaemus centralis* | Anura | 1872 | Provete 2013b | n/a |  | n/a |  | 3.5 | Provete 2013b |
| 83 | a | *Physalaemus cuvieri* | Anura | 473.5 | Barreto and Andrade 1995 | n/a |  | n/a |  | 2.9 | Mijares et al. 2010 |
| 83 | a | *Pseudis platensis* | Anura | 1834 | Prado and Haddad 2005, Cynthia Prado, personal communication | n/a |  | 27.2 | Prado and Haddad 2005, Cynthia Prado, personal communication | 3.7 | Fabrezi et al. 2009 |
| 83 | a | *Pseudopaludicola falcipes* | Anura | 32 | Prado and Haddad 2005 | n/a |  | 0.3 | Prado and Haddad 2005 | 1.4 | Prado and Haddad 2005 |
| 83 | a | *Rhinella schneideri* | Anura | n/a |  | n/a |  | n/a |  | n/a |  |
| 83 | a | *Scinax fuscomarginatus* | Anura | 337 | Prado and Haddad 2005 | n/a |  | 0.5 | Prado and Haddad 2005 | 2.2 | Prado and Haddad 2005 |
| 83 | a | *Scinax fuscovarius* | Anura | 2892 | Rodrigues et al. 2005 | 0.442 | Domingos Rodrigues, personal communication | 8.9 | Rodrigues et al. 2005 | 4.9 | Rodrigues et al. 2005 |
| 83 | a | *Scinax similis* | Anura | n/a |  | n/a |  | n/a |  | n/a |  |
| 84 | a | *Acris crepitans* | Anura | 300 | Gibbs et al. 2007 | 0.132 | Gray 1983, Lemckert 2004 | n/a |  | 2.5 | Conant and Collins 1991; Gibbs et al. 2007 |
| 84 | a | *Bufo americanus* | Anura | 8000 | Gibbs et al. 2007 | 34.1 | Forester et al. 2006 | n/a |  | 7 | Conant and Collins 1991; Gibbs et al. 2007 |
| 84 | a | *Bufo fowleri* | Anura | 3700 | Green 2012 | 0.64 | Boenke 2011 | n/a |  | 6.3 | Conant and Collins 1991; Gibbs et al. 2007 |
| 84 | a | *Hyla versicolor/chrysoscelis* | Anura | 1750 | Gibbs et al. 2007, Resetarits and Wilbur 1989 | 9.075 | Johnson et al 2007 | n/a |  | 4 | Conant and Collins 1991 |
| 84 | a | *Notophthalmus viridescens* | c | 304 | Petranka 2010 | 0.045 | Healy 1975 | n/a |  | 8.9 | Petranka 2010 |
| 84 | a | *Pseudacris crucifer* | Anura | 900 | Gibbs et al. 2007 | 5.89 | Delzell 1958 | n/a |  | 2.5 | Conant and Collins 1991; Gibbs et al. 2007 |
| 84 | a | *Rana catesbeiana* | Anura | 16000 | Gibbs et al. 2007 | 12.94 | Semlitsch and Bodie 2003 | n/a |  | 12 | Conant and Collins 1991; Gibbs et al. 2007 |
| 84 | a | *Rana clamitans* | Anura | 3250 | Gibbs et al. 2007 | 3.83 | Lamoureux et al 2002 | n/a |  | 6.5 | Conant and Collins 1991 |
| 84 | a | *Rana palustris* | Anura | 2500 | Gibbs et al. 2007 | n/a |  | n/a |  | 6 | Conant and Collins 1991 |
| 84 | a | *Rana sylvatica* | Anura | 690 | Karraker and Gibbs 2009 | 2.909 | Baldwin et al 2006 | n/a |  | 5.3 | Conant and Collins 1991; Gibbs et al. 2007 |
| 84 | a | *Rana utricularia* | Anura | 4000 | Gibbs et al. 2007 | 706.5 | Meade 2008 | n/a |  | 6.3 | Gibbs et al. 2007 |
| 85 | a | *Ambystoma maculatum* | Caudata | 257 | Petranka 2010 | 1.093 | Semlitsch and Bodie 2003 | n/a |  | 20 | Petranka 2010 |
| 85 | a | *Rana sylvatica* | Anura | 690 | Karraker and Gibbs 2009 | 2.909 | Baldwin et al 2006 | n/a |  | 5.3 | Conant and Collins 1991; Gibbs et al. 2007 |
| 86 | a | *Crinia parinsignifera* | Anura | n/a |  | n/a |  | n/a |  | 2.1 | Tyler and Knight 2009 |
| 86 | a | *Crinia signifera* | Anura | 125 | Hero et al. 2005 | 0.502 | Westgate et al. 2012 | n/a |  | 2.5 | Hero et al. 2005 |
| 86 | a | *Limnodynastes dumerilii* | Anura | 3900 | Hero 2013 | 1.96 | Carthew et al. 2009 | n/a |  | 6.9 | Tyler and Knight 2009 |
| 86 | a | *Limnodynastes peronii* | Anura | 857 | Hero et al. 2005 | 0.159 | Martin Westgate, personal communication | n/a |  | 5.9 | Tyler and Knight 2009 |
| 86 | a | *Limnodynastes tasmaniensis* | Anura | 1000 | Wilson et al. 2012 | n/a |  | n/a |  | 3.8 | Tyler and Knight 2009 |
| 86 | a | *Litoria ewingii/L. paraewingi* | Anura | 600 | Hero and Bishop 2013 | n/a |  | n/a |  | 3.5 | Tyler and Knight 2009 |
| 86 | a | *Litoria peronii* | Anura | 1750 | Sherman et al. 2008 | n/a |  | n/a |  | 5.2 | Tyler and Knight 2009 |
| 87 | a | *Ambystoma maculatum* | Caudata | 268.8 | Karraker 2007 | 1.093 | Semlitsch and Bodie 2003 | n/a |  | 20 | Petranka 2010 |
| 87 | a | *Rana sylvatica* | Anura | 690 | Karraker and Gibbs 2009 | 2.909 | Baldwin et al 2006 | n/a |  | 5.3 | Conant and Collins 1991; Gibbs et al. 2007 |
| 88 | a | *Rana arvalis* | Anura | 1500 | Spellerberg 2002, Kuzmin 2013d | 14.92 | Kovar et al. 2009 | n/a |  | 7 | Cooper et al. 2008 |
| 89 | a | *Hyla arborea* | Anura | 1100 | Kuzmin 2013b | 7.864 | Pellet et al. 2006 | n/a |  | 4.3 | Cooper et al. 2008 |
| 90 | a | *Pseudacris crucifer* | Anura | 900 | Fisher et al. 2007 | 5.89 | Delzell 1958 | n/a |  | 2.5 | Conant and Collins 1991; Gibbs et al. 2007 |
| 90 | a | *Pseudacris triseriata* | Anura | 1000 | Gibbs et al. 2007, Cooper et al. 2008 | 0.785 | Kramer 1973 | n/a |  | 3 | Conant and Collins 1991; Gibbs et al. 2007 |
| 91 | a | *Bombina variegata* | Anura | 63 | Barandun et al. 1997 | 1.65 | Barandun and Reyer 1998 | n/a |  | 4.1 | Spellerberg 2002 |
| 91 | a | *Bufo bufo* | Anura | 3100 | Cooper et al. 2008 | 54.73 | Sinsch 1988 | n/a |  | 11.5 | Cooper et al. 2008 |
| 91 | a | *Bufo calamita* | Anura | 3500 | Cooper et al. 2008 | 14.655 | Miaud and Sanuy 2005 | n/a |  | 8 | Cooper et al. 2008 |
| 91 | a | *Hyla arborea* | Anura | 1100 | Kuzmin 2013b | 7.864 | Pellet et al. 2006 | n/a |  | 4.3 | Cooper et al. 2008 |
| 91 | a | *Ichthyosaura alpestris* | Caudata | 150 | Griffiths 1996 | 3.05 | Kovar et al. 2009 | n/a |  | 10 | Spellerberg 2002 |
| 91 | a | *Lissotriton helveticus* | Caudata | 365 | van der Meijden and Cavagnaro 2013 | 1.766 | Diego-Rasilla and Luengo 2007 | n/a |  | 6.5 | Spellerberg 2002 |
| 91 | a | *Lissotriton vulgaris* | Caudata | 180 | Kuzmin 2013c | 2.8 | Kovar et al. 2009 | n/a |  | 9 | Spellerberg 2002 |
| 91 | a | *Rana dalmatina* | Anura | 910 | Ponsero and Joly 1998 | 0.93 | Ponsero and Joly 1998 | n/a |  | 7.5 | Cooper et al. 2008 |
| 91 | a | *Rana ridibunda* | Anura | 1200 | Cooper et al 2008 | 2.834 | Holenweg Peter 2001 | n/a |  | 10 | Cooper et al. 2008 |
| 91 | a | *Rana temporaria* | Anura | 2585 | Cooper et al 2008 | 9.397 | Kovar et al. 2009 | n/a |  | 10 | Cooper et al. 2008 |

*Citations for studies included in the meta-analysis are provided in Reference List S1

^+^Taxa: m = mammal, b = bird, r = reptile, a = amphibian

^a^Repro = reproductive rate = mean litter or clutch size multiplied by the mean number of litters or clutches per year

^b^Home Range = mean annual home range or seasonal migration distance across both sexes

^c^Mass = body mass = average body mass of the two sexes

^d^Length = body length = average total body length of the two sexes

^§^Source = Citations for studies used for species trait information are provided in Reference List S2
