# Supplementary material for: Low Reproductive Rate Predicts Species Sensitivity to Habitat Loss: A Meta-Analysis of Wetland Vertebrates
Source: PLoS One. 2014 Mar 20;9(3):e90926. doi: 10.1371/journal.pone.0090926 (PMC3961235; doi:10.1371/journal.pone.0090926)
Supplement: Table S3 — Results from univariate mixed-effects meta-analytic models testing the effect of mobility (home range size and body length) and reproductive rate on species responses to wetland habitat loss at the population-level across and within taxonomic groups. (DOCX) [file pone.0090926.s005.docx]

Table S3: Results from univariate mixed-effects meta-analytic models testing the effect of mobility (home range size and body length) and reproductive rate on species responses to wetland habitat loss at the population-level across and within taxonomic groups.

| Group | Moderator variable | ES_Z_*_r_*^a^ | ES*_r_*^b^ | CI_lower_ | CI_upper_ | Q_E_^c^ | Q_M_^d^ | p (Q_M_) | n |
| --- | --- | --- | --- | --- | --- | --- | --- | --- | --- |
| All Taxa | Reproductive Rate | -0.046 | -0.046 | -0.067 | -0.025 | 672.762 | 18.829 | <0.0001 | 334 |
|  | Home Range Size | -0.009 | -0.009 | -0.032 | 0.015 | 708.151 | 0.536 | 0.464 | 334 |
|  | Body Length | 0.119 | 0.118 | 0.061 | 0.177 | 665.864 | 16.022 | <0.0001 | 334 |
|  | Reproductive Rate + Body Length^e^ |  |  |  |  | 661.251 | 20.761 | <0.0001 | 334 |
|  | *Reproductive Rate* | -0.032 | -0.032 | -0.061 | -0.003 |  |  |  | 334 |
|  | *Body Length* | 0.056 | 0.056 | -0.025 | 0.137 |  |  |  | 334 |
|  | Taxon |  |  |  |  | 621.388 | 25.169 | <0.0001 | 334 |
|  | *Mammal* | 0.303 | 0.294 | 0.125 | 0.48 |  |  |  | 6 |
|  | *Bird* | 0.198 | 0.196 | 0.153 | 0.243 |  |  |  | 115 |
|  | *Reptile* | 0.087 | 0.087 | -0.002 | 0.175 |  |  |  | 189 |
|  | *Amphibian* | 0.074 | 0.074 | 0.044 | 0.104 |  |  |  | 24 |
|  | Study Type |  |  |  |  | 695.378 | 0.649 | 0.421 | 334 |
|  | *Amount-based* | 0.108 | 0.107 | 0.08 | 0.134 |  |  |  | 256 |
|  | *Configuration-based* | 0.132 | 0.132 | 0.08 | 0.185 |  |  |  | 78 |
|  | Sampling Effort |  |  |  |  | 689.673 | 4.341 | 0.114 | 334 |
|  | *Dependent* | 0.138 | 0.137 | 0.104 | 0.172 |  |  |  | 191 |
|  | *Independent* | 0.091 | 0.091 | 0.051 | 0.131 |  |  |  | 123 |
|  | *Unknown* | 0.07 | 0.07 | -0.008 | 0.148 |  |  |  | 20 |
|  | Sampled Wetland Area |  |  |  |  | 711.241 | 1.26 | 0.262 | 334 |
|  | *Included* | 0.125 | 0.124 | 0.093 | 0.157 |  |  |  | 185 |
|  | *Not Included* | 0.096 | 0.096 | 0.057 | 0.135 |  |  |  | 149 |
|  | Study Region |  |  |  |  | 776.636 | 4.632 | 0.201 | 426 |
|  | *Natural* | 0.113 | 0.113 | 0.046 | 0.180 |  |  |  | 58 |
|  | *Agricultural* | 0.136 | 0.136 | 0.099 | 0.174 |  |  |  | 188 |
|  | *Rural* | 0.122 | 0.121 | 0.084 | 0.160 |  |  |  | 132 |
|  | *Urban* | 0.050 | 0.050 | -0.021 | 0.021 |  |  |  | 88 |
|  | Number of Scales | -0.032 | -0.032 | -0.084 | 0.020 | 778.399 | 1.479 | 0.224 | 426 |
| Birds | Reproductive Rate | -0.305 | -0.295 | -0.546 | -0.063 | 158.508 | 6.09 | 0.014 | 115 |
|  | Home Range Size | -0.003 | -0.003 | -0.032 | 0.025 | 168.645 | 0.052 | 0.819 | 115 |
|  | Body Length | 0.152 | 0.151 | 0 | 0.303 | 160.317 | 3.863 | 0.049 | 115 |
|  | Body Mass | -0.008 | -0.008 | -0.037 | 0.021 | 165.693 | 0.315 | 0.575 | 115 |
|  | Reproductive Rate + Body Length^c^ |  |  |  |  | 155.038 | 7.749 | 0.021 | 115 |
|  | *Reproductive Rate* | -0.253 | -0.248 | -0.506 | -0.001 |  |  |  |  |
|  | *Body Length* | 0.103 | 0.102 | -0.056 | 0.261 |  |  |  |  |
|  | Order |  |  |  |  | 151.493 | 9.887 | 0.195 | 115 |
|  | *Anseriformes* | 0.072 | 0.072 | -0.258 | 0.403 |  |  |  | 2 |
|  | *Charadriiformes* | 0.143 | 0.142 | 0.043 | 0.243 |  |  |  | 20 |
|  | *Ciconiiformes* | 0.25 | 0.244 | 0.163 | 0.336 |  |  |  | 28 |
|  | *Falconiformes* | 0.07 | 0.069 | -0.224 | 0.363 |  |  |  | 5 |
|  | *Gruiformes* | 0.115 | 0.114 | 0.023 | 0.207 |  |  |  | 23 |
|  | *Passeriformes* | 0.238 | 0.234 | 0.143 | 0.333 |  |  |  | 25 |
|  | *Pelecaniformes* | 0.343 | 0.33 | 0.092 | 0.593 |  |  |  | 3 |
|  | *Podicipediformes* | 0.265 | 0.259 | 0.132 | 0.398 |  |  |  | 9 |
|  | Study Type |  |  |  |  | 168.005 | 0.851 | 0.356 | 115 |
|  | *Amount-based* | 0.202 | 0.2 | 0.158 | 0.247 |  |  |  | 100 |
|  | *Configuration-based* | 0.111 | 0.11 | -0.079 | 0.3 |  |  |  | 15 |
|  | Sampling Effort |  |  |  |  | 168.453 | 0.116 | 0.944 | 115 |
|  | *Dependent* | 0.193 | 0.19 | 0.132 | 0.253 |  |  |  | 68 |
|  | *Independent* | 0.205 | 0.202 | 0.14 | 0.269 |  |  |  | 46 |
|  | *Unknown* | 0.151 | 0.15 | -0.28 | 0.583 |  |  |  | 1 |
|  | Sampled Wetland Area |  |  |  |  | 168.649 | 0.486 | 0.486 | 115 |
|  | *Included* | 0.191 | 0.189 | 0.145 | 0.238 |  |  |  | 85 |
|  | *Not Included* | 0.237 | 0.232 | 0.119 | 0.354 |  |  |  | 30 |
| Reptiles | Reproductive Rate | 0.044 | 0.044 | -0.338 | 0.404 | 35.294 | 0.085 | 0.771 | 24 |
|  | Home Range Size | -0.033 | -0.033 | -0.139 | 0.073 | 34.748 | 0.37 | 0.543 | 24 |
|  | Body Length | 0.039 | 0.039 | -0.212 | 0.289 | 35.278 | 0.093 | 0.761 | 24 |
|  | Order |  | 0 |  |  |  |  |  | 24 |
|  | *Squamata* | 0.15 | 0.149 | -0.025 | 0.326 | 33.956 | 0.664 | 0.415 | 5 |
|  | *Testudine* | 0.067 | 0.067 | -0.029 | 0.163 |  |  |  | 19 |
|  | Study Type |  | 0 |  |  | 25.549 | 6.104 | 0.014 | 24 |
|  | *Amount-based* | 0.046 | 0.046 | -0.034 | 0.126 |  |  |  | 18 |
|  | *Configuration-based* | 0.309 | 0.299 | 0.116 | 0.501 |  |  |  | 6 |
|  | Sampling Effort |  | 0 |  |  | 27.064 | 4.626 | 0.099 | 24 |
|  | *Area-Dependent* | 0.26 | 0.254 | 0.082 | 0.438 |  |  |  | 9 |
|  | *Area-Independent* | 0.042 | 0.042 | -0.047 | 0.13 |  |  |  | 14 |
|  | *Unknown* | 0.09 | 0.09 | -0.279 | 0.46 |  |  |  | 1 |
|  | Sampled Wetland Area |  | 0 |  |  | 31.256 | 1.993 | 0.158 | 24 |
|  | *Included* | 0.044 | 0.044 | 0.028 | 0.306 |  |  |  | 13 |
|  | *Not Included* | 0.167 | 0.165 | -0.056 | 0.143 |  |  |  | 11 |
| Amphibians | Reproductive Rate | -0.011 | -0.011 | -0.062 | 0.04 | 411.101 | 0.171 | 0.679 | 189 |
|  | Home Range Size | -0.019 | -0.019 | -0.057 | 0.018 | 411.004 | 1.018 | 0.313 | 189 |
|  | Body Length | -0.025 | -0.025 | -0.15 | 0.1 | 411.859 | 0.153 | 0.696 | 189 |
|  | Order |  |  |  |  | 411.422 | 0.183 | 0.669 | 189 |
|  | *Anura* | 0.07 | 0.07 | 0.034 | 0.105 |  |  |  | 145 |
|  | *Caudata* | 0.085 | 0.084 | 0.026 | 0.143 |  |  |  | 44 |
|  | Family |  |  |  |  | 366.036 | 18.081 | 0.054 | 189 |
|  | *Ambystomatidae* | 0.024 | 0.023 | -0.074 | 0.121 |  |  |  | 17 |
|  | *Bombinatoridae* | -0.01 | -0.01 | -0.212 | 0.191 |  |  |  | 3 |
|  | *Bufonidae* | -0.054 | -0.054 | -0.146 | 0.037 |  |  |  | 23 |
|  | *Hylidae* | 0.124 | 0.123 | 0.059 | 0.189 |  |  |  | 44 |
|  | *Leiuperidae* | 0.569 | 0.515 | -0.449 | 1.588 |  |  |  | 1 |
|  | *Myobatrachidae* | 0.031 | 0.031 | -0.111 | 0.174 |  |  |  | 10 |
|  | *Pelobatidae* | -0.036 | -0.036 | -0.427 | 0.354 |  |  |  | 1 |
|  | *Plethodontidae* | -0.277 | -0.27 | -1.296 | 0.742 |  |  |  | 1 |
|  | *Ranidae* | 0.083 | 0.083 | 0.031 | 0.136 |  |  |  | 62 |
|  | *Salamandridae* | 0.119 | 0.118 | 0.048 | 0.19 |  |  |  | 26 |
|  | *Scaphiopodidae* | 0.681 | 0.592 | -0.029 | 1.391 |  |  |  | 1 |
|  | Study Type |  |  |  |  | 403.452 | 2.844 | 0.092 | 189 |
|  | *Amount-based* | 0.057 | 0.057 | 0.021 | 0.093 |  |  |  | 133 |
|  | *Configuration-based* | 0.114 | 0.114 | 0.058 | 0.17 |  |  |  | 56 |
|  | Sampling Effort |  |  |  |  | 402.658 | 4.624 | 0.099 | 189 |
|  | *Dependent* | 0.102 | 0.102 | 0.61 | 0.143 |  |  |  | 109 |
|  | *Independent* | 0.027 | 0.027 | -0.027 | 0.082 |  |  |  | 62 |
|  | *Unknown* | 0.067 | 0.067 | -0.012 | 0.146 |  |  |  | 18 |
|  | Sampled Wetland Area |  |  |  |  | 412.169 | 0.313 | 0.576 | 189 |
|  | *Included* | 0.083 | 0.083 | 0.039 | 0.127 |  |  |  | 83 |
|  | *Not Included* | 0.065 | 0.065 | 0.023 | 0.108 |  |  |  | 106 |
| Ranidae | Reproductive Rate | 0.01 | 0.01 | -0.083 | 0.103 | 64.59 | 0.042 | 0.838 | 62 |
|  | Home Range Size | -0.015 | -0.015 | -0.074 | 0.044 | 63.421 | 0.253 | 0.615 | 62 |
|  | Body Length | -0.035 | -0.035 | -0.429 | 0.359 | 64.627 | 0.03 | 0.862 | 62 |
|  | Study Type |  |  |  |  | 95.585 | 0.84 | 0.359 | 62 |
|  | *Amount-based* | 0.071 | 0.071 | 0.021 | 0.121 |  |  |  | 47 |
|  | *Configuration-based* | 0.123 | 0.122 | 0.024 | 0.222 |  |  |  | 15 |
|  | Sampling Effort |  |  |  |  | 91.994 | 1.834 | 0.400 | 62 |
|  | *Dependent* | 0.107 | 0.107 | 0.046 | 0.167 |  |  |  | 39 |
|  | *Independent* | 0.036 | 0.035 | -0.049 | 0.121 |  |  |  | 18 |
|  | *Unknown* | 0.073 | 0.073 | -0.039 | 0.186 |  |  |  | 5 |
|  | Sampled Wetland Area |  |  |  |  | 94.134 | 2.859 | 0.091 | 62 |
|  | *Included* | 0.125 | 0.124 | 0.058 | 0.191 |  |  |  | 40 |
|  | *Not Included* | 0.049 | 0.049 | -0.009 | 0.107 |  |  |  | 22 |

^a^ mean-weighted Z-transformed correlation coefficients (ES_Z_*_r_*)

^b^ back-transformed correlation coefficients (ES*r*)

^c^ residual heterogeneity

^d^ between group/model heterogeneity

^e^ multiple meta-regression
