# Supplementary material for: Low Reproductive Rate Predicts Species Sensitivity to Habitat Loss: A Meta-Analysis of Wetland Vertebrates
Source: PLoS One. 2014 Mar 20;9(3):e90926. doi: 10.1371/journal.pone.0090926 (PMC3961235; doi:10.1371/journal.pone.0090926)
Supplement: Table S4 — Descriptive statistics of species traits (untransformed values) across all wetland vertebrates and within taxonomic groups used in the meta-analysis. (DOCX) [file pone.0090926.s006.docx]

Table S4: Descriptive statistics of species traits (untransformed values) across all wetland vertebrates and within taxonomic groups used in the meta-analysis.

| Group | Variable | Mean | Median | Min | Max |
| --- | --- | --- | --- | --- | --- |
| All Taxa (n = 334) | Home Range^a^ | 287.0 | 3.8 | 0.0 | 35600.0 |
|  | Body Length^b^ | 26.1 | 12.0 | 2.5 | 1200.0 |
|  | Reproductive rate^c^ | 1505.0 | 200.0 | 2.0 | 16000.0 |
| Mammals (n = 6) | Home Range | 3.0 | 0.5 | 0.1 | 13.0 |
|  | Body Length | 38.3 | 25.0 | 12.4 | 120.0 |
|  | Reproductive rate | 13.1 | 10.0 | 3.0 | 33.8 |
| Birds (n = 114) | Home Range | 775.9 | 9.7 | 0.0 | 35600.0 |
|  | Body Length | 53.4 | 32.0 | 10.5 | 1200.0 |
|  | Reproductive rate | 5.8 | 4.5 | 2.0 | 17.0 |
| Reptiles (n = 24) | Home Range | 96.1 | 6.1 | 0.6 | 1740.5 |
|  | Body Length | 33.6 | 20.2 | 8.3 | 99.0 |
|  | Reproductive rate | 18.1 | 14.4 | 3.5 | 36.6 |
| Amphibians (n = 189) | Home Range | 22.5 | 3.1 | 0.0 | 706.5 |
|  | Body Length | 8.1 | 7.0 | 2.5 | 34.0 |
|  | Reproductive rate | 2657.7 | 1000.0 | 40.0 | 16000.0 |

^a^Home Range (ha) = mean annual home range or seasonal migration distance across both sexes

^b^Length (cm) = body length = average total body length of the two sexes

^c^Reproductive rate = mean litter or clutch size multiplied by the mean number of litters or clutches per year
