# Supplementary material for: Low Reproductive Rate Predicts Species Sensitivity to Habitat Loss: A Meta-Analysis of Wetland Vertebrates
Source: PLoS One. 2014 Mar 20;9(3):e90926. doi: 10.1371/journal.pone.0090926 (PMC3961235; doi:10.1371/journal.pone.0090926)
Supplement: Reference List S1 — Studies included in the meta-analysis. (DOCX) [file pone.0090926.s008.docx]

Reference List S1: Studies included in the meta-analysis

1. Browne, C.L., Paszkowski, C.A., Foote, A. L., Moenting, A., Boss, S.M. 2009. The relationship of amphibian abundance to habitat features across spatial scales in the Boreal Plains. Ecoscience 16:209-223.

2. Eubanks, B.W., Hellgren, E.C., Nawrot, J.R., Bluett, R.D. 2011. Habitat associations of the marsh rice rat (*Oryzomys palustris*) in freshwater wetlands of southern Illinois. Journal of Mammalogy 92:552-560.

3. Francl, K.E., Castleberry, S.B., Ford, W.M. 2004. Small mammal communities of high elevation central Appalachian wetlands. American Midland Naturalist 151:388-398.

4. Schmidt, P.M., McCleery, R.A., Lopez, R.R., Silvy, N.J., Schmidt, J.A., Perry, N.D. 2011. Influence of patch, habitat, and landscape characteristics on patterns of Lower Keys marsh rabbit occurrence following Hurricane Wilma. Landscape Ecol 26:1419-1431.

5. Schooley, R.L., Branch, L.C. 2009. Enhancing the area–isolation paradigm: habitat heterogeneity and metapopulation dynamics of a rare wetland mammal. Ecological Applications 19:1708-1722.

6. Amano, T., Kusumoto, Y., Tokuoka, Y., Yamada, S., Kim, E.-Y., Yamamoto, S. 2008. Spatial and temporal variations in the use of rice-paddy dominated landscapes by birds in Japan. Biological Conservation 141:1704-1716.

7. Bolenbaugh, J.R., Krementz, D.G., Lehnen, S.E. 2011. Secretive marsh bird species co-occurrences and habitat associations across the Midwest, USA. Journal of Fish and Wildlife Management 2:49-60.

8. Budd, M.J. 2007. Status, distribution, and habitat selection of secretive marsh birds in the Delta of Arkansas. M.Sc. Thesis, University of Arkansas, Fayetteville, Arkansas, USA.

9. Cardador, L., Carrete, M., Mañosa, S. 2011. Can intensive agricultural landscapes favour some raptor species? The marsh harrier in north-eastern Spain. Animal Conservation 14:382-390.

10. Foppen, R.P.B., Chardon, J.P., Liefveld, W. 2000. Understanding the role of sink patches in source-sink metapopulations: reed warbler in an agricultural landscape. Conservation Biology 14:1881-1892.

11. Gibbs, J.P. 1991. Spatial relationships between nesting colonies and foraging areas of great blue herons. The Auk 108:764-770.

12. Gibbs, J.P., Kinkel, L.K. 1997. Determinants of the size and location of great blue heron colonies. Colonial Waterbirds 20:1-7.

13. Gibbs, J.P., Longcore, J.R., McAuley, D.G., Ringelman, J.K. 1991. Use of wetland habitats by selected nongame water birds in Maine. U.S. Fish and Wildlife Service, Fish and Wildlife Research Report 9, Washington D.C.

14. Gilbert, G., Tyler, G.A., Dunn, C.J., Smith, K.W. 2005. Nesting habitat selection by bitterns *Botaurus stellaris* in Britain and the implications for wetland management. Biological Conservation 124:547-553.

15. Grandmaison, D.D., Niemi, G.J. 2007. Local and landscape influence on red-winged blackbird (*Agelaius phoeniceus*) nest success in Great Lakes coastal wetlands. J. Great Lakes Res. 33 (Special Issue 3):292-304.

16. Guadagnin, D.L., Maltchik, L. 2007. Habitat and landscape factors associated with neotropical waterbird occurrence and richness in wetland fragments. Biodivers. Conserv. 16:1231-1244.

17. Hay, S. 2006. Distribution and habitat of the least bittern and other marsh bird species in southern Manitoba. Masters of Natural Resource Management thesis. University of Manitoba, Winnipeg, Manitoba, Canada.

18. Kelly, J. P., Stralberg, D., Etienne, K. L., McCaustland, M. 2008. Landscape influences on the quality of heron and egret colony sites. Wetlands 28: 257-275.

19. Maclean, I., Hassall, M., Boar, R.R., Lake, I.R. 2006. Effects of disturbance and habitat loss on papyrus-dwelling passerines. Biological Conservation 131:349-358.

20. Massey, B., Bowen, R., Griffin, C., McGarigal, K. 2008. A classification-tree analysis of nesting habitat in an island population of northern harriers. The Condor 110:177-183.

21. Moreno-Mateos, D., Pedrocchi, C., Comín, F.A. 2009. Avian communities’ preferences in recently created agricultural wetlands in irrigated landscapes of semi-arid areas. Biodivers Conserv 18:811-828.

22. Niemuth, N.D., Estey, M.E., Reynolds, R.E. 2012. Factors influencing presence and detection of breeding shorebirds in the Prairie Pothole Region of North Dakota, South Dakota, and Montana, USA. Wader Study Group Bull. 119:37-45.

23. Pierluissi, S., King, S.L., Kaller, M.D. 2010. Waterbird nest density and nest survival in rice fields of southwestern Louisiana. Waterbirds 33:323-330.

24. Quesnelle, P.E., Fahrig, L., Lindsay, K.E. 2013. Effects of habitat loss, habitat configuration and matrix composition on declining wetland species. Biological Conservation 160:200-208.

25. Rehm, E.M., Baldassarre, G.A. 2007. The influence of interspersion on marsh bird abundance in New York. The Wilson Journal of Ornithology 119:648-654.

26. Sánchez-Zapata, J.A., Anadón, J.D., Carrete, M., Giménez, A., Navarro, J., Villacorta, C., Botella, F. 2005. Breeding waterbirds in relation to artificial pond attributes: implications for the design of irrigation facilities. Biodiversity and Conservation 14:1627-1639.

27. Sripanomyom, S., Round, P.D., Savini, T., Trisurat, Y., Gale, G.A. 2011. Traditional salt-pans hold major concentrations of overwintering shorebirds in Southeast Asia. Biological Conservation 144:526–537.

28. Taft, W.O., Haig, S.M., 2006. Landscape context mediates influence of local food abundance on wetland use by wintering shorebirds in an agricultural valley. Biological Conservation 128:298-307.

29. Tourenq, C., Benhamoub, S., Sadoula, N., Sandoza, A., Mesléarda, F., Martinb, J.-L., Hafner, H. 2004. Spatial relationships between tree-nesting heron colonies and rice fields in the Camargue, France. The Auk 121:192-202.

30. Tozer, D.C., E. Nol, K.F. Abraham. 2010. Effects of local and landscape-scale habitat variables on abundance and reproductive success of wetland birds. Wetl. Ecol. Manag. 18, 679-693.

31. Uyehara, K.J., Engilis Jr., A., Dugger, B.D. 2008. Wetland features that influence occupancy by the endangered Hawaiian duck. The Wilson Journal of Ornithology 120:311-319.

32. Valente, J.J., King, S.L., Wilson, R.R. 2011. Distribution and habitat associations of breeding secretive marsh birds in Louisiana’s Mississippi alluvial valley. Wetlands 31:1–10.

33. Whited, D., Galatowitsch, S., Tester, J.R., Schik, K., Lehtinen, R., Hasueth, J. 2000. The importance of local and regional factors in predicting effective conservation: Planning strategies for wetland bird communities in agricultural and urban landscapes. Landscape and Urban Planning 49:49-65.

34. Attum, O., Lee, Y.M., Roe, J.H., Kingsbury, B.A. 2008. Wetland complexes and upland-wetland linkages: landscape effects on the distribution of rare and common wetland reptiles. Journal of Zoology 275:245-252.

35. Cosentino, B. J., Schooley, R. L., Phillips, C. A. 2010. Wetland hydrology, area, and isolation influence occupancy and spatial turnover of the painted turtle, *Chrysemys picta*. Landscape Ecology 25:1589-1600.

36. Dorland, A. 2012. Determining the impacts of high traffic roads on painted turtle (*Chrysemys picta marginata*) populations in eastern Ontario. M.Sc. Thesis. Carleton University, Ottawa, Ontario, Canada.

37. Joyal, L.A., McCollough, M., Hunter Jr., M.L., 2001. Landscape ecology approaches to wetland species conservation: a case study of two turtle species in southern Maine. Conserv. Biol. 15, 1755-1762.

38. Marchand, M.N., Litvaitis, J.A. 2004. Effects of habitat features and landscape composition on the population structure of a common aquatic turtle in a region undergoing rapid development. Conservation Biology 18:758-767.

39. Marshall, J.C. 2008. Population Genetics and Landscape Modeling in Water Snakes. PhD Thesis. Purdue University. West Lafayette, Indiana, USA.

40. Myers, A.T. 2011. Landscape and microhabitat drivers of bog turtle (*Glyptemys muhlenbergii*) occurrence in southeastern New York state. M.Sc.Thesis. State University of New York, Syracuse, New York, USA.

41. Rizkalla, C.E., Swihart, R.K. 2006. Community structure and differential responses of aquatic turtles to agriculturally induced habitat fragmentation. Landscape Ecology 21:1361 -1379.

42. Roe, J.H., Brinton, A.C., Georges, A. 2009. Temporal and spatial variation in landscape connectivity for a freshwater turtle in a temporally dynamic wetland system. Ecological Applications 19:1288-1299.

43. Steen, D.A., Gibbs, J.P., 2004. Effects of roads on the structure of freshwater turtle populations. Conserv. Biol. 18:1143-1148.

44. Winchell, K.M. 2011. Population response of two freshwater turtle species (*Chelydra serpentina* and *Chrysemys picta*) to an urbanization gradient in Syracuse, NY. M.Sc. Thesis. Columbia University, New York, New York, USA.

45. Baldwin, R.F., Calhoun, A.J.K., deMaynadier, P.G. 2006. The significance of hydroperiod and stand maturity for pool-breeding amphibians in forested landscapes. Can. J. Zool. 84:1604-1615.

46. van Buskirk, J. 2005. Local and landscape influence on amphibian occurrence and abundance. Ecology 86:1936-1947.

47. Corser, J.D., Dodd, C.K. Jr. 2004. Fluctuations in a metapopulation of nesting four-toed salamanders, *Hemidactylium scutatum*, in the Great Smoky Mountains National Park, USA, 1999-2003. Natural Areas Journal 24:135-140.

48. Cosentino, B. J., Schooley, R. L., Phillips, C. A. 2011. Spatial connectivity moderates the effect of predatory fish on salamander metapopulation dynamics. Ecosphere 2(8):art95. doi: 10.1890/ES11-00111.1

49. Cunningham, J.M., Calhoun, A.J. K., Glanz, W.E. 2007. Pond-breeding amphibian species richness and habitat selection in a beaver-modified landscape. Journal of Wildlife Management 71:2517-2526.

50. D’Amore, A., Hemingway, V., Wasson, K. 2010. Do a threatened native amphibian and its invasive congener differ in response to human alteration of the landscape? Biol Invasions 12:145–154.

51. Denoël, M., Ficetola, G.F. 2008. Conservation of newt guilds in an agricultural landscape of Belgium: the importance of aquatic and terrestrial habitats. Aquatic Conserv. Mar. Freshw. Ecosyst. 18:714-728.

52. Denoël, M., Lehmann, A. 2006. Multi-scale effect of landscape processes and habitat quality on newt abundance: implications for conservation. Biological Conservation 130:495-504.

53. Eigenbrod, F., Hecnar, S.J., Fahrig, L. 2008. The relative effects of road traffic and forest cover on anuran populations. Biological Conservation 141:35-46.

54. Goldberg, C.S., Waits, L.P. 2009. Using habitat models to determine conservation priorities for pond-breeding amphibians in a privately-owned landscape of northern Idaho, USA. Biological Conservation 142:1096-1104.

55. Gray, M.J., Smith, L.M., Leyva, R.I. 2004. Influence of agricultural landscape structure on a Southern High Plains, USA, amphibian assemblage. Landscape Ecology 19:719-729.

56. Guerry, A. D., Hunter Jr., M. L. 2002. Amphibian distributions in a landscape of forests and agriculture: an examination of landscape composition and configuration. Conservation Biology 16:745–754.

57. Gustafson, D.H., Malmgren, J.C., Mikusiński, G. 2011. Terrestrial habitat predicts use of aquatic habitat for breeding purposes – a study on the great crested newt (*Triturus cristatus*). Ann. Zool. Fennici. 48:295-307.

58. Hamer, A.J., Parris, K.M. 2011. Local and landscape determinants of amphibian communities in urban ponds. Ecological Applications 21:378–390.

59. Hamer, A.J., Smith, P.J., McDonnell, M.J. 2012. The importance of habitat design and aquatic connectivity in amphibian use of urban stormwater retention ponds. Urban Ecosyst 15:451–471.

60. Hartel, T., Schweiger, O, Öllerer, K., Cogălniceanu, D., Arntzen, J.W. 2010. Amphibian distribution in a traditionally managed rural landscape of Eastern Europe: probing the effect of landscape composition. Biological Conservation 143:1118–1124.

61. Heard, G.W., Scroggie, M.P., Malone, B.S. 2012. Classical metapopulation theory as a useful paradigm for the conservation of an endangered amphibian. Biological Conservation 148:156-166.

62. Hecnar, S.J., M’Closkey, R.T. 1998. Species richness patterns of amphibians in southwestern Ontario ponds. Journal of Biogeography 25:763-772.

63. Homan, R. N., Windmiller, B.S., Reed, J.M. 2004. Critical thresholds associated with habitat loss for two vernal pool-breeding amphibians. Ecological Applications 14:1547-1553.

64. Houlahan, J.E., Findlay, C.S. 2003. The effects of adjacent land use on wetland amphibian species richness and community composition. Can. J. Fish. Aquat. Sci. 60:1078-1094.

65. Jacobs, L., Houlahan, J.E. 2011. Adjacent land-use affects amphibian community composition and species richness in managed forests in New Brunswick, Canada. Can. J. For. Res. 41:1687-1697.

66. Johnson, P.T.J., McKenzie, V.J., Peterson, A.C., Kerby, J.L., Brown, J., Blaustein, A.R., Jackson, T. 2011. Regional decline of an iconic amphibian associated with elevation, land-use change, and invasive species. Conservation Biology 25: 556-566.

67. Joly, P., Miaud, C., Lehmann, A., Grolet, O. 2001. Habitat matrix effects on pond occupancy in newts. Conservation Biology 15:239-248.

68. Knapp, R.A., Matthews, K.R., Preisler, H.K., Jellison, R. 2003. Developing probabilistic models to predict amphibian site occupancy in a patchy landscape. Ecological Applications 13:1069-1082.

69. Knuston, M.G., Richardson, W.B., Reineke, D.M., Gray, B.R., Parmelee, J.R., Weick, S.E. 2004. Agricultural ponds support amphibian populations. Ecological Applications 14:669-684.

70. Lehtinen, R.M., Galatowitsch, S.M., Tester, J.R. 1999. Consequences of habitat loss and fragmentation for wetland amphibian assemblages. Wetlands 19:1-12.

71. Marsh, D.M., Fegraus, E.H., Harrison, S. 1999. Effects of breeding pond isolation on the spatial and temporal dynamics of pond use by the tungara frog *Physalaemus pustulosus*. Journal of Animal Ecology 68:804-814.

72. Mazerolle, M.J., Desrochers, A., Rochefort, L. 2005. Landscape characteristics influence pond occupancy by frogs after accounting for detectability. Ecological Applications 15:824-834.

73. McCarthy, K., Lathrop, R.G. 2011. Stormwater basins of the New Jersey coastal plain: subsidies or sinks for frogs and toads? Urban Ecosyst 14:395-413.

74. Otto, C.R.V., Forester, D.C., Snodgrass, J.W. 2007. Influences of wetland and landscape characteristics on the distribution of carpenter frogs. Wetlands 27:261-269.

75. Pellet, J., A. Guisan, and N. Perrin. 2004. A concentric analysis of the impact of urbanization on the threatened European Tree Frog in an agricultural landscape. Conservation Biology 18:1599–1606.

76. Pellet, J., Hoehn, S., Perrin, N. 2004. Multiscale determinants of tree frog (*Hyla arborea* L.) calling ponds in western Switzerland. Biodiversity and Conservation 13: 2227–2235.

77. Pillsbury, F.C., Miller, J.R. 2008. Habitat and landscape characteristics underlying anuran community structure along an urban-rural gradient. Ecological Applications 18:1107-1118.

78. Plăiaşu, R., Băncilă, R., Samoilă, C., Hartel, T., Cogălniceanu, D. 2012. Waterbody availability and use by amphibian communities in a rural landscape. Herpetological Journal 22:13-21.

79. Ray, N., Lehmann, A., Joly, P. 2002. Modeling spatial distribution of amphibian populations: a GIS approach based on habitat matrix permeability. Biodiversity and Conservation 11:2143-2165.

80. Rubbo, M. J., and J. M. Kiesecker. 2005. Amphibian breeding distribution in an urbanized landscape. Conservation Biology 19:504-511.

81. Schotthoefer, Anna M., Jason R. Rohr, Rebecca A. Cole, Anson V. Koehler, Catherine M. Johnson, Lucinda B. Johnson, and Val R. Beasley. 2011. Effects of wetland vs. landscape variables on parasite communities of *Rana pipiens*: links to anthropogenic factors. Ecological Applications 21:1257-1271.

82. Shulse, C.D., Semlitsch, R.D., Trauth, K.M., Willia, A.D. 2010. Influences of design and landscape placement parameters on amphibian abundance in constructed wetlands. Wetlands 30:915-928.

83. da Silva, F.R., Gibbs, J.P., Denise de Cerqueira Rossa-Feres. 2011. Breeding habitat and landscape correlates of frog diversity and abundance in a tropical agricultural landscape. Wetlands 31:1079–1087.

84. Simon, J.A., Snodgrass, J.W., Casey, R.E., Sparling, D.W. 2009. Spatial correlates of amphibian use of constructed wetlands in an urban landscape. Landscape Ecol. 24:361-373.

85. Skidds, D.E., Golet, F.C., Paton, P.W., Mitchell, J.C. 2007. Habitat correlates of reproductive effort in wood frogs and spotted salamanders in an urbanizing watershed. Journal of Herpetology 41:439-450.

86. Smallbone, L.T., Luck, G.W., Wassens, S. 2011. Anuran species in urban landscapes: relationships with biophysical, built environment and socio-economic factors. Landscape and Urban Planning 101:43-51.

87. Veysey J.S., Mattfeldt, S.D., Babbitt, K.J. 2011. Comparative influence of isolation, landscape, and wetland characteristics on egg-mass abundance of two pool-breeding amphibian species. Landscape Ecology 26:661-672.

88. Vos, C.C., Chardon, J.P. 1998. Effects of habitat fragmentation and road density on the distribution pattern of the moor frog *Rana arvalis*. Journal of Applied Ecology 35:44-56.

89. Vos, C.C., Stumpel, A.H.P. 1995. Comparison of habitat-isolation parameters in relation to fragmented distribution patterns in the tree frog (*Hyla arborea*). Landscape Ecology 11:203-214.

90. Werner, E.E., Relyea, R.A., Yurewicz, K.L., David K. Skelly, D.K., Davis, C.J. 2009. Comparative landscape dynamics of two anuran species: climate-driven interaction of local and regional processes. Ecological Monographs 79:503-521.

91. Zanini, F., Pellet, J., Schmidt, B.R. 2009. The transferability of distribution models across regions: an amphibian case study. Diversity and Distributions 15:469-480.
