# Supplementary material for: Low Reproductive Rate Predicts Species Sensitivity to Habitat Loss: A Meta-Analysis of Wetland Vertebrates
Source: PLoS One. 2014 Mar 20;9(3):e90926. doi: 10.1371/journal.pone.0090926 (PMC3961235; doi:10.1371/journal.pone.0090926)
Supplement: Reference List S2 — Species trait references for species used in the meta-analysis. (DOCX) [file pone.0090926.s009.docx]

Reference List S2: Species trait references for species used in the meta-analysis.

Abroe, B., Garvin, J.C., Pedersen, M.C., Whittingham, L.A., Dunn, P.O. 2007. Brood sex ratios are related to male size but not to attractiveness in common yellowthroats (*Geothlypis trichas*). The Auk 124:176-184.

Amano, T., Katayama, N. 2009. Hierarchical movement decisions in predators: effects of foraging experience at more than one spatial and temporal scale. Ecology 90:3536-3545.

Angehr, G.R. 1999. Rapid long-distance colonization of Lake Gatun, Panama, by snail kites. The Wilson Bulletin 111:265-268.

Angulo, A. 2008. *Pseudis platensis*. IUCN Red List of Threatened Species. Version 2012.2. [online] URL: [www.icunredlist.org](http://www.icunredlist.org)

Anstis, M. 2007. Tadpoles of south-eastern Australia: a guide with keys. New Holland Publishing, Australia.

Aquino, L., Reichle, S., Silvano, D., Scott, N. 2004. *Eupemphix nattereri*. IUCN Red List of Threatened Species. Version 2012.2. [online] URL: [www.icunredlist.org](http://www.icunredlist.org)

Aquino, L., Bastos, R., Kwet, A., Reichle, S., Silvano, D., Azevedo-Ramos, C., Scott, N., Baldo, D. 2010a. *Hypsiboas albopunctatus*. IUCN Red List of Threatened Species. Version 2012.2. [online] URL: [www.icunredlist.org](http://www.icunredlist.org)

Aquino, L., Bastos, R., Reichle, S., Silvano, D., Baldo, D., Langone, J. 2010b. *Scinax fuscovarius*. IUCN Red List of Threatened Species. Version 2012.2. [online] URL: [www.icunredlist.org](http://www.icunredlist.org)

Arruda, M.P., Morielle-Versute, E., Silva, A., Schneider, M.P.C., Gonçalves, E.C. 2011. Contemporary gene flow and weak genetic structuring in Rococo toad (*Rhinella schneideri*) populations in habitats fragmented by agricultural activities. Amphibia-Reptilia 32:399-411.

Attademo, A.M., Peltzer, P.M., Lajmanovich, R.C., Junges, C., Basso, A., Cabagna-Zenklusen, M. 2012. Trombiculid mites (*Hannemania* sp.) in *Leptodactylus chaquensis* (Amphibia: Anura) inhabiting selected soybean and rice agroecosystems of Argentina. Journal of Zoo and Wildlife Medicine 43:579-584.

Austin, J.E., Custer, C.M., Afton, A.D. 1998. Lesser scaup (*Aythya affinis*). The Birds of North America Online (A. Poole, Ed.). Ithaca: Cornell Lab of Ornithology; Retrieved from the Birds of North America Online: <http://bna.birds.cornell.edu.bnaproxy.birds.cornell.edu/bna/species/338>

Austin, J.E., Miller, M.R. 1995. Northern pintail (*Anas acuta*). The Birds of North America Online (A. Poole, Ed.). Ithaca: Cornell Lab of Ornithology; Retrieved from the Birds of North America Online: <http://bna.birds.cornell.edu.bnaproxy.birds.cornell.edu/bna/species/163>

Baker, B.W., Hill, E.P. 2003. Beaver, *Castor canadensis*. In Feldhamer, G.A., Thompson, B.C., Chapman, J.A., Eds. Wild mammals of North America. Biology, conservation and management. 2^nd^ e.d. The John Hopkins University Press, Baltimore, Maryland, USA.

Baldwin, R. F., Calhoun, A. J. K., deMaynadier, P.G. 2006. Conservation planning for amphibian species with complex habitat requirements: a case study using movements and habitat selection of the wood frog *Rana sylvatica*. Journal of Herpetology 40:442-453.

Bannor, B.K. and Erik Kiviat. 2002. Common gallinule (*Gallinula galeata*). The Birds of North America Online (A. Poole, Ed.). Ithaca: Cornell Lab of Ornithology; Retrieved from the Birds of North America Online: <http://bna.birds.cornell.edu.bnaproxy.birds.cornell.edu/bna/species/685>

Barandun, J., Reyer, H-.U. 1998. Reproductive ecology of *Bombina variegata*: habitat use. Copeia, 1998:497-500.

Barandun, J., Reyer, H U., Anholt, B. 1997. Reproductive ecology of *Bombina variegata*: aspects of life history. Amphibia-Reptilia 18:347-355.

Barreto, L., Andrade, G.V. 1995. Aspects of the reproductive biology of *Physalaemus cuvieri* (Anura: Leptodactylidae) in northeastern Brazil. Amphibia-Reptilia 16:67-76.

Bennetts, R.E., Fasola, M., Hafner, H., Kayser, Y. 2000. Influence of environmental and density-dependent factors on reproduction of little egrets. The Auk 117:634-639.

Berven, K. A. 2009. Density dependence in the terrestrial stage of wood frogs: evidence from a 21-year population study. Copeia 2009:328-338.

Beshkov, V.A., Jameson, D.L. 1980. Movement and abundance of the yellow-bellied toad *Bombina variegata*. Herpetologica 36:365-370.

Beissinger, S. R., Snyder, N. F. R. 1987. Mate desertion in the snail kite. Anim. Behav. 35:477-487.

Bielefeld, R.R., Brasher, M.G., Moorman, T. E., Gray, P.N. 2010. Mottled duck (*Anas fulvigula*). The Birds of North America Online (A. Poole, Ed.). Ithaca: Cornell Lab of Ornithology; Retrieved from the Birds of North America Online: <http://bna.birds.cornell.edu.bnaproxy.birds.cornell.edu/bna/species/081>

Blomquist, S.M., Hunter, M.L. Jr. 2009. A multi-scale assessment of habitat selection and movement patterns by northern leopard frogs (*Lithobates [Rana] pipiens*) in a managed forest. Herpetological Conservation and Biology 4:142-160.

Bogner, H.E., Baldassarre, G.A. 2002. Home range, movement, and nesting of least bitterns in western New York. Wilson Bulletin 114:297-308.

Boenke, M. 2011. Terrestrial habitat and ecology of Fowler’s toads (*Anaxyrus fowleri*). M.Sc. Thesis. McGill University, Montreal, Québec, Canada.

Brasileiro, C.A., Sawaya, R.J., Kiefer, M.C., Martins, M. 2005. Amphibians of an open cerrado fragment in southeastern Brazil. Biota Neotrop. 5:93-109.

Bray, M. P., Klebenow, D.A. 1988. Feeding ecology of white-faced ibises in a Great Basin valley, USA. Colon. Waterbirds 11:24-31.

Brindock, K.M., Colwell M.A. 2011. Habitat selection by western snowy plovers during the nonbreeding season. Journal of Wildlife Management 75:786-793.

Brisbin, Jr., I. L., Mowbray, T.B. 2002. American coot (*Fulica americana*). The Birds of North America Online (A. Poole, Ed.). Ithaca: Cornell Lab of Ornithology; Retrieved from the Birds of North America Online: <http://bna.birds.cornell.edu.bnaproxy.birds.cornell.edu/bna/species/697a>

Brooks, R. J., D. Strickland, and R. J. Rutter. 2003. Reptiles and amphibians of Algonquin Provincial Park. The Friends of Algonquin Park, Whitney, Ontario Canada.

Browne, C.L., Paszkowski, C.A. 2010. Hibernation sites of western toads (*Anaxyrus boreas*): characterization and management implications. Herpetological Conservation and Biology 5:49-63.

Bryan, D.C. 1996. Family Aramidae. In del Hoyo, J., Elliott, A., Sargatal, J. Editors. Handbook of the Birds of the World, Vol. 3. Hoatzin to Auks. Lynx Edicions, Barcelona, Spain.

Bryan, D.C. 2002. Limpkin (*Aramus guarauna*). The Birds of North America Online (A. Poole, Ed.). Ithaca: Cornell Lab of Ornithology; Retrieved from the Birds of North America Online: <http://bna.birds.cornell.edu.bnaproxy.birds.cornell.edu/bna/species/627>

Bryan Jr, A.L., Meyer, K.D., Tomlinson, B.A., Lauritsen, J.A., Brooks, W.B. 2012. Foraging habitat use by breeding wood storks and the core foraging area concept. Waterbirds 35:292-300.

Bulger, J.B., Scott Jr., N.J., Seymour, R.B. 2003. Terrestrial activity and conservation of adult California red-legged frogs *Rana aurora draytonii* in coastal forests and grasslands. Biological Conservation 110:85-95.

Burger, J. 1974. Determinants of colony and nest-site selection in the silver grebe (*Podiceps occipitales*) and Rolland's grebe (*Rollandia rolland*). The Condor 76:301-306.

Butler, R. 1991. Habitat selection and time of breeding in the great blue heron (*Ardea herodias*). Ph.D. Thesis. University of British Columbia. Vancouver, British Columbia, Canada.

Caramaschi, U., Rodrigues, M.T. 2004. *Dendropsophus elianeae*. IUCN Red List of Threatened Species. Version 2012.2. [online] URL: [www.icunredlist.org](http://www.icunredlist.org)

Carboneras, C. 1992a. Family Anhimidae. In del Hoyo, J., Elliott, A., Sargatal, J. Editors. Handbook of the birds of the world, vol. 1. Ostrich to Ducks. Lynx Edicions, Barcelona, Spain.

Carboneras, C. 1992b. Family Anatidae. In del Hoyo, J., Elliott, A., Sargatal, J. Editors. Handbook of the birds of the world, vol. 1. Ostrich to Ducks. Lynx Edicions, Barcelona, Spain.

Cardador, L., Mañosa, S., Varea, A., Bertolero, A. 2009. Ranging behaviour of marsh harriers *Circus aeruginosus* in agricultural landscapes. Ibis 151:766-770.

Carrière, M.-A. 2007. Movement patterns and habitat selection of common map turtles (*Graptemys geographica*) in St. Lawrence Islands National Park, Ontario, Canada. M.Sc. Thesis. University of Ottawa, Ottawa, Ontario, Canada.

Carthew, S.M., Horner, B., Jones, K.M.W. 2009. Do utility corridors affect movements of small terrestrial fauna? Wildlife Research 36:488–495.

Catchpole, C.K. 1972. A comparative study of territory in the reed warbler *(Acrocephalus scirpaceus)* and sedge warbler *(A. schoenobaenus*). J. Zool., Lond. 166:213-231.

Catchpole, C.K. 1974. Habitat selection and breeding success in the reed warbler (*Acrocephalus scirpaceus*). Journal of Animal Ecology 43:363-380.

Catchpole, C., Leisler, B., Winkler, H. 1985. Polygyny in the great reed warbler, *Acrocephalus arundinaceus*: a possible case of deception. Behavioral Ecology and Sociobiology 16:285-291.

Chapman Mosher, B.-A. 1986. Factors influencing reproductive success and nesting strategies in black terns. Ph.D. Thesis. Simon Fraser University, Burnaby, British Columbia, Canada.

Chernetsov N., Titov, N. 2001. Movement patterns of European reed warblers *Acrocephalus scirpaceus* and sedge warblers *A.* *Schoenobaenus* before and during autumn migration. Ardea 89:509-515.

Christiansen, J.L., Moll, E.O. 1973. Latitudinal reproductive variation within a single subspecies of painted turtle, *Chrysemys picta bellii*. Herpetologica 29:152-163.

Clement, P. 2006. Family Sylviidae. In del Hoyo, J., Elliott, A., Christie, D.A. Editors. Handbook of the Birds of the World, Vol. 11. Old world flycatchers to old world warblers. Lynx Edicions, Barcelona, Spain.

Cline, B.B., Haig, S.M. 2011. Seasonal movement, residency, and migratory patterns of Wilson's snipe (*Gallinago delicata*). The Auk 128:543-555.

Collar, N.J., Newton, I., Clement, P. 2010. Family Malaconotidae. In del Hoyo, J., Elliott, A., Christie, D.A. Editors. Handbook of the birds of the world, vol. 15. Weavers to New World Warblers. Lynx Edicions, Barcelona, Spain.

Colwell, M. A., Jehl, J. R. Jr. 1994. Wilson's phalarope (*Phalaropus tricolor*). The Birds of North America Online (A. Poole, Ed.). Ithaca: Cornell Lab of Ornithology; Retrieved from the Birds of North America Online: <http://bna.birds.cornell.edu.bnaproxy.birds.cornell.edu/bna/species/083>

Congdon, J.D., Kinney, O.M., Nagle, R.D. 2011. Spatial ecology and core-area protection of Blanding's turtle (*Emydoidea blandingii*). Can. J. Zool. 89:1098-1106.

## Congdon, J.D., van Loben Sels, R.C. 1993. Relationships of reproductive traits and body size with attainment of sexual maturity and age in Blanding's turtles (*Emydoidea blandingi*). Journal of Evolutionary Biology 6:547–557.

Connant, R., Collins, J.T. 1991. A field guide to reptiles and amphibians: eastern and central North America. 3^rd^ e.d. Houghton Mifflin, Boston, Massachusetts, USA.

Conway, C.J. 1995. Virginia rail (*Rallus limicola*). The Birds of North America Online (A. Poole, Ed.). Ithaca: Cornell Lab of Ornithology; Retrieved from the Birds of North America Online: <http://bna.birds.cornell.edu.bnaproxy.birds.cornell.edu/bna/species/173>

Cooper, N., Bielby, J., Thomas, G.H., Purvis, A. 2008. Macroecology and extinction risk correlates of frogs. Global Ecology and Biogeography, (Global Ecol. Biogeogr.)17:211-221.

Corn, P.S., Livo, L.J. 1989. Leopard frog and wood frog reproduction in Colorado and Wyoming. Northwestern Naturalist 70:1-9.

COSEWIC 2002. COSEWIC assessment and status report the stinkpot *Sternotherus odoratus*. Committee on the Status of Endangered Wildlife in Canada. Ottawa. vi + 18 pp [online] URL: <http://www.sararegistry.gc.ca/>

Crawford, J.A., Brown, L.E., Painter, C.W. 2013. AmphibiaWeb: Information on amphibian biology and conservation. *Rana blairi*. Berkeley, California: AmphibiaWeb. [online] URL: <http://amphibiaweb.org/>

Cuervo, J.J. 2004. Nest-site selection and characteristics in a mixedspecies colony of avocets *Recurvirostra avosetta* and black-winged stilts *Himantopus himantopus*. Bird Study 51:20-24.

Cuervo, J.J. 2005. Hatching success in avocet *Recurvirostra avosetta* and black-winged stilt *Himantopus himantopus*. Bird Study 52:166-172.

Custer, C.M., Galli, J. 2002. Feeding habitat selection by great blue herons and great egrets nesting in east central Minnesota. Waterbirds: The International Journal of Waterbird Biology, 25:115-124.

Custer, T.W., Osborn, R.G. 1978. Feeding habitat use by colonially-breeding herons, egrets, and ibises in North Carolina. The Auk 95:733-743.

Davis, Jr., W. E. Kushlan, J. A. 1994. Green heron (*Butorides* *virescens*). The Birds of North America Online (A. Poole, Ed.). Ithaca: Cornell Lab of Ornithology; Retrieved from the Birds of North America Online: <http://bna.birds.cornell.edu.bnaproxy.birds.cornell.edu/bna/species/129>

De Fonseca, P.H. 1982. The palmate newt *Triturus helveticus helveticus* (Raz.) in Flanders (Belgium). Distribution and habitat preferences. Biological Conservation 23:297-307.

Delzell, D. E. 1958. Spatial movement and growth of *Hyla crucifer*. Ph.D. Thesis. University of Michigan, Ann Arbor, Michigan, USA.

Demers, S.A., Colwell, M.A., Takekawa, J.Y., Ackerman, J.T. 2008. Breeding stage influences space use of female American avocets in San Francisco Bay, California. Waterbirds 31:365-371.

Diego-Rasilla, F.J., Luengo, R.M. 2007. Acoustic orientation in the palmate newt, *Lissotriton helveticus*. Behav Ecol Sociobiol 61:1329-1335.

Dietl, J., Fritz, S., Kittel, R., Sole, M. 2013. AmphibiaWeb: Information on amphibian biology and conservation. *Leptodactylus latrans*. Berkeley, California: AmphibiaWeb. [online] URL: [www.amphibiaweb.org](http://www.amphibiaweb.org)

Dijkstra C., Zijlstra, M. 1997. Reproduction of the marsh harrier *Circus aeruginosus* in recent land reclamations in The Netherlands. Ardea 85:37-50.

Drilling, N., Titman, R., Mckinney, F. 2002. Mallard (*Anas platyrhynchos*). The Birds of North America Online (A. Poole, Ed.). Ithaca: Cornell Lab of Ornithology; Retrieved from the Birds of North America Online: <http://bna.birds.cornell.edu.bnaproxy.birds.cornell.edu/bna/species/658>

Dubowy, P.J. 1996. Northern shoveler (*Anas clypeata*). The Birds of North America Online (A. Poole, Ed.). Ithaca: Cornell Lab of Ornithology; Retrieved from the Birds of North America Online: <http://bna.birds.cornell.edu.bnaproxy.birds.cornell.edu/bna/species/217>

Dumas, J.V. 2000. Roseate spoonbill (*Platalea ajaja*). The Birds of North America Online (A. Poole, Ed.). Ithaca: Cornell Lab of Ornithology; Retrieved from the Birds of North America Online: <http://bna.birds.cornell.edu.bnaproxy.birds.cornell.edu/bna/species/490>

Dyrcz, A. 2006. Family Sylviidae. In del Hoyo, J., Elliott, A., Christie, D.A. Editors. Handbook of the Birds of the World, Vol. 11. Old world flycatchers to old world warblers. Lynx Edicions, Barcelona, Spain.

Edmonds, J. H. 1999. Population ecology of the stinkpot turtle (*Sternotherus odoratus*) in Georgian Bay, Ontario. M.Sc. Thesis. University of Guelph, Guelph, Ontario, Canada.

Elliott, A. 1992. Family Ciconiidae. In del Hoyo, J., Elliott, A., Sargatal, J. Editors. Handbook of the birds of the world, vol. 1. Ostrich to ducks. Lynx Editions, Barcelona, Spain.

Elphick, C.S., Tibbitts, T.L. 1998. Greater yellowlegs (*Tringa melanoleuca*). The Birds of North America Online (A. Poole, Ed.). Ithaca: Cornell Lab of Ornithology; Retrieved from the Birds of North America Online: <http://bna.birds.cornell.edu.bnaproxy.birds.cornell.edu/bna/species/355>

Emlen, S.T., Wrege, P.H., Webster, M.S. 1998. Cuckoldry as a cost of polyandry in the sex-role-reversed wattled jacana, *Jacana jacana*. Proc. R. Soc. Lond. B 265:2359-2364.

Engilis, Jr., A., Uyehara, K.J., Giffin, J.G. 2002. Hawaiian duck (*Anas wyvilliana*). The Birds of North America Online (A. Poole, Ed.). Ithaca: Cornell Lab of Ornithology; Retrieved from the Birds of North America Online: <http://bna.birds.cornell.edu.bnaproxy.birds.cornell.edu/bna/species/694>

Ernst, C.H., Ernst, E.M. 2003. Snakes of the United States and Canada. Smithsonian Institution, Washington, D.C.

Ernst, C.H., Lovich, J.E. 2009. Turtles of the United States and Canada. 2^nd^ e.d. John Hopkins University Press, Baltimore, Maryland, USA.

Eubanks, B.W., Hellgren , E.C., Nawrot , J.R., Bluett, R.D. 2011. Habitat associations of the marsh rice rat (*Oryzomys palustris*) in freshwater wetlands of southern Illinois. Journal of Mammalogy 92:552-560.

Fabrezi, M., Quinzio, S. I., Goldberg, J. 2009. Giant tadpole and delayed metamorphosis of *Pseudis platensis* Gallardo, 1961 (Anura, Hylidae). Journal of Herpetology 43:228-243.

Faccio, S.D. 2003. Postbreeding emigration and habitat use by Jefferson and spotted salamanders in Vermont. Journal of Herpetology 37:479-489.

Faulhaber, C. A., Silvy, N.J., Lopez, R.R., LaFever, D.H., Frank, P.A., Peterson, M. J. 2008. Diurnal habitat use by Lower Keys marsh rabbits. Journal of Wildlife Management 72:1161-1167.

Fellers, G. M. 2013. AmphibiaWeb: Information on amphibian biology and conservation. *Rana draytonii*. Berkeley, California: AmphibiaWeb. [online] URL: <http://amphibiaweb.org/>

Filho, J.C.O., Giaretta, A.A. 2008. Reproductive behavior of *Leptodactylus mystacinus* (Anura, Leptodactylidae) with notes on courtship call of other *Leptodactylus* species. Iheringia. Série zoologia 98:508-515.

Finn, P.G., Catterall, C.P., Driscoll, P.V. 2007. Determinants of preferred intertidal feeding habitat for eastern curlew: a study at two spatial scales. Austral Ecology 32:131-144.

Forester, D.C., Snodgrass, J.W., Marsalek, K., Lanham, Z. 2006. Post-breeding dispersal and summer home range of female American toads (*Bufo americanus*). Northeastern Naturalist 13:59-72.

Forys, E.A. 1995. Metapopulations of marsh rabbits: a population viability analysis of the Lower Keys marsh rabbit (*Sylvilagus palustris hefneri*). Ph.D. Thesis. University of Florida, Gainesville, Florida, USA.

Forys, E.A., Humphrey, S.R. 1996. Home range and movements of the Lower Keys marsh rabbit in a highly fragmented habitat. Journal of Mammalogy 77:1042-1048.

Fisher, C., Joynt, A., Brooks, R.J. 2007. Reptiles and amphibians of Canada. Lone Pine Publishing, Edmonton, Alberta, Canada.

Fry, C.H. 2009. Family Malaconotidae. In del Hoyo, J., Elliott, A., Christie, D.A. Editors. Handbook of the birds of the world, vol. 14. Bush-shrikes to Old World sparrows. Lynx Edicions, Barcelona, Spain.

Fry, C.H., Keith, S., Urban, E.K., eds. 2000. The birds of Africa, vol VI. Princeton University Press, Princeton, New Jersey, USA.

Fry, C.H., Keith, S., eds. 2004. The birds of Africa, vol VII. Princeton University Press, Princeton, New Jersey, USA.

Galois, P., Léveillé, M., Bouthillier, L., Daigle, C., Parren, S. 2002. Movement patterns, activity, and home range of the eastern spiny softshell turtle (*Apalone spinifera*) in northern Lake Champlain, Québec, Vermont. Journal of Herpetology 36:402-411.

Giaretta, A.A., Facure, K.G. 2006. Terrestrial and communal nesting in *Eupemphix nattereri* (Anura, Leiuperidae): interactions with predators and pond structure. Journal of Natural History 40: 2577-2587.

Gibbons, J.W., Dorcas, M.E. 2004. North American watersnakes: a natural history. University of Oklahoma Press, Norman, USA.

Gibbons, J.W., Lovich, J.E. 1990. Sexual dimorphism in turtles with emphasis on the slider turtle (*Trachemys scripta*). Herpetological Monographs 4:1-29.

Gibbs, J.P., Breisch, A.R., Ducey, P.K., Johnson, G., Behler, J.L., Bothner, R.C. 2007. The amphibians and reptiles of New York state: identification, natural history, and conservation. Oxford University Press. New York, New York, USA.
Gilbert, G., Tyler, G.A., Smith, K.W. 2005. Behaviour, home-range size and habitat use by male Great Bittern *Botaurus stellaris* in Britain. Ibis 147:533-543.

Gilbert, G., Tyler, G.A., Dunn, C.J., Ratcliffe, N., Smith, K.W. 2007. The influence of habitat management on the breeding success of the Great Bittern *Botaurus stellaris* in Britain. Ibis 149:53-66.

Gilbert, M., Leclair Jr, R., Fortin, R. 1994. Reproduction of the Northern Leopard Frog (*Rana pipiens*) in floodplain habitat in the Richelieu River, P. Quebec, Canada. Journal of Herpetology 28:465-470.

Gilmer, D. S., Ball, I.J., Cowardin, L.M., Riechmann, J.H., Tester, J. R. 1975. Habitat use and home range of Mallards breeding in Minnesota. J. Wildl. Manage. 39:781-789.

van Gils, J., Wiersma, P. 1996. Family Scolopacidae. In del Hoyo, J., Elliott, A., Sargatal, J. Editors. Handbook of the birds of the world, vol. 3. Hoatzin to Auks. Lynx Edicions, Barcelona, Spain.

Given, M.F. 1988. Territoriality and aggressive interactions of male carpenter frogs, *Rana virgatipes* Mac F. Copeia 1988:411-421.

González, J.A. 1999. Nesting success in two wood stork colonies in Venezuela. Journal of Field Ornithology 70:18-27.

González, J.A. 1998. Phenology and reproductive success of the maguari stork in the southern llanos of Venezuela. Colonial Waterbirds 21:135-142.

Gratto-Trevor, C.L. 2000. Marbled Godwit (*Limosa fedoa*). The Birds of North America Online (A. Poole, Ed.). Ithaca: Cornell Lab of Ornithology; Retrieved from the Birds of North America Online: <http://bna.birds.cornell.edu.bnaproxy.birds.cornell.edu/bna/species/492>

Graves, B.M., Krupa, J.J. 2013. AmphibiaWeb: Information on amphibian biology and conservation. *Bufo cognatus*. Berkeley, California: AmphibiaWeb. [online] URL: <http://amphibiaweb.org/>

Gray, M.J. 2002. Effect of anthropogenic disturbance and landscape structure on body size, demographics, and chaotic dynamics of southern high plains amphibians. Ph.D. Thesis. Texas Tech University, Lubbock, Texas, USA.

Gray R.H. 1983. Seasonal, annual and geographic variation in color morph frequencies of the cricket frog, *Acris crepitans*, in Illinois. Copeia 1983:300-311.

Green, D.M. 2013. AmphibiaWeb: Information on amphibian biology and conservation. *Bufo fowleri*. Berkeley, California: AmphibiaWeb. [online] URL: <http://amphibiaweb.org/>

Griffiths, R. A. 1996. Newts and salamanders of Europe. T. & A. D. Poyser Natural History, London, United Kingdom.

Guimarães, T.C.S., de Figueiredo G.B., Mesquita, D.O., Vasconcellos, M.M. 2011. Ecology of *Hypsiboas albopunctatus* (Anura: Hylidae) in a neotropical savanna. Journal of Herpetology 45:244-250.

Gutiérrez, R., Figuerola, J. 1997. Estimating the size of Little Grebe (*Tachybaptus rufficollis*) breeding populations. Ardeola 44: 157–161.

Guzy, M.J., Ritchison, G. 1999. Common yellowthroat (*Geothlypis trichas*). The Birds of North America Online (A. Poole, Ed.). Ithaca: Cornell Lab of Ornithology; Retrieved from the Birds of North America Online: <http://bna.birds.cornell.edu.bnaproxy.birds.cornell.edu/bna/species/448>

Hafner, H., Britton, R.H. 1983. Changes of foraging sites by nesting little egrets (*Egretta garzetta* L.) in relation to food supply. Colonial Waterbirds 6:24-30.

Hafner, H., Bennetts, R.E., Kayser, Y. 2001. Changes in clutch size, brood size and numbers of nesting Squacco Herons *Ardeola ralloides* over a 32-year period in the Camargue, southern France. Ibis 143:11-16.

Hartel, T. 2008. Movement activity in a *Bombina variegata* population from a deciduous forested landscape. North-Western Journal of Zoology 4:79-90.

Healy, W. R. 1975. Terrestrial activity and home range in efts of *Notophthalmus viridescens*. American Midland Naturalist 93:131-138.

Heard, G.W., Scroggie, M.P., Malone, B.S. 2012a. The life history and decline of the threatened Australian frog, *Litoria raniformis*. Austral Ecology 37:276-284.

Heard, G.W., Scroggie, M.P., Malone, B.S. 2012b. Classical metapopulation theory as a useful paradigm for the conservation of an endangered amphibian. Biological Conservation 148:156-166.

Heath, S.R., Dunn, E.H., Agro, D.J. 2009. Black tern (*Chlidonias niger*), The Birds of North America Online (A. Poole, Ed.). Ithaca: Cornell Lab of Ornithology; Retrieved from the Birds of North America Online: <http://bna.birds.cornell.edu.bnaproxy.birds.cornell.edu/bna/species/147>

Hecnar, S. J., M'Closkey, R. T. 1997. Patterns of nestedness and species association in a pond-dwelling amphibian fauna. Oikos 80:371-381.

Herkert, J.R., Kroodsma, D.E., Gibbs, J.P. 2001. Sedge wren (*Cistothorus platensis*). The Birds of North America Online (A. Poole, Ed.). Ithaca: Cornell Lab of Ornithology; Retrieved from the Birds of North America Online: <http://bna.birds.cornell.edu.bnaproxy.birds.cornell.edu/bna/species/582>

Hels, T. 2002. Population dynamics in a Danish metapopulation of spadefoot toads *Pelobates fuscus*. Ecography 25:303-313.

Hero, J.-M., Williams, S.E., Magnusson, W.E. 2005. Ecological traits of declining amphibians in upland areas of eastern Australia. J. Zool., Lond*.* 267:221-232.

Hero, J.-M. 2013. AmphibiaWeb: Information on amphibian biology and conservation. *Limnodynastes dumerilii*. Berkeley, California: AmphibiaWeb. [online] URL: <http://amphibiaweb.org/>

Hero, J.-M., Bishop, P. 2013. AmphibiaWeb: Information on amphibian biology and conservation. *Litoria ewingii*. Berkeley, California: [online] URL: <http://amphibiaweb.org/>

Hero, J.-M., Clarke, J., Meyer, E., Robertson, P., Lemckert, F. 2004. *Crinia parinsignifera*. IUCN Red List of Threatened Species. Version 2012.2. [online] URL: [www.icunredlist.org](http://www.icunredlist.org)

Hero, J.-M., Hollis, G., Osborne, W., Gillespie, G., Shoo L. 2013. AmphibiaWeb: Information on amphibian biology and conservation. *Litoria verreauxii*. Berkeley, California: AmphibiaWeb. [online] URL: <http://amphibiaweb.org/>

Heyer, W. R., A. Stanley Rand; Carlos Alberto Gonçalves da Cruz; Oswaldo L. Peixoto; Craig E. Nelson. 1990. Frogs of Boracéia. Arq. Zool. v.31 n.4 São Paulo.

Heyer, R., Reichle, S., Silvano, D., Azevedo-Ramos, C., Baldo, D., Gascon, C. 2004. *Leptodactylus podicipinus*. IUCN Red List of Threatened Species. Version 2012.2. [online] URL: [www.icunredlist.org](http://www.icunredlist.org)

Hicklin, P., Gratto-Trevor, C.L. 2010. Semipalmated sandpiper (*Calidris pusilla*). The Birds of North America Online (A. Poole, Ed.). Ithaca: Cornell Lab of Ornithology; Retrieved from the Birds of North America Online: <http://bna.birds.cornell.edu.bnaproxy.birds.cornell.edu/bna/species/006>

Holenweg, A.-K., Reyer, H.-U. 2000. Hibernation behavior of *Rana lessonae* and *R. esculenta* in their natural habitat. Oecologia 123:41-47.

Holenweg Peter, A.-K. 2001. Dispersal rates and distances in adult water frogs, *Rana lessonae*, *Rana ridibunda*, and their hybridogenetic associate *Rana esculenta*. Herpetologica 57:449–46.

Holmes, R.T., Pitelka, F.A. 1964. Breeding behavior and taxonomic relationships of the curlew sandpiper. The Auk 81:362-379.

Hohman, W.L., Lee, S.A. 2001. Fulvous whistling-duck (*Dendrocygna* *bicolor*), The Birds of North America Online (A. Poole, Ed.). Ithaca: Cornell Lab of Ornithology; Retrieved from the Birds of North America Online: <http://bna.birds.cornell.edu.bnaproxy.birds.cornell.edu/bna/species/562>

Howard, J.H., Wallace, R.L. 1985. Life history characteristics of populations of the long-toed salamander (*Ambystoma macrodactylum*) from different altitudes. American Midland Naturalist 113: 361-372.

Irwin, J.T., Costanzo, J.P., Lee, Jr., R.E. 1999. Terrestrial hibernation in the northern cricket frog, *Acris crepitans*. Can. J. Zool. 77:1240-1246.

Iverson, J.B, Higgins, H., Sirulnik, A., Griffiths, C. 1997. Local and geographic variation in the reproductive biology of the snapping turtle (*Chelydra serpentina*). Herpetologica 53:96-117.

Jaeger, C.P., Cobb, V.A. 2012. Comparative spatial ecologies of female painted turtles (*Chrysemys picta*) and red-eared sliders (*Trachemys scripta*) at Reelfoot Lake, Tennessee. Chelonian Conservation and Biology 11:59-67.

Jarrett, M. 2011. "*Chelodina longicollis*". Animal Diversity Web. [online] URL: <http://animaldiversity.ummz.umich.edu/accounts/Chelodina_longicollis/>

Jehle, R., Arntzen, J. W. 2000. Post-breeding migrations of newts (*Triturus cristatus* and *T. marmoratus*) with contrasting ecological requirements. J. Zool., Lond. 251:297-306.

Jenkins, R. K. B., Buckton, S. T., Ormerod, S. J. 1995. Local movements and population density of Water Rails *Rallus aquaticus* in a small inland reedbed. Bird Study 42:82-87.

Jenni, D.A. 1996. Family Jacanidae. In del Hoyo, J., Elliott, A., Sargatal, J. Editors. Handbook of the Birds of the World, Vol. 3. Hoatzin to Auks. Lynx Edicions, Barcelona, Spain.

Jetz, W., Sekercioglu, C.H., Böhning-Gaese, K. 2008. The worldwide variation in avian clutch size across species and space. PLoS Biol 6(12): e303. doi:10.1371/journal.pbio.0060303

Johnson, R. R., Dinsmore, J. J. 1985. Brood-rearing and postbreeding habitat use by Virginia Rails and Soras. Wilson Bull. 97:551-554.

Johnson, J. R. 2005. Multi-scale investigations of gray treefrog movements: patterns of migration, dispersal and gene flow. Ph.D. Thesis. University of Missouri-Columbia, Missouri, USA.

Johnson, J. R., Knouft, J. H., Semlitsch, R. D. 2007. Sex and seasonal differences in the spatial terrestrial distribution of gray treefrog (*Hyla versicolor*) populations. Biological Conservation 140:250-258.

Johnson, K. 1995. Green-winged teal (*Anas crecca*). The Birds of North America Online (A. Poole, Ed.). Ithaca: Cornell Lab of Ornithology; Retrieved from the Birds of North America Online: <http://bna.birds.cornell.edu.bnaproxy.birds.cornell.edu/bna/species/193>

Johnson, O.W., Connors, P.G. 2010. Pacific golden-plover (*Pluvialis* *fulva*). The Birds of North America Online (A. Poole, Ed.). Ithaca: Cornell Lab of Ornithology; Retrieved from the Birds of North America Online: <http://bna.birds.cornell.edu.bnaproxy.birds.cornell.edu/bna/species/202>

Jones, K.E., et al. 2009. PanTHERIA: a species-level database of life history, ecology, and geography of extant and recently extinct mammals. Ecology 90:2648.

Joyal, L.A. 1996. Ecology of Blanding's (*Emydoidea blandingii*) and spotted (*Clemmys guttata*) turtles in southern Maine: population structure, habitat use, movements, and reproductive biology. M.Sc. Thesis. University of Maine, Orono, Maine, USA.

Joyal, L.A., McCollough, M., Hunter Jr., M.L. 2000. Population structure and reproductive ecology of Blanding's turtle (*Emydoidea blandingii*) in Maine, near the northeastern edge of its range. Chelonian Conservation and Biology 3:580-588.

Karraker, N.E. 2007. A new method for estimating clutch sizes of ambystomatid salamanders and ranid frogs: introducing the ovagram. Herpetological Review 38:46-48.

Karraker, N.E., Gibbs, J.P. 2009. Amphibian production in forested landscapes in relation to wetland hydroperiod: A case study of vernal pools and beaver ponds. Biological Conservation 142:2293-2302.

Kaufmann, G. W. 1989. Breeding ecology of the Sora *Porzana carolina*, and the Virginia Rail *Rallus limicola*. Can. Field-Nat. 3:270-282.

Keck, M.B. 2004. *Nerodia rhomiber*, diamondback watersnake. In Gibbons, J.W., Dorcas, M.E. 2004. North American watersnakes: a natural history. University of Oklahoma Press, Norman, USA.

Kennett, R.M., Georges, A. 1990. Habitat utilization and its relationship to growth and reproduction of the eastern long-necked turtle, *Chelodina longicollis* (Testudinata: Chelidae), from Australia. Herpetologica 46:22-33.

Knapp, M. 2013. AmphibiaWeb: Information on amphibian biology and conservation. *Engystomops pustulosus*. Berkeley, California: AmphibiaWeb. [online] URL: <http://amphibiaweb.org/>

Knuston, M.G., Sauer, J.R., Olsen, D.A., Mossman, M.J., Hemesath, L.M., Lannoo, M.J. 1999. Effects of landscape composition and wetland fragmentation on frog and toad abundance and species richness in Iowa and Wisconsin, U.S.A. Conservation Biology 13:1437-1446.

Kovar, R., Brabec, M., Vita, R., Bocek, R. 2009. Spring migration distances of some Central European amphibian species. Amphibia-Reptilia 30:367-378.

Kramer, D. C. 1973. Movements of western chorus frogs *Pseudacris triseriata triseriata* tagged with Co60. Journal of Herpetology **7**:231-235.

Krupa, J.J. 1994. Breeding biology of the Great Plains toad in Oklahoma. Journal of Herpetology 28:217-224.

Kroodsma, D.E., Verner, J. 1997. Marsh wren (*Cistothorus palustris*). The Birds of North America Online (A. Poole, Ed.). Ithaca: Cornell Lab of Ornithology; Retrieved from the Birds of North America Online: <http://bna.birds.cornell.edu.bnaproxy.birds.cornell.edu/bna/species/308>

Kuzmin, S. L. 2013a. AmphibiaWeb: Information on amphibian biology and conservation. *Bombina variegata*. Berkeley, California: AmphibiaWeb. [online] URL: <http://amphibiaweb.org/>

Kuzmin, S. L. 2013b. AmphibiaWeb: Information on amphibian biology and conservation. *Hyla arborea*. Berkeley, California: AmphibiaWeb. [online] URL: <http://amphibiaweb.org/>

Kuzmin, S. L. 2013c. AmphibiaWeb: Information on amphibian biology and conservation. *Lissotriton vulgaris*. Berkeley, California: AmphibiaWeb. [online] URL: <http://amphibiaweb.org/>

Kuzmin, S. L. 2013d. AmphibiaWeb: Information on amphibian biology and conservation. *Rana arvalis*. Berkeley, California: AmphibiaWeb. [online] URL: <http://amphibiaweb.org/>

Kuzmin, S. L., Andreone, F. 2013. AmphibiaWeb: Information on amphibian biology and conservation. *Pelobates fuscus*. Berkeley, California: AmphibiaWeb. [online] URL: <http://amphibiaweb.org/>

Kuzmin, S. L., Cavagnaro, J. 2013. AmphibiaWeb: Information on amphibian biology and conservation. *Rana lessonae*. Berkeley, California: AmphibiaWeb. [online] URL: <http://amphibiaweb.org/>

Lamoureux, V.S., Maerz, J.C., Madison, D.M. 2002. Premigratory autumn foraging forays in the green frog, *Rana clamitans*. Journal of Herpetology 36:245-254.

Landreth, H.F., Christensen, M.T. 1971. Orientation of the plains spadefoot toad, *Spea bombifrons*, to solar cues. Herpetologica 27:454-461.

Lauck, B. 2005. Life-history studies and the impact of recent forest harvesting on two frog species, *Crinia signifera* and *Litoria ewingii*. Tasforests 16:83-94.

Lavilla, E., Aquino, L., Kwet, A., Baldo, D. 2004. *Pseudopaludicola falcipes*. IUCN Red List of Threatened Species. Version 2012.2. [online] URL: [www.icunredlist.org](http://www.icunredlist.org)

Lemckert, F.L. 2004. Variations in anuran movements and habitat use: implications for conservation. Applied Herpetology 1:165-181.

Leonard, M.L., Picma, J. 1986. Why are nesting marsh wrens and yellow-headed blackbirds spatially segregated? The Auk 103:135-140.

Leyrer, J., Spaans, B., Camara, M., Piersma, T. 2006. Small home ranges and high site fidelity in red knots (*Calidris c. canutus*) wintering on the Banc d'Arguin, Mauritania. J Ornithol 147: 376–384.

Lima, A.P., Magnusson, W.E., Menin, M., Erdtmann, L.K., Rodrigues, D.J., Keller, C., Hodl, W. 2006. Guide to the frogs of Reserva Adolpho Ducke, Central Amazonia. Attema Design Editorial. Manaus, Brazil.

Linzey, A. V. 1983. *Synaptomys cooperi*. Mammalian Species 210:1-5.

Linzey, A.V. & NatureServe (Hammerson, G.). 2008. *Microtus pennsylvanicus*. IUCN Red List of Threatened Species. Version 2012.2. [online] URL: [www.iucnredlist.org](http://www.iucnredlist.org)

Linzey, A.V. & NatureServe (Hammerson, G.). 2008. *Oryzomys palustris*. IUCN Red List of Threatened Species. Version 2012.2. [online] URL: [www.iucnredlist.org](http://www.iucnredlist.org)

Litzgus, J.D., Brooks, R.J. 1998. Growth in a cold environment: body size and sexual maturity in a northern population of Spotted Turtles, *Clemmys* *guttata*. Canadian Journal of Zoology 76:773-782.

Litzgus, J.D., Mousseau, T.A. 2006. Geographic variation in reproduction in a freshwater turtle (*Clemmys guttata*). Herpetologica 62:132-140.

Llimona, F., del Hoyo, J. 1992. Family Podicipedidae. In del Hoyo, J., Elliott, A., Sargatal, J. Editors. Handbook of the birds of the world, vol. 1. Ostrich to Ducks. Lynx Edicions, Barcelona, Spain.

Lor, S.K. 2007. Habitat use and home range of American bitterns (*Botaurus lentiginosus*) and monitoring of inconspicuous marsh birds in northwest Minnesota. Ph.D. Thesis. University of Missouri, Columbia, Missouri, USA.

Lor, S., Malecki, R. A. 2006. Breeding ecology and nesting habitat associations of five marsh bird species in western New York. Waterbirds 29:427-436.

Lowther, J. K. 1977. Nesting biology of the sora at Vermilion, Alberta. Can. Field-Nat. 91:63-67.

Lowther, P.E. 1999. Alder flycatcher (*Empidonax alnorum*), The Birds of North America Online (A. Poole, Ed.). Ithaca: Cornell Lab of Ornithology; Retrieved from the Birds of North America Online: <http://bna.birds.cornell.edu.bnaproxy.birds.cornell.edu/bna/species/446>

Lowther, P. E., Celada, C., Klein, N.K., Rimmer, C.C., Spector, D.A. 1999. Yellow warbler (*Setophaga petechia*). The Birds of North America Online (A. Poole, Ed.). Ithaca: Cornell Lab of Ornithology; Retrieved from the Birds of North America Online: <http://bna.birds.cornell.edu.bnaproxy.birds.cornell.edu/bna/species/454>

Lowther, P.E., Douglas III, H.D., Gratto-Trevor, C.L. 2001. Willet (*Tringa semipalmata*). The Birds of North America Online (A. Poole, Ed.). Ithaca: Cornell Lab of Ornithology; Retrieved from the Birds of North America Online: <http://bna.birds.cornell.edu.bnaproxy.birds.cornell.edu/bna/species/579>

Lowther, P., Poole, A.F., Gibbs, J.P., Melvin, S., Reid, F.A. 2009. American bittern (*Botaurus lentiginosus*). The Birds of North America Online (A. Poole, Ed.). Ithaca: Cornell Lab of Ornithology; Retrieved from the Birds of North America Online: <http://bna.birds.cornell.edu.bnaproxy.birds.cornell.edu/bna/species/018>

MacCulloch, R. D. 2002. The ROM field guide to amphibians and reptiles of Ontario. The Royal Ontario Museum and McClelland & Stewart Ltd., Toronto, Ontario.

MacCulloch, R. D., Weller, W.F. 1988. Some aspects of reproduction in a Lake Erie population of Blanding's turtle, *Emydoidea blandingii*. Can. J. Zool. 66: 2317-2319.

Macwhirter, B., Austin-Smith Jr., P., Kroodsma, D. 2002. Sanderling (*Calidris alba*), The Birds of North America Online (A. Poole, Ed.). Ithaca: Cornell Lab of Ornithology; Retrieved from the Birds of North America Online: <http://bna.birds.cornell.edu.bnaproxy.birds.cornell.edu/bna/species/653>

Madge, S.C. 2006. Family Cisticolidae. In del Hoyo, J., Elliott, A., Christie, D.A. Editors. Handbook of the Birds of the World, Vol. 11. Old world flycatchers to old world warblers. Lynx Edicions, Barcelona, Spain.

Madge, S.C. 2008. Family Remizidae. In del Hoyo, J., Elliott, A., Christie, D.A. Editors. Handbook of the birds of the world, vol. 13. Penduline-tits to Shrikes. Lynx Edicions, Barcelona, Spain.

Madison, D. M., Farrand, L III. 1998. Habitat use during breeding and emigration in radio-implanted tiger salamanders, *Ambystoma tigrinum*. Copeia 1998:402-410.

la Marca, E., Azevedo-Ramos, C., Silvano, D., Scott, N., Aquino, L., Faivovich, J. 2004. *Hypsiboas raniceps*. IUCN Red List of Threatened Species. Version 2012.2. [online] URL: [www.iucnredlist.org](http://www.iucnredlist.org)

Matheu, E., del Hoyo, J. 1992. Family Threskiornithidae. In del Hoyo, J., Elliott, A., Sargatal, J. Editors. Handbook of the birds of the world, vol. 1. Ostrich to Ducks. Lynx Editions, Barcelona, Spain.

Martínez-Vilalta, A., Motis, A. 1992. Order Ciconiiformes. In del Hoyo, J., Elliott, A., Sargatal, J. Editors. Handbook of the Birds of the World, Vol. 1. Lynx Edicions, Barcelona, Spain.

Matthews, Jr., W. C. 1983. Home range, movements, and habitat selection of nesting gallinules in a Louisiana freshwater marsh. Master's Thesis. Louisiana State University, Baton Rouge, Louisiana, USA.

Matthews, K. R., Pope, K. L. 1999. A telemetric study of the movement patterns and habitat use of *Rana muscosa*, the mountain yellow-legged frog, in a high-elevation basin in Kings Canyon National Park, California. Journal of Herpetology 33:615-624.

Matthews, K. R., Miaud, C. 2007. A skeletochronological study of the age structure, growth, and longevity of the Mountain Yellow-legged Frog, *Rana muscosa*, in the Sierra Nevada, California. Copeia 2007:986-993.

Maxson, S.J., Fieberg, J.R., Riggs, M.R. 2007. Black tern nest habitat selection and factors affecting nest success in Northwestern Minnesota. Waterbirds 30:1-9.

Mccrimmon, Jr., D.A., Ogden, J.C., Bancroft, G.T. 2011. Great egret (*Ardea alba*), The Birds of North America Online (A. Poole, Ed.). Ithaca: Cornell Lab of Ornithology; Retrieved from the Birds of North America Online: <http://bna.birds.cornell.edu.bnaproxy.birds.cornell.edu/bna/species/570>

McRae, S.B. 2011. Conspecific brood parasitism in the tropics: an experimental investigation of host responses in common moorhens and American purple gallinules. Ecology and Evolution 1:317-329.

Meade, T. 2008. "*Lithobates sphenocephalus sphenocephalus*". Animal Diversity Web. [online] URL: <http://animaldiversity.ummz.umich.edu/accounts/Lithobates_sphenocephalus_sphenocephalus/>

van der Meijden, A., Cavagnaro, J. 2013. AmphibiaWeb: Information on amphibian biology and conservation. *Lissotriton helveticus*. Berkeley, California: AmphibiaWeb. [online] URL: <http://amphibiaweb.org/>

Melvin, S.M., Gibbs, J.P. 2012. Sora (*Porzana carolina*), The Birds of North America Online (A. Poole, Ed.). Ithaca: Cornell Lab of Ornithology; Retrieved from the Birds of North America Online: <http://bna.birds.cornell.edu.bnaproxy.birds.cornell.edu/bna/species/250>

Mészáros, A.L., Kajdocsi, S., Szentirmai, I., Komdeur, J., Székely, T. 2006. Breeding site fidelity in penduline tit *Remiz pendulinus* in Southern Hungary. European Journal of Wildlife Research 52:39-42.

Miaud, C., Sanuy, D. 2005. Terrestrial habitat preferences of the natterjack toad during and after the breeding season in a landscape of intensive agricultural activity. Amphibia-Reptilia 26:359-366.

Mijares, A., Rodrigues, M.T., Baldo, D. 2010. *Physalaemus cuvieri*. IUCN Red List of Threatened Species. Version 2012.2. [online] URL: [www.iucnredlist.org](http://www.iucnredlist.org)

Mikulíček, P., Pišút, P. 2012. Genetic structure of the marsh frog (*Pelophylax ridibundus*) populations in urban landscape. Eur J Wildl Res 58:833-845.

Millar, C.S. 2010. The spatial ecology of Blanding’s turtles (*Emydoidea blandingii*): from local movement patterns, home ranges and microhabitat selection to Ontario-wide habitat suitability modelling. M.Sc. Thesis. University of Ottawa, Ottawa, Ontario, Canada.

Millar, C.S., Blouin-Demers, G. 2011. Spatial ecology and seasonal activity of Blanding's turtle (*Emydoidea blandingii*) in Ontario, Canada. Journal of Herpetology. 45:370-378

Mingo, M. 2008. "*Anas discors*". Animal Diversity Web. [online] URL: <http://animaldiversity.ummz.umich.edu/accounts/Anas_discors/>

Mitchell, J.C. 2013.AmphibiaWeb: Information on amphibian biology and conservation. *Rana virgatipes*. Berkeley, California: AmphibiaWeb. [online] URL: <http://amphibiaweb.org/>

Mowbray, T.B. 1997. Swamp sparrow (*Melospiza georgiana*), The Birds of North America Online (A. Poole, Ed.). Ithaca: Cornell Lab of Ornithology; Retrieved from the Birds of North America Online: <http://bna.birds.cornell.edu.bnaproxy.birds.cornell.edu/bna/species/279>

Mowbray, T. 1999. American wigeon (*Anas americana*). The Birds of North America Online (A. Poole, Ed.). Ithaca: Cornell Lab of Ornithology; Retrieved from the Birds of North America Online: <http://bna.birds.cornell.edu.bnaproxy.birds.cornell.edu/bna/species/401>

Mueller, H. 1999. Wilson's snipe (*Gallinago delicata*), The Birds of North America Online (A. Poole, Ed.). Ithaca: Cornell Lab of Ornithology; Retrieved from the Birds of North America Online: <http://bna.birds.cornell.edu.bnaproxy.birds.cornell.edu/bna/species/417>

Mullarney, K., Svensson, L., Zetterström, D., Grant, P.J. 1999. Birds of Europe. Princeton University Press, Princeton, New Jersey, USA.

Muller, M.J., Storer, R.W. 1999. Pied-billed grebe (*Podilymbus podiceps*), The Birds of North America Online (A. Poole, Ed.). Ithaca: Cornell Lab of Ornithology; Retrieved from the Birds of North America Online: <http://bna.birds.cornell.edu.bnaproxy.birds.cornell.edu/bna/species/410>

Oldham, R. S. 1967. Orienting mechanisms of the green frog, *Rana clamitans*. Ecology 48:477-491.

Orta, J. 1994. Western marsh-harrier *Circus aeruginosus*. In del Hoyo, J., Elliott, A., Sargatal, J. eds. Handbook of the birds of the world. Volume 2. New world vultures to guineafowl. Lynx Edicions, Barcelona, Spain.

Owen, J.G. 1984. *Sorex fumeus*. Mammalian Species 215:1-8.

Paisley, R.N., Wetzel, J.F., Nelson, J.S., Stetzer, C., Hamernick, M.G., Anderson, B.P. 2009. Survival and spatial ecology of the snapping turtle, *Chelydra serpentina*, on the Upper Mississippi River. Canadian Field-Naturalist 123:329-337.

Pellet, J., Rechsteiner, L., Skrivervik, A. K., Zürcher, J-F., Perrin, N. 2006. Use of the harmonic direction finder to study the terrestrial habitats of the European tree frog (*Hyla arborea*). Amphibia-Reptilia 27:138-142.

van Perlo, B. 2009. A field guide to the birds of Brazil. Oxford University Press, New York, New York, USA.

Petirs, B. 2013. AmphibiaWeb: Information on amphibian biology and conservation. *Ambystoma mavortium*. Berkeley, California: AmphibiaWeb. [online] URL: <http://amphibiaweb.org/>

Petokas, P.J., Alexander, M.M. 1980. The nesting of *Chelydra serpentina* in northern New York. Journal of Herpetology 14:239-244

Petranka, J.W. 2010. Salamanders of the United States and Canada. Smithsonian Books. Washington, D.C.

Petry, M.V., Da Silva Fonseca, V.S. 2005. Breeding success of the colonist species *Bubulcus ibis* (Linnaeus, 1758) and four native species. Acta Zoologica 86:217-221.

Pickens, B.A. 2012. Modeling the spatial and temporal dynamics of coastal marsh birds. Ph.D. Thesis. Louisiana State University, Baton Rouge, Louisiana, USA.

Pearson, D.J. 2006. Family Sylviidae. In del Hoyo, J., Elliott, A., Christie, D.A. Editors. Handbook of the Birds of the World, Vol. 11. Old world flycatchers to old world warblers. Lynx Edicions, Barcelona, Spain.

Pfennig, K.S., Pfennig, D.W. 2005. Character displacement as the "best of a bad situation": fitness trade-offs resulting from selection to minimize resource and mate competition. Evolution 59:2200-2208.

Pierce, R.J. 1996. Family Recurvirostridae. In del Hoyo, J., Elliott, A., Sargatal, J. Editors. Handbook of the Birds of the World, Vol. 3. Hoatzin to Auks. Lynx Edicions, Barcelona, Spain.

Pierluissi, S. 2006. Breeding waterbird use of rice fields in southwestern Louisiana. M.Sc. Thesis. Louisiana State University, Baton Rouge, Louisiana, USA.

Pilliod, D.S., Fronzuto, J.A. 2013. AmphibiaWeb: Information on amphibian biology and conservation. *Ambystoma macrodactylum*. Berkeley, California: AmphibiaWeb. [online] URL: <http://amphibiaweb.org/>

Pilliod, D.S., Peterson, C.R., Ritson, P.I., 2002. Seasonal migration of Columbia spotted frogs (*Rana luteiventris*) among complementary resources in a high mountain basin. Canadian Journal of Zoology 80:1849-1862.

Plummer, M. V. 1992. Relationships among mothers, litters, and neonates in diamondback water snakes (*Nerodia rhombifer*). Copeia 1992:1096-1098.

Pogány, A., Van Dijk , R.E., Horváth , P., Székely, T. 2012. Parental behavior and reproductive output in male-only cared and female-only cared clutches in the Eurasian penduline tit (*Remiz pendulinus*). The Auk 129:773-781.

Ponsero, A., Joly, A. 1998. Clutch size, egg survival and migration distance in the agile frog (*Rana dalmatina*) in a floodplain. Arch.Hydrobiol. 142:343-352.

Poole, A.F., Bevier, L. R., Marantz, C. A., Meanley, B. 2005. King rail (*Rallus* *elegans*). The Birds of North America Online (A. Poole, Ed.). Ithaca: Cornell Lab of Ornithology; Retrieved from the Birds of North America Online: <http://bna.birds.cornell.edu.bnaproxy.birds.cornell.edu/bna/species/003>

Poole, A.F., Lowther, P., Gibbs, J. P., Reid, F. A., Melvin, S. M. 2009. Least bittern (*Ixobrychus exilis*). The Birds of North America Online (A. Poole, Ed.). Ithaca: Cornell Lab of Ornithology; Retrieved from the Birds of North America Online: <http://bna.birds.cornell.edu.bnaproxy.birds.cornell.edu/bna/species/017>

Prado, C.P.A., Haddad, C.F.B. 2005. Size-fecundity relationships and reproductive investment in female frogs in the Pantanal, south-western Brazil. Herpetological Journal 15:181-189.

Prado, C.P.A., Uetanabaro, M., Lopes, F.S. 2000. Reproductive strategies of *Leptodactylus chaquensis* and *L. podicipinus* in the Pantanal, Brazil. Journal of Herpetology 34:135-139.

Pratt, H. M., Winkler, D.W. 1985. Clutch size, timing of laying and reproductive success in a colony of great blue herons and great egrets. The Auk 102:49-63.

Pretelli, M.G., Josens, M.L., Escalante, A.H. 2012. Breeding biology at a mixed-species colony of great egret and cocoi heron in a pampas wetland of Argentina. Waterbirds 35:35-43.

Prosper, J., Hafner, H. 1996. Breeding aspects of the colonial Ardeidae in the Albufera de Valencia, Spain: population changes, phenology, and reproductive success of the three most abundant species. Colonial Waterbirds 19 (Special Publication 1):98-107.

Provete, D.B. 2013a. AmphibiaWeb: Information on amphibian biology and conservation. *Dermatonotus muelleri*. Berkeley, California: AmphibiaWeb. [online] URL: <http://amphibiaweb.org/>

Provete, D.B. 2013b. AmphibiaWeb: Information on amphibian biology and conservation. *Physalaemus centralis*. Berkeley, California: AmphibiaWeb. [online] URL: <http://amphibiaweb.org/>

Pugh, S.R., Johnson, S., Tamarin, R.H. 2003. Voles *Microtus* species. In Feldhamer, G.A., Thompson, B.C., Chapman, J.A., Eds. Wild mammals of North America. Biology, conservation and management. 2^nd^ e.d. The John Hopkins University Press, Baltimore, Maryland, USA.

Reaser, J.K., Pilliod, D.S. 2013. AmphibiaWeb: Information on amphibian biology and conservation. *Rana luteiventris*. Berkeley, California: AmphibiaWeb. [online] URL: <http://amphibiaweb.org/>

Rebelo, R., Leclair, M.H. 2003. Differences in size at birth and brood size among Portuguese populations of the fire salamander, *Salamandra salamandra*. Herpetological Journal 13:179-187.

Rehfisch, M.M., Clark, N.A., Langston, R.H.W., Greenwood J.J.D. 1996. A guide to the provision of refuges for waders: an analysis of 30 years of ringing data from the Wash, England. Journal of Applied Ecology 33:673-687.

Rehfisch, M.M., Insley, H., Swann, B. 2003. Fidelity of overwintering shorebirds to roosts on the Moray Basin, Scotland: implications for predicting impacts of habitat loss. Ardea 91:53-70.

Reich, L.M. 1981. *Microtus pennsylvanicus*. Mammalian Species 15:1-8.

Reichle, S., Aquino, L., Colli, G., Silvano, D., Azevedo-Ramos, C., Bastos, R. 2004. *Dendropsophus nanus*. IUCN Red List of Threatened Species. Version 2012.2. [online] URL: [www.iucnredlist.org](http://www.iucnredlist.org)

Reisman, E. 2013. AmphibiaWeb: Information on amphibian biology and conservation. *Leptodactylus mystacinus*. Berkeley, California: AmphibiaWeb. [online] URL: <http://amphibiaweb.org/>

Resetarits, Jr., W.J., Wilbur, H.M. 1989. Choice of oviposition site by *Hyla chrysoscelis*: role of predators and competitors. Ecology 70:220-228.

Reynolds, R., Caramaschi, U., Mijares, A., Acosta-Galvis, A., Heyer, R., Lavilla, E., Hardy, J. 2004. *Leptodactylus fuscus*. IUCN Red List of Threatened Species. Version 2012.2. [online] URL: [www.iucnredlist.org](http://www.iucnredlist.org)

Richmond, A.M. 1999. Contributions to the herpetology of New England. Ph.D. Thesis. University of Massachusetts, Amherst, Massachusetts, USA.

Ritke, M.E., Babb, J.G., Ritke, M.K. 1990. Life history of the gray treefrog (*Hyla chrysoscelis*) in western Tennessee. Journal of Herpetology 24:135-141.

Rittenhouse, T.A.G., Semlitsch, R.D. 2009. Behavioral response of migrating wood frogs to experimental timber harvest surrounding wetlands. Can. J. Zool. 87:618–625.

Robinson, J.A., Oring, L.W., Skorupa, J.P., Boettcher, R. 1997. American avocet (*Recurvirostra* *americana*). The Birds of North America Online (A. Poole, Ed.). Ithaca: Cornell Lab of Ornithology; Retrieved from the Birds of North America Online: <http://bna.birds.cornell.edu.bnaproxy.birds.cornell.edu/bna/species/275>

Robinson, J.A., Reed, J.M., Skorupa, J.P., Oring, L.W. 1999. Black-necked stilt (*Himantopus mexicanus*). The Birds of North America Online (A. Poole, Ed.). Ithaca: Cornell Lab of Ornithology; Retrieved from the Birds of North America Online: <http://bna.birds.cornell.edu.bnaproxy.birds.cornell.edu/bna/species/449>

Robinson, K.M., Murphy, G.G. 1978. The reproductive cycle of the eastern spiny softshell turtle (*Trionyx spiniferus spiniferus*). Herpetologica 34:137-140.

Robson, C. 2000. A guide to the birds of southeast Asia. Princeton University Press, Princeton, New Jersey, USA.

Rodrigues, D.J., Uetanabaro, M., Lopes, F.S. 2005. Reproductive patterns of *Trachycephalus venulosus* (Laurenti, 1768) and *Scinax fuscovarius* (Lutz, 1925) from the Cerrado, Central Brazil. Journal of Natural History 39:3217-3226.

Rodrigues, M.T., Telles, A.M. 2010. *Scinax similis*. IUCN Red List of Threatened Species. Version 2012.2. [online] URL: [www.iucnredlist.org](http://www.iucnredlist.org)

Roe, J.H., Georges, A. 2008. Terrestrial activity, movements and spatial ecology of an Australian freshwater turtle, *Chelodina longicollis*, in a temporally dynamic wetland system. Austral Ecology 33:1045–1056.

Roe, J.H., Kingsbury, B.A., Herbert, N.R. 2004. Comparative water snake ecology: conservation of mobile animals that use temporally dynamic resources. Biological Conservation 118:79–89.

Rohwer, F.C., Johnson, W.P., Loos, E.R. 2002. Blue-winged teal (*Anas discors*), The Birds of North America Online (A. Poole, Ed.). Ithaca: Cornell Lab of Ornithology; Retrieved from the Birds of North America Online: <http://bna.birds.cornell.edu.bnaproxy.birds.cornell.edu/bna/species/625>

Rorabaugh, J.C., Lannoo, M.J. 2013. AmphibiaWeb: Information on amphibian biology and conservation. *Pseudacris regilla*. Berkeley, California: AmphibiaWeb. [online] URL: <http://amphibiaweb.org/>

Rose, F.L., Armentrout, D. 1976. Adaptive strategies of *Ambystoma tigrinum* Green inhabiting the Llano Estacado of west Texas. Journal of Animal Ecology 45:713-729.

Rosenthal, A. 2004. "*Agelaius phoeniceus*". Animal Diversity Web. [online] URL: <http://animaldiversity.ummz.umich.edu/site/accounts/information/Agelaius_phoeniceus.html>

Roth, T.C., Greene, B.D. 2006. Movement patterns and home range use of the northern watersnake (*Nerodia sipedon*). Copeia 3:544–551.

Rowe, J. W. 2003. Activity and movements of midland painted turtles (*Chrysemys picta marginata*) living in a small marsh system on Beaver Island, Michigan. Journal of Herpetology 37:342-353.

Rowe, J. W., Coval, K. A., Campbell, K.C. 2003. Reproductive characteristics of female midland painted turtles (*Chrysemys picta marginata*) from a population on Beaver Island, Michigan. Copeia 2003:326-336.

Russell, A. P., Bauer, A.M., Lynch, W. 2000. The amphibians and reptiles of Alberta: a field guide and primer of boreal herpetology, second edition. University of Calgary Press, Calgary, Alberta, Canada.

Ryan, M.J. 1983. Sexual selection and communication in a Neotropical frog *Physalaemus pustulosus*. Evolution 37:261-272.

Ryan, M.R., Renken, R.B. 1987. Habitat use by breeding willets in the northern Great Plains. Wilson Bull. 99:175-189.

Ryder, R.A., Manry, D.E. 1994. White-faced ibis (*Plegadis chihi*). The Birds of North America Online (A. Poole, Ed.). Ithaca: Cornell Lab of Ornithology; Retrieved from the Birds of North America Online: <http://bna.birds.cornell.edu.bnaproxy.birds.cornell.edu/bna/species/130>

Samollow, P.B. 1980. Selective mortality and reproduction in a natural population of *Bufo boreas*. Evolution 34:18-39.

Schaefer, E.F., Hamann, M.I., Kehr, A.I., González, C.E., Duré, M.I. 2006. Trophic, reproductive and parasitological aspects of the ecology of *Leptodactylus chaquensis* (Anura: Leptodactylidae) in Argentina. Herpetological Journal 16:387-394.

Schooley, R. L., Branch, L. C. 2006. Space use by roundtailed muskrats in isolated wetlands. Journal of Mammalogy 87:495-500.

Schulte, U. Daniel Küsters, D, Steinfartz, S. 2007. A PIT tag based analysis of annual movement patterns of adult fire salamanders (*Salamandra* *salamandra* ) in a middle European habitat. Amphibia-Reptilia 28:531-536.

Semlitsch, R.D., Bodie, J.R. 2003. Biological criteria for buffer zones around wetlands and riparian habitats for amphibians and reptiles. Conserv. Biol. 17:1219-1228.

Sherman, C.D.H., Uller, T., Wapstra, E., Olsson, M. 2008. Within-population variation in ejaculate characteristics in a prolonged breeder, Peron*’*s tree frog, *Litoria peronii*. Naturwissenschaften 95:1055–1061.

Shirose, L.J., Brooks, R.J. 1995. Age structure, mortality, and longevity in syntopic populations of three species of ranid frogs in central Ontario. Can. J. Zool. 73:1878-1886.

Silva, W.R., Giaretta, A.A., Facure, K.G. 2005. On the natural history of the South American pepper frog, *Leptodactylus labyrinthicus* (Spix, 1824) (Anura: Leptodactylidae). Journal of Natural History 39:555-566.

Simmons, K.E.L. 1956. Territory in the little ringed plover *Charadrius dubius*. Ibis 98:390-397.

Sinsch, U. 1988. Seasonal changes in the migratory behaviour of the toad *Bufo bufo*: direction and magnitude of movements. Oecologia 76:390–398.

Sinsch, U. 2007. Initial orientation of newts (*Triturus vulgaris, T.cristatus*) following short–and long–distance displacements. Ethology Ecology & Evolution 19:201-214.

Smith, K.G., Wittenberg, S.R., Macwhirter, R.B., Bildstein, K.L. 2011. Northern harrier (*Circus cyaneus*), The Birds of North America Online (A. Poole, Ed.). Ithaca: Cornell Lab of Ornithology; Retrieved from the Birds of North America Online: <http://bna.birds.cornell.edu.bnaproxy.birds.cornell.edu/bna/species/210>

Spellerberg, I.F. 2002. Amphibians and reptiles of north-west Europe: their natural history, ecology and conservation. Science Publishers, Inc. Enfield, New Hampshire, USA.

Spencer, A. W., 1964. The relationship of dispersal and migration to gene flow in the Boreal chorus frog. Ph.D. thesis. Colorado State University, Fort Collins, Colorado, USA.

Steen, D.A., Gibbs, J.P. 2004. Effects of roads on the structure of freshwater turtle populations. Conserv. Biol. 18:1143-1148.

Steen, D.A., Smith, L.L., Miller, G.J., Sterrett, S.C. 2006. Post-breeding terrestrial movements of *Ambystoma* *tigrinum* (eastern tiger salamanders). Southeastern Naturalist 5:285-288.

Stolen, E.D., Collazo, J.A., Percival, H. F. 2007. Scale-dependent habitat selection of nesting great egrets and snowy egrets. Waterbirds 30:384-393.

Svensson, B.W. 1987. Structure and vocalizations of display flights in the broad-billed sandpiper *Limicola falcinellus*. Ornis Scandinavica 18:47-52.

Tacha, T. C., Nesbitt, S.A., Vohs, P.A. 1992. Sandhill crane (*Grus canadensis*), The Birds of North America Online (A. Poole, Ed.). Ithaca: Cornell Lab of Ornithology; Retrieved from the Birds of North America Online: <http://bna.birds.cornell.edu.bnaproxy.birds.cornell.edu/bna/species/031>

Taft, O. W., Sanzenbacher, P.M., Haig, S.M. 2008. Movements of wintering Dunlin *Calidris alpina* and changing habitat availability in an agricultural wetland landscape. Ibis 150:541-549.

Taylor, P.B. 1996. Family Rallidae. In del Hoyo, J., Elliott, A., Sargatal, J. Editors. Handbook of the birds of the world, vol. 3. Hoatzin to Auks. Lynx Edicions, Barcelona, Spain.

Telfair II, R.C. 2006. Cattle egret (*Bubulcus ibis*). The Birds of North America Online (A. Poole, Ed.). Ithaca: Cornell Lab of Ornithology; Retrieved from the Birds of North America Online: <http://bna.birds.cornell.edu.bnaproxy.birds.cornell.edu/bna/species/113>

Thiollay, J.M. 1994. Family Accipitridae. In del Hoyo, J., Elliott, A., Sargatal, J. Editors. Handbook of the Birds of the World, Vol. 2. New world vultures to gunieafowl. Lynx Edicions, Barcelona, Spain.

Tibbitts, T. L., Moskoff, W. 1999. Lesser yellowlegs (*Tringa flavipes*). The Birds of North America Online (A. Poole, Ed.). Ithaca: Cornell Lab of Ornithology; Retrieved from the Birds of North America Online: <http://bna.birds.cornell.edu.bnaproxy.birds.cornell.edu/bna/species/427>

Tran, S.L., Moorhead, D.L., McKenna, K.C. 2007. Habitat selection by native turtles in a Lake Erie wetland, USA. American Midland Naturalist 158:16–28.

Trauth, S.E. 2013. *Ambystoma texanum*. AmphibiaWeb: Information on amphibian biology and conservation. Berkeley, California. [online] URL: <http://amphibiaweb.org/>

Tucker, J.K. 2001. Clutch frequency in the red-eared slider (*Trachemys scripta elegans*). Journal of Herpetology 35:664-668.

Tucker, J.K., Paukstis, G.L., Janzen, F.J. 1998. Annual and local variation in reproduction in the red-eared slider, *Trachemys scripta elegans*. Journal of Herpetology 32:515-526.

Turner, J.R. 2004. Frogs of Australia. Pensoft Publishers. Sofia, Bulgaria.

Twedt, D.J., Crawford, R.D. 1995. Yellow-headed blackbird (*Xanthocephalus xanthocephalus*), The Birds of North America Online (A. Poole, Ed.). Ithaca: Cornell Lab of Ornithology; Retrieved from the Birds of North America Online: <http://bna.birds.cornell.edu.bnaproxy.birds.cornell.edu/bna/species/192>

Tyler, M.J., Knight, F. 2009. Field guide to the frogs of Australia. CSIRO Publishing. Collingwood, Victoria, Australia.

Urban, E.K., Fry, C.H., Keith, S., eds. 1997. The birds of Africa. Vol. V. Academic Press, San Diego, California, USA.

Vennesland, R.G., Butler, R.W. 2011. Great blue heron (*Ardea herodias*). The Birds of North America Online (A. Poole, Ed.). Ithaca: Cornell Lab of Ornithology; Retrieved from the Birds of North America Online: <http://bna.birds.cornell.edu.bnaproxy.birds.cornell.edu/bna/species/025>

Verner, J. 1965. Breeding biology of the long-billed marsh wren. Condor 67:6-30.

van Vessem, J., Draulaks, D., de Bont, A.F. 1984. Movements of radio-tagged grey herons *Ardea cinerea* during the breeding season in a large pond area. Ibis 126:576-587.

Vonesh, J.R., de la Cruz, O. 2002. Complex life cycles and density dependence: assessing the contribution of egg mortality to amphibian declines. Oecologia 133:325-333.

Vredenburg, V., Fellers, G.M., Davidson, C. 2013. AmphibiaWeb: Information on amphibian biology and conservation. *Rana muscosa*. Berkeley, California: AmphibiaWeb. [online] URL: <http://amphibiaweb.org/>

Warburg, M., Degani, G., Warburg, I. 1979. Growth and population structure of *Salamandra salamandra* (L.) larvae in different limnological conditions. Hydrobiologia 64:147-155.

Warnock, N.D., Gill, R.E. 1996. Dunlin (*Calidris alpina*). The Birds of North America Online (A. Poole, Ed.). Ithaca: Cornell Lab of Ornithology; Retrieved from the Birds of North America Online: <http://bna.birds.cornell.edu.bnaproxy.birds.cornell.edu/bna/species/203>

Weinstein, B. 1999. "*Sorex fumeus*". Animal Diversity Web. [online] URL: <http://animaldiversity.ummz.umich.edu/accounts/Sorex_fumeus/>

West, R.L., Hess, G.K. 2002. Purple gallinule (*Porphyrio martinicus*). The Birds of North America Online (A. Poole, Ed.). Ithaca: Cornell Lab of Ornithology; Retrieved from the Birds of North America Online: <http://bna.birds.cornell.edu.bnaproxy.birds.cornell.edu/bna/species/626>

Westgate, M.J., Driscoll, D.A., Lindenmayer, D.B. 2012. Limited influence of stream networks on the terrestrial movements of three wetland-dependent frog species. Biological Conservation 153:169-176.

Whillans, T.H., Crossman, E.J. 1977. Morphological parameters and spring activities in a central Ontario population of midland painted turtle, *Chrysemys picta marginata* (Agassiz). Can Field Nat. 91:45-47.

Whiting, A.V. 2010. Factors affecting larval growth and development of the boreal chorus frog *Pseudacris maculata*. Ph.D. Thesis. University of Alberta, Edmonton, Alberta, Canada.

Whitlock, A.L. 2002. Ecology and status of the bog turtle (*Clemmys muhlenbergii*) in New England. Ph.D Thesis. University of Massachusetts, Amherst, Massachusetts, USA.

Wiersma, P. 1992. Family Charadriidae. In del Hoyo, J., Elliott, A., Sargatal, J. Editors. Handbook of the Birds of the World, Vol. 3. Lynx Edicions, Barcelona, Spain.

Wilson, J.N., Bekessy, S., Parris, K.M., Gordon, A., Heard, G.W., Wintle, B.A. 2012. Impacts of climate change and urban development on the spotted marsh frog (*Limnodynastes tasmaniensis*). Austral Ecology DOI: 10.1111/j.1442-9993.2012.02365.x

Woodward, B. 1987. Intra- and interspecific variation in spadefoot toad (*Scaphiopus*) clutch parameters. The Southwestern Naturalist 32:127-131.

Wong, L.C., Corlett, R.T., Young. L., Lee, J.S.Y. 1999. Foraging flights of nesting egrets and herons at a Hong Kong egretry, south China. Waterbirds 22:424-434.

Yasukawa, K., Searcy, W.A. 1995. Red-winged blackbird (*Agelaius phoeniceus*). The Birds of North America Online (A. Poole, Ed.). Ithaca: Cornell Lab of Ornithology; Retrieved from the Birds of North America Online: <http://bna.birds.cornell.edu.bnaproxy.birds.cornell.edu/bna/species/184>
